# Supplementary material for: Clonal Heterogeneity Influences the Fate of New Adaptive Mutations
Source: Cell Rep. 2017 Oct 17;21(3):732–44. doi: 10.1016/j.celrep.2017.09.046 (PMC5656752; doi:10.1016/j.celrep.2017.09.046)
Supplement: Document S2. Article plus Supplemental Information [file mmc2.pdf]

Cell Reports

Article

Clonal Heterogeneity Influences the Fate of New Adaptive Mutations

Graphical Abstract

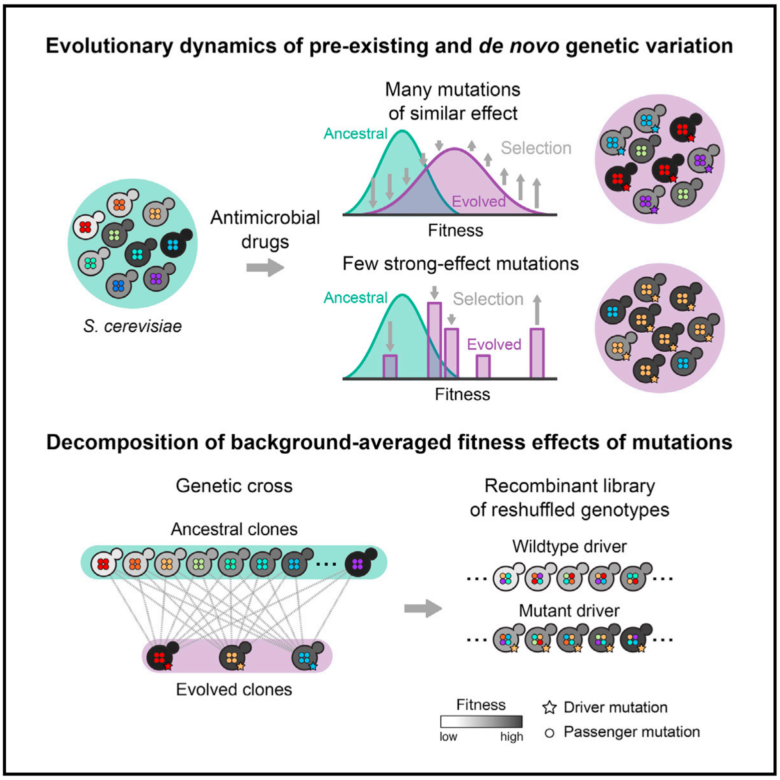

Highlights

- Clonal heterogeneity influences the acquisition of antimicrobial resistance
- Joint role of pre-existing and *de novo* genetic variation in clonal evolution
- Clonal dynamics are shaped by background-dependent fitness effects of mutations
- Loss of clonal heterogeneity is balanced by genomic instability and diversification

Authors

Ignacio Vázquez-García, Francisco Salinas, Jing Li, ..., Jonas Warringer, Ville Mustonen, Gianni Liti

Correspondence

ivg@sanger.ac.uk (I.V.-G.), v.mustonen@helsinki.fi (V.M.), gianni.liti@unice.fr (G.L.)

In Brief

Vázquez-García et al. examine the role of clonal heterogeneity in the acquisition of antimicrobial resistance. They report that pre-existing and *de novo* genetic variation jointly contribute to clonal evolution. By building a library of adaptive mutations in multiple genetic backgrounds, they resolve the fitness effects of mutations in a clonal lineage.

Vázquez-García et al., 2017, Cell Reports *21*, 732–744
October 17, 2017 © 2017 The Author(s).
https://doi.org/10.1016/j.celrep.2017.09.046

OPEN ACCESS
CellPress

Cell Reports
Article

Clonal Heterogeneity Influences the Fate of New Adaptive Mutations

Ignacio Vázquez-García,[1,2,9,*] Francisco Salinas,[3,6] Jing Li,[3] Andrej Fischer,[1] Benjamin Barré,[3] Johan Hallin,[3] Anders Bergström,[1,3] Elisa Alonso-Perez,[4] Jonas Warringer,[4,5] Ville Mustonen,[1,7,8,*] and Gianni Liti[3,8,*]
[1]Wellcome Trust Sanger Institute, Hinxton, Cambridge CB10 1SA, UK
[2]Department of Applied Mathematics and Theoretical Physics, University of Cambridge, Cambridge CB3 0WA, UK
[3]Université Côte d'Azur, INSERM, CNRS, IRCAN, 06107 Nice, France
[4]Department of Chemistry and Molecular Biology, University of Gothenburg, Gothenburg, Sweden
[5]Centre for Integrative Genetics (CIGENE), Department of Animal and Aquacultural Sciences, Norwegian University of Life Sciences, Ås, Norway
[6]Present address: Millennium Nucleus for Fungal Integrative and Synthetic Biology (MN-FISB), Departamento de Genética Molecular y Microbiología, Facultad de Ciencias Biológicas, Pontificia Universidad Católica de Chile, Casilla 114-D, Santiago, Chile
[7]Present address: Department of Biosciences, Department of Computer Science, Institute of Biotechnology, University of Helsinki, PO Box 65, 00014 Helsinki, Finland
[8]Senior author
[9]Lead Contact
*Correspondence: ivg@sanger.ac.uk (I.V.-G.), v.mustonen@helsinki.fi (V.M.), gianni.liti@unice.fr (G.L.)
https://doi.org/10.1016/j.celrep.2017.09.046

SUMMARY

The joint contribution of pre-existing and *de novo* genetic variation to clonal adaptation is poorly understood but essential to designing successful antimicrobial or cancer therapies. To address this, we evolve genetically diverse populations of budding yeast, *S. cerevisiae*, consisting of diploid cells with unique haplotype combinations. We study the asexual evolution of these populations under selective inhibition with chemotherapeutic drugs by time-resolved whole-genome sequencing and phenotyping. All populations undergo clonal expansions driven by *de novo* mutations but remain genetically and phenotypically diverse. The clones exhibit widespread genomic instability, rendering recessive *de novo* mutations homozygous and refining pre-existing variation. Finally, we decompose the fitness contributions of pre-existing and *de novo* mutations by creating a large recombinant library of adaptive mutations in an ensemble of genetic backgrounds. Both pre-existing and *de novo* mutations substantially contribute to fitness, and the relative fitness of pre-existing variants sets a selective threshold for new adaptive mutations.

INTRODUCTION

The adaptive response of a cell population can thwart therapeutic control of a wide spectrum of diseases, from bacterial and viral infections to cancer. A prototypical scenario arises when individuals in a population acquire heritable genetic or non-genetic changes to adapt and thrive in a new environment (Balaban et al., 2004; Marusyk et al., 2014; Toprak et al., 2011). Since the seminal findings by Luria and Delbrück (1943) that phage-resistant bacteria can acquire adaptive mutations prior to selection, measuring the fitness effects and dynamics of mutations has been key to map the principles of evolutionary adaptation (Barrick and Lenski, 2013). The focus has typically been on characterizing few mutations at a time under the implicit assumption that beneficial mutations are rare, treating pre-existing and acquired mutations separately. However, many mutations are often simultaneously present in a population, which result in fitness differences between individuals upon which selection can act (Lang et al., 2013; Levy et al., 2015; Parts et al., 2011; Venkataram et al., 2016).

Given that mutations in asexual populations are physically linked in the genome, the fates of pre-existing and *de novo* mutations are mutually dependent, and selection can only act on these sets of variants in their entirety. Genome evolution experiments on isogenic populations have revealed both adaptive sweeps and pervasive clonal competition in large populations where the mutation supply is high. This phenomenon, known as clonal interference, takes place as mutations in different individuals cannot recombine via sexual reproduction and is now relatively well understood both experimentally and theoretically (Gerrish and Lenski, 1998; Lang et al., 2013; Neher, 2013). Experiments on populations with extensive genetic variation have demonstrated that beneficial mutations expand in a repeatable way (Parts et al., 2011). Theory predicts that the rate of adaptation is proportional to the fitness variance present in a population, generating a traveling fitness wave (Desai and Fisher, 2007; Rouzine and Coffin, 2005). However, the role of *de novo* mutations has been negligible in these experiments, either because of their short duration or related to the selective constraints used. A study that was able to anticipate new mutations found that one or few genetic variants were sufficient to affect the fate of subsequent beneficial mutations, hinting that the joint dynamics of new mutations have to be considered in the light

 © 2017 The Author(s).
This is an open access article under the CC BY license (http://creativecommons.org/licenses/by/4.0/).

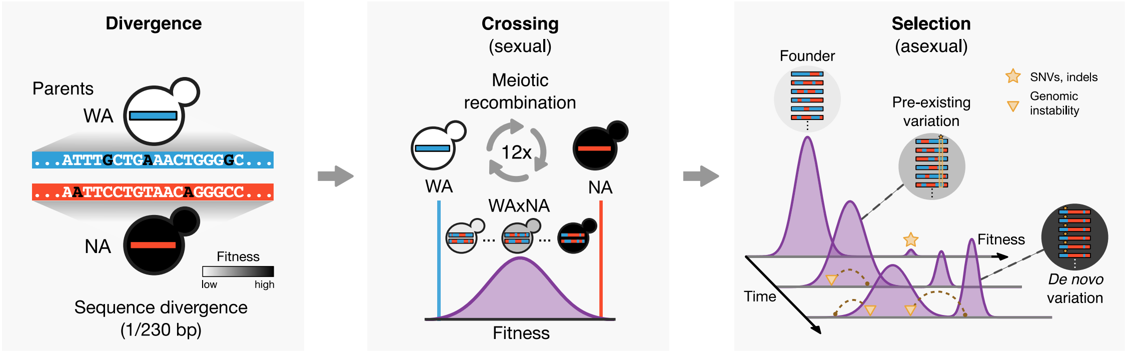

Figure 1. Study Overview

Schematic of the divergence, crossing, and selection phases of the experiment. Two diverged *S. cerevisiae* lineages (WA and NA) were crossed for twelve rounds, generating a large ancestral population of unique haplotypes. These diploid cells were asexually evolved for 32 days in stress and control environments, and their adaptation was studied by whole-population and isolate sequencing and phenotyping. Populations evolved resistant macroscopic subclones driven by individual cells with beneficial genetic backgrounds (i.e., parental allele configurations) and by beneficial *de novo* mutations that provided a resistance phenotype.

of pre-existing variation (Lang et al., 2011). The ensuing interaction between existing and subsequent mutations has been theoretically considered under different population genetic scenarios (Good et al., 2012; Hermisson and Pennings, 2005; Orr and Betancourt, 2001; Peter et al., 2012; Schiffels et al., 2011). A key theoretical prediction is that a new beneficial mutation will only establish when it has a selective advantage greater than a characteristic value that depends on the underlying fitness distribution (Good et al., 2012; Schiffels et al., 2011). However, this important hypothesis remains to be tested; namely, whether genetic diversity can change the evolutionary fate of new adaptive mutations by limiting the number of backgrounds where they can still outcompete the fittest extant individuals. Understanding the effect of genetic heterogeneity on adaptive dynamics is particularly urgent because recent findings indicate that it can play a major role in the development of resistant bacterial infections (Lieberman et al., 2014) and in cancer recurrence (Gerlinger et al., 2012; Landau et al., 2013).

We have delineated two lines of enquiry into our hypothesis. To what extent can the adaptive response be attributed to genetic variation already present in a population, and how much to acquired? How do the aggregate effects of pre-existing variation influence the fate of new mutations? To address these questions, we investigated the interaction between pre-existing (or background) genetic variation and new mutations in a population of diploid cells with unique combinations of alleles. The cells originate from two diverged *S. cerevisiae* strains (Figure 1). We carried out 12 rounds of random mating and sporulation (meiosis) between DBVPG6044, a West African (WA) palm wine strain, and YPS128, a North American (NA) oak tree bark strain (Parts et al., 2011). The cross population (WAxNA) consisted of 10^7–10^8 unique haplotypes, with a pre-existing single-nucleotide variant segregating every 230 bp on average. We further identified 91 *de novo* single-nucleotide variants (SNVs) and small insertions or deletions (indels) acquired during the crossing phase from genome sequences of 173 founder individuals. This is consistent with a mutation rate of approximately 2.89×10^{-10} mutations per base per generation, close to empirical estimates in other yeast strains (Zhu et al., 2014). We also observed aneuploidy in chromosome IX, indicating the presence of variation other than point mutations. This design results in the frequency spectrum of background mutations to be normally distributed so that pre-existing variants are already established and do not need to overcome genetic drift. We refer to the parental genotype of each individual in the cross as its genetic background, which, on average, differs by ~31,000 SNVs between individuals. Because naturally occurring deleterious mutations have been selected against over long evolutionary timescales, the recombinant parental genotypes are enriched for functional diversity that is not readily accessible using other techniques, such as random or site-directed mutagenesis. The cross-based approach also reduces genetic linkage of nearby loci, which enables us to localize background alleles responding to selection.

Starting from WA, NA, and WAxNA founders, we asexually evolved populations of ~10^7 cells in serial batch culture under drug inhibition with hydroxyurea (HU) and rapamycin (RM) at concentrations impeding, but not ending, cell proliferation. These drugs were chosen for having known targets and to cover two of the most common modes of action of antimicrobial and chemotherapy drugs: inhibition of nucleic acid synthesis (hydroxyurea) and inhibition of protein synthesis and cell growth (rapamycin). We derived replicate lines of WA, NA (2 each in hydroxyurea and rapamycin), and WAxNA (6 in hydroxyurea, 8 in rapamycin, and 4 in a control environment), propagating them for 32 days in 48-hr cycles (~54 generations; Experimental Procedures). We monitored evolutionary changes by whole-genome sequencing of populations after 2, 4, 8, 16, and 32 days as well as clonal isolates at 0 and 32 days (Table S1). Finally, we measured the rate of growth at the initial and final time points for a subset of populations and quantified the relative fitness contributions of background and *de novo* variation using a genetic cross.

RESULTS

Two regimes of selection became readily apparent in both sequence and phenotype. Initially, there were local changes in

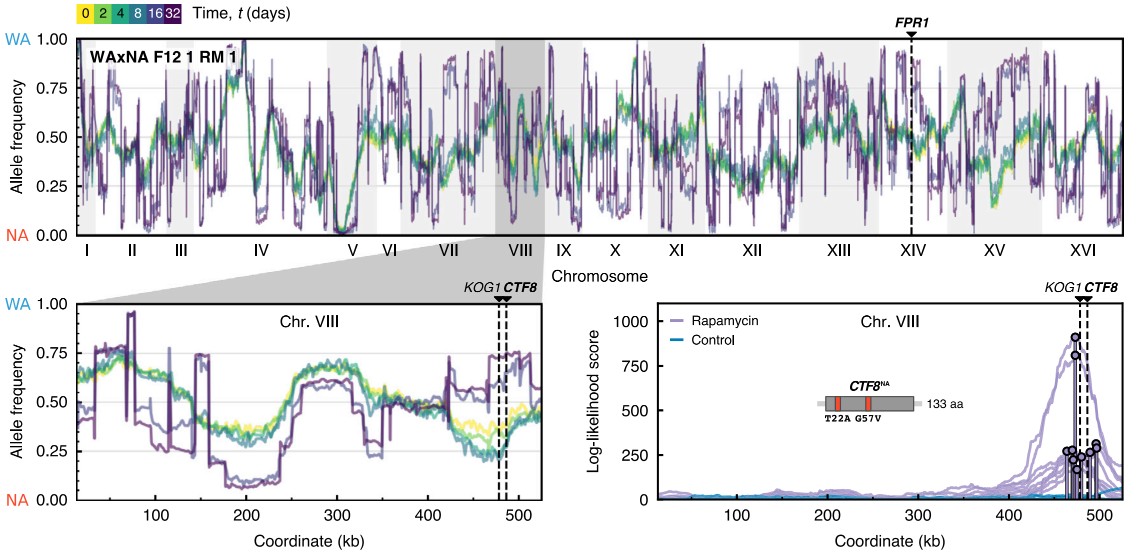

Figure 2. Genome-wide Allele Frequency Changes

Genome-wide allele frequency of pre-existing parental variants after $t = (0, 2, 4, 8, 16, 32)$ days, measured by whole-population sequencing for a representative population in rapamycin. Pre-existing and *de novo* driver mutations are highlighted by dashed lines. Top: chromosomes are shown on the x axis; the frequency of the WA allele at locus i, x_i^{WA}, is shown on the y axis. The reciprocal frequency of the NA allele is equivalent because $x_i^{NA} = 1 - x_i^{WA}$. Bottom left: enlarged inset of the shaded region showing allele frequency changes in chromosome VIII during selection in rapamycin. Early time points 2, 4, and 8 show localized allele frequency changes at 460–490 kb because of a beneficial NA allele sweeping with hitchhiking passengers. Late time points 16 and 32 show abrupt jumps between successive loci that reflect the parental haplotype of emerging subclone(s). These long-range correlations can alter the frequency of parental alleles independently of their fitness value. In case of a fully clonal population, allele frequencies at 0, 0.5, and 1.0 would correspond to the background genotypes NA/NA, WA/NA, and WA/WA of a diploid clone that reached fixation. Bottom right: we tested a model in which each allele is proposed to be a driver under selection, with linked passenger alleles also changing in frequency by genetic hitchhiking. Top log likelihood scores are shown for all populations in this region of interest (Supplemental Experimental Procedures). We validated the *CTF8*^{NA} allele to be strongly beneficial for rapamycin resistance (Figure S8).
See also Figures S1 and S2.

the frequency of parental alleles under selection (Figure 2). Over time, subclonal populations arose and expanded, depleting the pool of genetic diversity. Here and throughout this article, we employ the term "subclone" to refer to a group of cells that carry the same set of mutations. These successful "macroscopic" subclones could be detected by whole-population sequencing and phenotyping, persisting in time, as manifested by broad jumps in the allele frequency visible across the genome and by multiple modes in the fitness distribution (Figures 2 and 3; Figure S2). But what drives these clonal expansions? Is it the founder haplotypes themselves, *de novo* mutations relegating the parental variation to the role of passengers, or their combined action?

Selective Effects on Pre-existing Genetic Variation

To determine the adaptive value of background variation, we identified regions where local allele frequencies changed over the time course of the selection experiments. Frequency changes over time indicate that selection is acting on beneficial background alleles. These drivers cause linked passenger mutations to also change in frequency by genetic hitchhiking (Illingworth et al., 2012). We performed a systematic scan for background variants under selection using data up to 4 days, when no population yet had detectable subclones that would distort this signal (Supplemental Experimental Procedures). A region of interest was found in chromosome VIII (coordinates 460–490 kb) in all WAxNA populations under rapamycin (Figure 2B). We evaluated two candidate genes in this region by reciprocal hemizygosity, validating the *CTF8*^{NA} allele to increase rapamycin resistance. *CTF8* harbors two background missense variants and has previously been implicated in sensitivity to rapamycin, although the mechanism remains unknown (Parsons et al., 2004). Carrying the *CTF8*^{NA} allele confers a 36% growth rate advantage over the *CTF8*^{WA} allele (Figure S8). *KOG1*, which falls within the same region and is a subunit of the TORC1 complex, differs by seven missense mutations between the parents. However, reciprocal hemizygous deletions only revealed a modest fitness difference between WA and NA sequences of *KOG1*. We did not find events that replicated across all populations in hydroxyurea.

Pervasive Selection of Macroscopic Subclones Driven by *De Novo* Genetic Variation

To reconstruct clonal expansions in the WAxNA populations, we used background genetic variants as markers. Using the cloneHD algorithm (Fischer et al., 2014), we inferred the subclonal genotypes and their frequency in the populations, both of which are unknown *a priori* (Figure S1; Supplemental Experimental Procedures). We found at least one subclone in all WAxNA populations under selection but none in the control environment (Figure 3; Figure S3). Clonal competition was prevalent with two or more expanding subclones in 12 of 16 WAxNA populations.

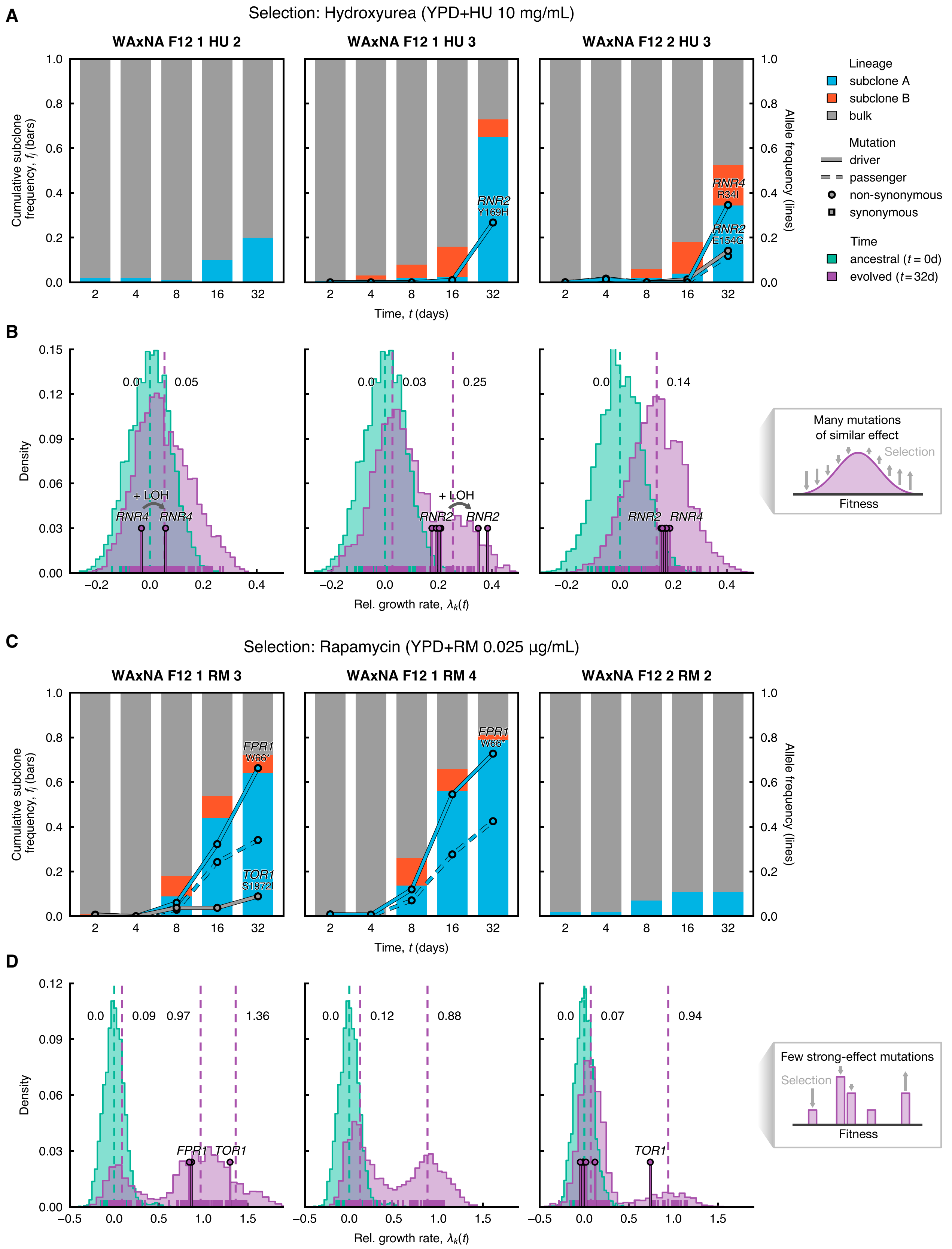

(legend on next page)

No population became fully clonal during the experiment, with subclone frequencies stabilizing after 16 days in several rapamycin populations. Similarly, WA and NA populations under selection underwent adaptation, as evidenced by *de novo* mutation frequencies, except for WA, which became extinct in hydroxyurea (Figure S4).

To genetically characterize the subclones, we isolated and sequenced 44 clones drawn from WAxNA populations after the selection phase (Figure 4; Experimental Procedures). From population and isolate sequence data, we observed 19 recurrent *de novo* mutations in the ribonucleotide reductase subunits *RNR2* and *RNR4* during hydroxyurea selection and in the rapamycin targets *FPR1* and *TOR1* during rapamycin selection (Table 1). Each of these driver mutations had a drug-resistant growth rate phenotype (Figures S6, S7, and S8) and carried a unique background of ~31,000 passenger mutations on average compared with other sequenced isolates. All *FPR1* mutations were homozygous and likely to inactivate the gene or inhibit its expression. In contrast, *TOR1* mutations were heterozygous, whereas we found *RNR2* and *RNR4* mutations in both the heterozygous and homozygous state. All driver mutations occurred in highly conserved functional domains. The variant allele fractions of these mutations mirrored the inferred subclonal dynamics (Figures 3A and 3C; Figures S3 and S4). Other mutated genes with similar dynamics were confirmed as passengers (e.g., *DEP1*, *INP54*, and *YNR066C*; Figure S8). From the genome sequence of the 44 individual clones, we also detected six trisomies as large-scale copy-number aberrations, without conclusive evidence that they are adaptive compared with recurrent point mutations (Figure 4).

Clonal expansions were also evident from changes in the fitness distribution of cells. We established this by phenotyping 96 randomly isolated individuals from 3 populations per environment at 0 and 32 days as well as the 44 sequenced individuals at 32 days (Experimental Procedures). We measured the growth rate of each isolate and determined the population growth rate with respect to the mean of the fitness distribution. The variance of the fitness distribution varied significantly with different drugs, consistent with previous studies (Chevereau et al., 2015). While the variance of the fitness distribution at 0 days was narrow in hydroxyurea ($\sigma^2 = 3.1\times10^{-3}$), growth in rapamycin showed a wider response ($\sigma^2 = 5.4\times10^{-3}$). In rapamycin selection, the fitness distribution became multimodal after 32 days, reflecting the fitness of subclones substantially improving with respect to the mean fitness of the bulk population (Figure 3D). The clonal subpopulation divided on average twice as fast as the ancestral population. Sequenced isolates with driver mutations in *FPR1* and *TOR1* were on the leading edge of the fitness distribution, far ahead of the bulk. Furthermore, the bulk component showed a 10% average improvement, possibly because of selection of beneficial genetic backgrounds. Conversely, bimodality was only detected in one population in hydroxyurea selection (WAxNA F12 1 HU 3), where the clonal peak grew 25% faster on average compared with the ancestral population, and the bulk grew 7% faster on average across all populations (Figure 3B). Isolates with *RNR2* driver mutations fell onto the leading edge of the fitness distribution. These six isolates originated from the same expanding subclone, and two of them had a 13% faster growth rate than the remaining four, although they all shared the same heterozygous *RNR2* driver mutation. In both of these isolates, we found a large region in chromosome II to have undergone loss of heterozygosity (LOH), offering a putative genetic cause for their growth advantage (Figure 4A). Finally, to understand how the fitness of a typical population changes across environments, we characterized the fitness correlations of ancestral and evolved clonal isolates with and without stress (Figure S9). The rank order in clone fitness did not change significantly because of selection when measured in the absence of stress, implying that the evolutionary history of each of the clones did not lead to trade-offs in the average fitness of the population. However, a strong fitness cost of driver mutations in *FPR1* was observed.

Diversification and Genomic Instability

We found several of the driver mutations to exist in homozygous rather than heterozygous states. LOH has been shown to rapidly convert beneficial heterozygous mutations to homozygosity in diploid yeast evolving under nystatin stress (Gerstein et al., 2014). Thus, we hypothesized that genomic instability, causing widespread LOH, could be significantly contributing to adaptation. To detect mechanisms of genomic instability, we used heterozygous genetic variants as markers. First, we used the sequences of haploid individuals from the ancestral population, drawn before the last round of crossing, to create *in silico* diploid genomes and calculate the length distribution of homozygous segments. Similarly, we measured the length distribution of

Figure 3. Reconstruction of Subclonal Dynamics

Competing subclones evolved in hydroxyurea and rapamycin experienced a variety of fates.

(A and C) Time is shown on the x axis, starting after crossing, when the population has no macroscopic subclones and during selection with (A) hydroxyurea and (C) rapamycin between $t = 0$ and $t = 32$ days. Cumulative haplotype frequency of subclones (bars) and allele frequency of *de novo* mutants (lines) are shown on the y axis. Most commonly, selective sweeps were observed where a spontaneous mutation arose and increased in frequency. Driver mutations are shown as solid lines and passenger mutations as dashed lines, colored by subclone assignment; circles and squares denote non-synonymous and synonymous mutations, respectively. For driver mutations, the mutated gene and codon are indicated above each line.

(B and D) Variability in intra-population growth rate, estimated by random sampling of 96 individuals at initial ($t = 0$ days, green) and final time points ($t = 32$ days, purple), before and after selection with (B) hydroxyurea and (D) rapamycin. Relative growth rates $\lambda_k(t)$ by individual k are shown at the foot of the histogram, calculated by averaging over $n_r = 32$ technical replicates per individual. Relative growth rates are normalized with respect to the mean population growth rate $\langle\lambda_k\rangle_{t=0}$ at $t = 0$ days. The posterior means of the distribution modes fitted by a Gaussian mixture model are indicated as dashed lines. The fitter individuals (pins) carry driver mutations, detected by targeted sampling and sequencing. The insets on the right depict a schematic of the fitness distribution in two limit cases: when there are many mutations of similar effect, the fitness wave will be smooth and unimodal; when only few mutations of large effect exist, the fitness distribution will become multimodal.

See also Figures S3, S4, and S10.

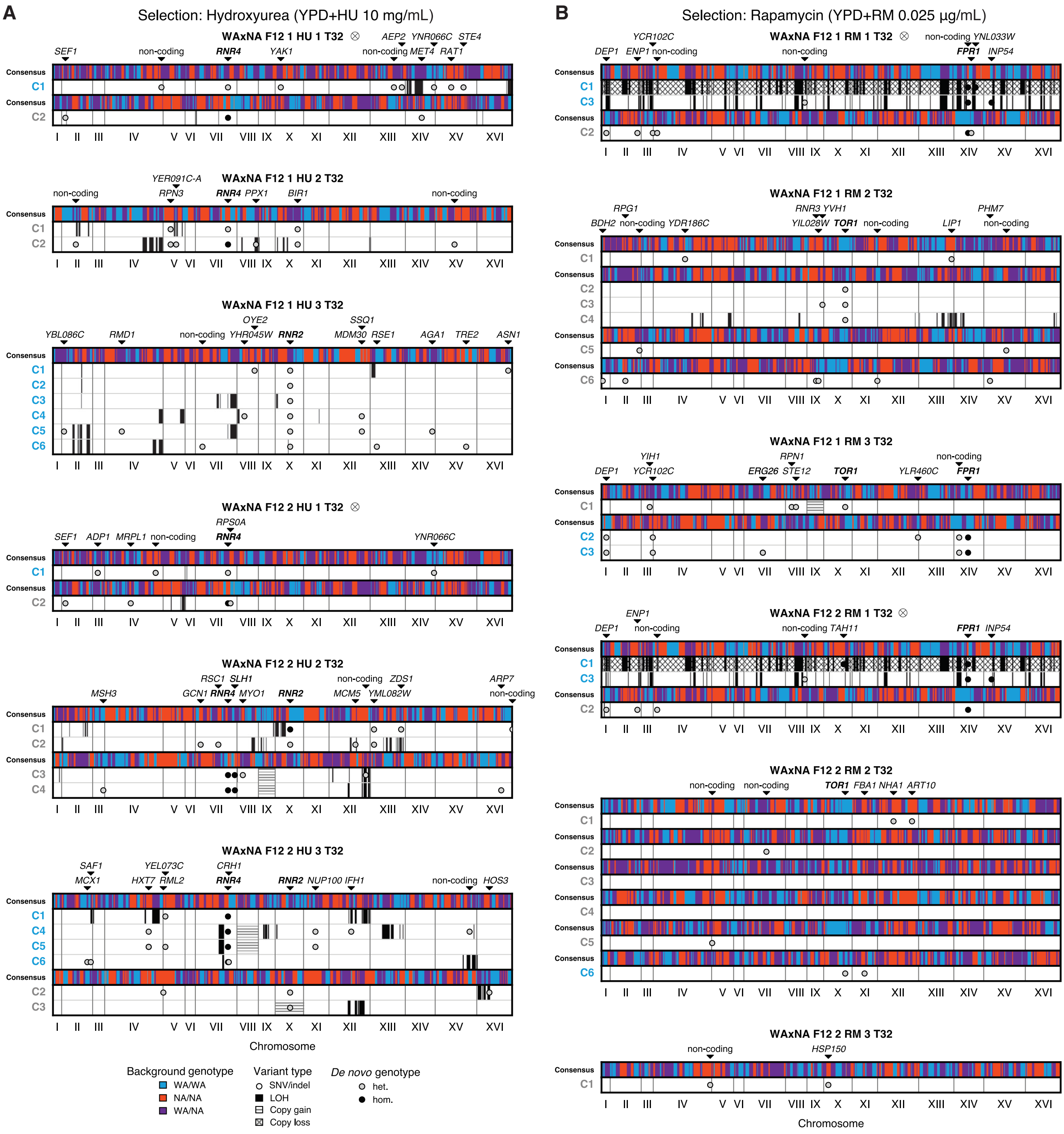

Figure 4. Pervasive Selection for Adaptive Mutations and Genomic Instability

Whole-genome sequences of clones sampled from WAxNA F_{12} populations. SNVs, indels, and chromosome-level aberrations were detected by whole-genome sequencing in single-cell diploid clones derived from evolved populations after $t = 32$ days in (A) hydroxyurea or (B) rapamycin (Table S1). Chromosomes are shown on the x axis; clone isolates are listed on the left, colored by lineage (Figure S3). The consensus shows the majority genotype across population isolates with a sequence identity greater than 80%. WA/WA (blue) and NA/NA (red) represent homozygous diploid genotypes, and WA/NA (purple) represents a heterozygous genotype. Individual cells with a shared background genotype carry *de novo* SNVs and indels (circles), *de novo* mis-segregations with loss of heterozygosity (solid segments), and *de novo* gains or losses in copy number (hatched segments). Driver and passenger mutations are listed along the top (drivers are shown in boldface). Populations marked by ⊗ indicate cross-contamination during the selection phase, but any derived events are independent. All ancestral sequenced isolates can be found in Figure S5. See also Figures 3A and 3C, Table 1, and Table S1.

Table 1. Summary of Driver Mutations

Gene	Chr	Position	Strand	Nucleotide Position	Substitution	Protein Position	Substitution	Status	Genotype	Effect
Selection: Hydroxyurea (YPD+HU 10 mg/mL)										
RNR2	X	392,854	+	451	A > C	151	N > H	*de novo*	N/A	missense
		392,864	+	461	A > G	154	E > G	*de novo*	+/−	missense
		392,908	+	505	T > C	169	Y > H	*de novo*	+/−	missense
		393,020	+	617	C > T	206	T > I	*de novo*	+/−, −/−	missense
RNR4	VII	855,961	−	341	T > A	114	K > M	*de novo*	N/A	missense
		856,201	−	101	C > A	34	R > I	*de novo*	+/−, −/−	missense
		856,202	−	100	T > C	34	R > G	*de novo*	−/−	missense
Selection: Rapamycin (YPD+RM 0.025 μg/mL)										
CTF8	VIII	486,462	−	170	C(WA) > A(NA)	57	G > V	pre-existing	N/A	missense
		486,568	−	64	T(WA) > C(NA)	22	T > A	pre-existing	N/A	missense
FPR1	XIV	371,920	−	306–307	T > TTG	102–103	ST > S*	*de novo*	N/A	frameshift (stop codon gained)
		371,921	−	306	A > T	102	S > R	*de novo*	N/A	missense
		372,030	−	197	C > G	66	W > S	*de novo*	−/−	missense
		372,030	−	197	C > T	66	W > *	*de novo*	−/−	nonsense (stop codon gained)
		372,195	−	28–31	ATTTT > ATTT	10–11	KI > K*	*de novo*	N/A	frameshift (stop codon gained)
		372,224	−	3	C > A	1	M > I	*de novo*	N/A	nonsense (start codon lost)
TOR1	X	564,757	+	5,343–5,345	ATGA > A	1,781–1,782	DD > D	*de novo*	N/A	in-frame deletion
		565,330	+	5,915	G > T	1,972	S > I	*de novo*	+/−	missense
		565,331	+	5,916	C > A	1,972	S > R	*de novo*	N/A	missense
		565,528	+	6,113	G > T	2,038	W > L	*de novo*	+/−	missense
		565,529	+	6,114	G > C	2,038	W > C	*de novo*	+/−	missense
		565,550	+	6,135	C > A	2,045	F > L	*de novo*	N/A	missense

Summary of unique SNVs, insertions and deletions found to be drivers in hydroxyurea (*RNR2* and *RNR4*) and rapamycin (*CTF8*, *FPR1*, and *TOR1*). Nucleotide and protein substitutions show the wild-type and mutated alleles. Nucleotides gained or lost are underlined. Variants are labeled as pre-existing when they differ between the parents and as *de novo* when they arose during the crossing or selection phases of the experiment. The functional effect of the mutations has been characterized using the Ensembl Variant Effect Predictor (McLaren et al., 2016). Populations and clones carrying mutations in these driver genes are listed in Table S1. The genotype of each mutation in individual clones is shown in Figure 4. The genotype of mutations only found by whole-population sequencing cannot be resolved and is indicated as not applicable (N/A). Chr, chromosome.

homozygous segments from evolved isolate genomes. We observed a significant increase of long homozygosity tracts in the evolved clones—a hallmark of LOH (Figure 5A). Second, we directly counted LOH events in populations using multiple sequenced isolates from the same expanding subclone (Supplemental Experimental Procedures).

We identified a minimum of 6 events per genome per subclone (Figure 4). Although this estimate is a lower bound and is limited because of the number of sequenced individuals per subclone, the LOH rates are substantial. To exemplify the interaction of genomic instability with pre-existing and *de novo* variation, inspection of *de novo* mutations in the WAxNA F12 1 HU 3 population shows that one *RNR2* mutation spans six isolates, being part of an expanding subclone (Figure 4A). These isolates have further diversified by acquiring passenger mutations and undergoing LOH. Clones C5 and C6 grow faster than the other four and share a large LOH event in chromosome II that is not present in the other isolates, possibly providing the growth advantage and broadening the fitness distribution (Figure 3B). An alternative route to homozygosity was observed in a single clone found to be haploid (clone C1 in WAxNA F12 2 RM 1) and, therefore, homozygous genome-wide. This haploid clone is closely related to a diploid clone (C3) from the same population, and both clones share the same *FPR1* W66* *de novo* mutation (Figure 4B). These data are consistent with the appearance of the *FPR1* heterozygous mutation in an ancestral diploid clone that took two independent routes—focal LOH or meiosis—to unveil the recessive driver mutation. Altogether, we find that genomic instability can render *de novo* mutations homozygous as a necessary event in a multi-hit process toward drug resistance.

The stress environments themselves have an active role in accelerating genome evolution by genomic instability. Using a fluctuation assay, we investigated the effect of the genetic background and of the selective environment on genomic instability by tracking the loss of the *URA3* marker. Consistent with previous studies (Barbera and Petes, 2006), replication stress

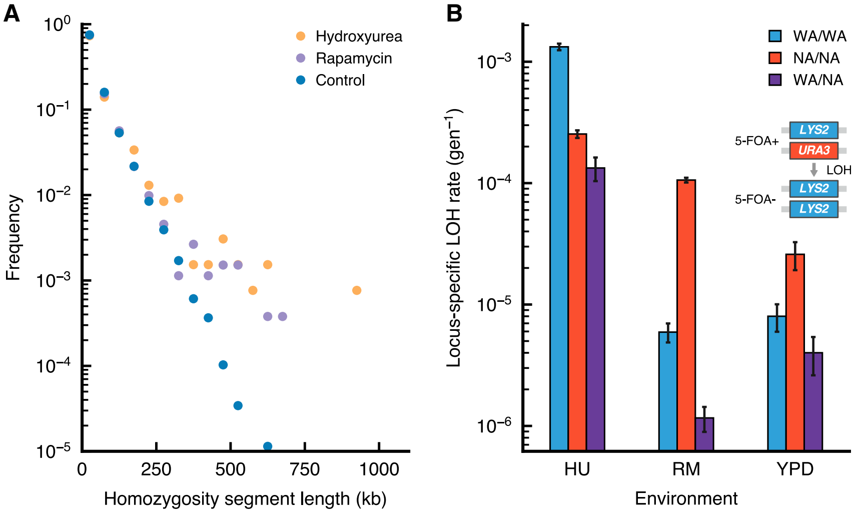

Figure 5. Elevated Rates of Loss of Heterozygosity

(A) The length distribution of homozygous segments, in bins corresponding to 50-kb increments, shows an excess of long homozygosity tracts above 300 kb in hydroxyurea and rapamycin (Kolmogorov-Smirnov test, $p < 0.01$). Ancestral haploid isolates are used to compare a set of *in silico* diploid genomes to evolved diploid isolates. Only unrelated isolate backgrounds were included.

(B) Background- and environment-dependent rates of loss of heterozygosity were measured in a fluctuation assay by loss of the *URA3* marker. Resistant colonies growing in 5-fluororotic acid (5-FOA+) indicate loss of the marker. Based on the number of 5-FOA+ colony-forming units (CFUs), the mean number of LOH events are estimated using the empirical probability-generating function of the Luria-Delbrück distribution (Supplemental Experimental Procedures). The locus-specific LOH rates are shown, given by the mean number of LOH events divided by the total number of cells in YPD. Error bars denote the upper and lower 95% confidence intervals. LOH rates were elevated in hydroxyurea compared with the control environment and manifested background-dependent effects between the parents and their hybrid.

See also Figure 4.

induced by hydroxyurea caused an increase in LOH rates. We also observed a background-dependent increase in LOH in rapamycin (Figure 5B).

Decomposing Fitness Effects of Genetic Variation by Background Averaging

Finally, we sought to partition and quantify the individual fitness contributions of pre-existing and *de novo* genetic variation. The genotype space is extremely vast, but we can uniformly sample a representative ensemble to reconstruct a fraction of the genetic backgrounds where beneficial mutations could have arisen. To this end, we designed a genetic cross where background and *de novo* variants were re-shuffled to create new combinations (Figure 6A). We randomly isolated diploids from both ancestral and evolved populations, sporulated these, and determined whether the derived haploids contained wild-type or mutated *RNR2*, *RNR4*, *FPR1*, and *TOR1* alleles. We then crossed haploids to create a large array of diploid hybrids where all genotypes (+/+, +/–, –/–) for each of these genes existed in an ensemble of backgrounds, thus recreating a large fraction of the genotype space conditioned on the presence or absence of driver mutations. We measured the growth rates of both haploid spores and diploid hybrids, estimating and partitioning the variation in growth rate contributed by the background genotype and by *de novo* genotypes using a linear mixed model (Figure 6B; Figure S10; Supplemental Experimental Procedures).

The ensemble average over backgrounds showed that the mean effect of *RNR2*, *RNR4*, and *TOR1* mutations was fully dominant and highly penetrant regardless of the background (Figures 6D and 6F). In contrast, *FPR1* mutants were recessive and only increased the growth rate when homozygous, again irrespective of the background (Figure 6F). Recombinants with *RNR2* and *RNR4* mutations show epistatic interactions, consistent with the products encoded by these genes, which are known to interact as subunits of the same protein complex (Figure 6C). After conditioning for *RNR2*, *RNR4*, *FPR1*, and *TOR1* driver mutation status, a large fraction of the phenotypic variance still remained, reflecting the effect of the genetic backgrounds in which they emerged (Figures 6C and 6E). In fact, under hydroxyurea exposure, background genetic variation accounted for an estimated 51% of the growth rate variance, more than twice the estimated 23% contributed by *RNR2* and *RNR4 de novo* mutations. Furthermore, these mutations eventually landed on genetic backgrounds much fitter than average in the ancestral fitness distribution, as denoted by the estimated 7% explained by the time of sampling. Both of these results directly imply that moderate-effect *de novo* mutations must arise on favorable genetic backgrounds to give rise to macroscopic subclones. In contrast, under rapamycin exposure, the pre-existing genetic variation accounted for only 22% of the variance, much less than the 70% attributed to *FPR1* and *TOR1* mutations. Such large-effect mutations can expand in a vast majority of backgrounds, explaining how they can almost entirely surpass the bulk of the fitness distribution (Figure 3D). Taken together, these results are consistent with the aggregation of small-effect, pre-existing variants that can condition the fate of new mutations in both selection environments.

DISCUSSION

Here we showed that populations containing extensive fitness variability can adapt to strong selective pressures utilizing both pre-existing and *de novo* genetic variation. Theory predicts that pre-existing genetic variation forms a traveling fitness wave, with the mean fitness increasing at a rate that is proportional to its fitness variance (Desai and Fisher, 2007; Rouzine and Coffin, 2005). New mutations are expected to be successful when they land on a favorable background or when they are beneficial enough to escape from the bulk dynamics by their own merits. Recent theoretical results have suggested the

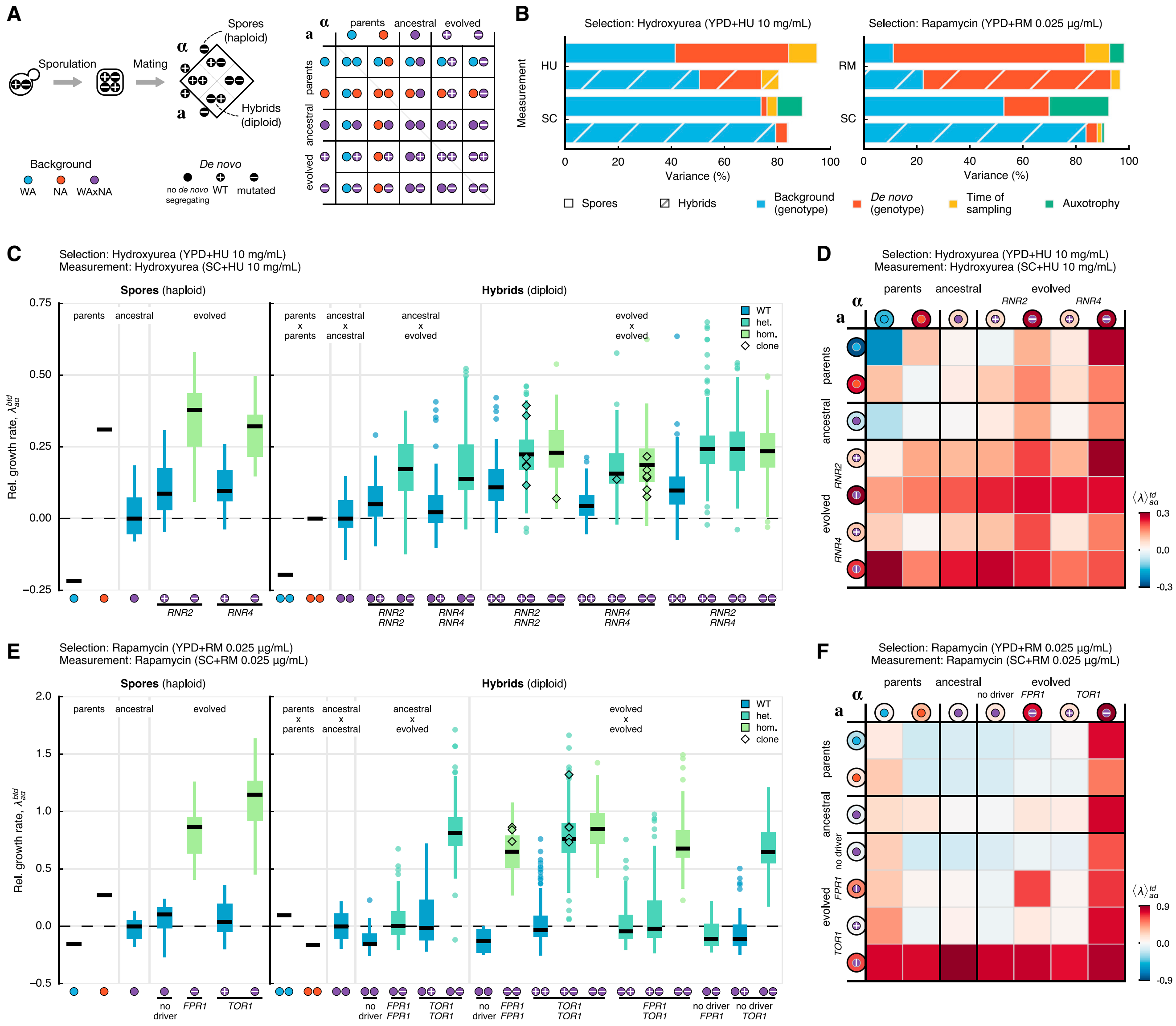

Figure 6. Ensemble-Averaged Fitness Effects of Genetic Background and *De Novo* Mutations

(A) To quantify the fitness effects of background variation and *de novo* mutations in hydroxyurea (*RNR2* and *RNR4*) and rapamycin (*FPR1* and *TOR1*), we isolated individuals from ancestral and evolved populations. From these diploid cells, we sporulated and selected haploid segregants of each mating type. Spores with mutations in *RNR2*, *RNR4*, and *TOR1* were genotyped to test whether they carry the wild-type or mutated allele. We crossed the *MAT*a and *MAT*α versions to create hybrids (48 × 48 in hydroxyurea and 56 × 56 in rapamycin). Independent segregants were used to measure the biological variability of ancestral and evolved backgrounds.

(B) Variance decomposition of the growth rate of spores (solid) and hybrids (hatched) that can be attributed to different components using a linear mixed model. The model components are the background genotype, *b*; *de novo* genotype, *d*; time of sampling during the selection phase, *t*; and auxotrophy, *x*. Estimates of variance components are obtained by restricted maximum likelihood (Figure S12 and Table S6).

(C and E) Relative growth rate of spores, $\lambda^{btd}_{\{a,\alpha\}}$, and hybrids, $\lambda^{btd}_{a\alpha}$, measured for multiple combinations of background and *de novo* genotypes and averaged over measurement replicates. Relative growth rates are normalized with respect to the mean growth rate of the ancestral WAxNA cross. Measurements of cells selected in (C) hydroxyurea and (E) rapamycin were taken in the respective stress environments. Medians and 25%/75% percentiles across groups are shown, with medians shown as horizontal black lines and colored by *de novo* genotype (wild-type, blue; heterozygote, cyan; homozygote, green). Outliers (circles) and isolated, selected clones with matching genotypes (diamonds) are highlighted.

(D and F) Ensemble average of the relative growth rate of spores, $\langle\lambda\rangle^{td}_{\{a,\alpha\}}$, and hybrids, $\langle\lambda\rangle^{td}_{a\alpha}$, measured in (D) hydroxyurea and (F) rapamycin. The color scale for all matrices is shown at the right and indicates the difference in the ensemble average with respect to the ancestral WAxNA crosses.

The symbols in (C)–(F) follow the legend in (A) and indicate combinations of the type of genetic background (WA parent, ●; NA parent, ●; WAxNA segregant, ●) and the genotype of *de novo* mutations (no *de novo* mutation, ●; wild-type, ⊕; mutated, ⊖). An extended version of the figure with all combinations and controls can be found in Figures S10 and S11, respectively.

existence of a selective advantage threshold above which the fate of a new mutation becomes decoupled from the background on which it lands (Good et al., 2012; Schiffels et al., 2011). Our results show that new beneficial mutations expanded on a range of genetic backgrounds and selection concomitantly acted on pre-existing variation through its combined effects on fitness, steadily improving the bulk of the population. The observed dynamics are, at this level, consistent with the theoretical picture.

The rate of adaptation and the type of beneficial mutations that expand and fix in a population depend on multiple factors, such as population size, mutation rate, and ploidy (Barrick and Lenski, 2013; Selmecki et al., 2015; zur Wiesch et al., 2011). Our results show that sufficiently large populations could readily find beneficial *de novo* mutations, but their adaptive trajectories were simultaneously shaped by pre-existing and *de novo* variation with overlapping timescales. Previous experimental studies with substantial founder diversity did not observe *de novo* mutations playing an important role in either asexual or sexual evolution (Burke et al., 2010, 2014; Parts et al., 2011). This may be due to differences in the selective constraints that affect the timescale for the emergence of *de novo* mutations or may depend on the genetic architecture of the selected phenotype, making the background fitness variation harder to overcome. Despite the large genetic heterogeneity of the founders, mutations in driver genes were recurrent, indicating convergent evolution toward a restricted number of molecular targets. This is an important aspect to be able to predict the outcome of selection. Larger studies that systematically vary key parameters, such as population size, are needed to quantify how pre-existing variation conditions the repeatability of new mutations.

Measurements of the fitness distribution revealed markedly different variability within a population in response to different inhibitors. There were two different outcomes of selection: when many mutations had comparable fitness effects as in hydroxyurea, the fitness distribution remained smooth; on the contrary, when few large-effect mutations were available, such as mutations in the target-of-rapamycin (TOR) pathway in rapamycin, the fitness distribution became multimodal. We were not able to attribute increases in the bulk of the fitness distribution to particular alleles beyond the *CTF8* gene, probably because of the contribution of many small-effect loci. Previous studies in isogenic populations have reported adaptive mutations sweeping to fixation on a comparable timescale without specific selective constraints such as drugs (Lang et al., 2013). In contrast, we did not observe complete fixations. This is partially due to the duration of the experiment: the clones are still expanding after 32 days in hydroxyurea. However, most rapamycin-resistant clones become stable between 16 and 32 days. Although we do not know the underlying cause, the observation has important consequences. Notably, the substantial genotypic and phenotypic diversity that remained after selection could be a potent substrate to re-sensitize a population and may compromise targeted therapies against resistant clones. Understanding the role of clonal competition in isogenic and heterogeneous populations requires further work, which could be approached experimentally using lineage tracing (Levy et al., 2015).

We observed a balance between the loss of diversity because of selection and active diversification mechanisms that partially re-established and refined existing variants. The background not only contributed substantially to fitness but was also continuously re-configured by genomic instability, diversifying the expanding clones. Chromosomal rearrangements represent a key mechanism in shaping genome diversity in asexual organisms (Dunham et al., 2002; Flot et al., 2013) and in somatic evolution of cancer (Stephens et al., 2011), where cells accumulate a genetic load during tumor development that LOH can reveal phenotypically. In asexual diploids such as those studied here, successful beneficial mutations are expected to be dominant in a phenomenon known as Haldane's sieve (Orr and Betancourt, 2001). However, LOH has been shown to overcome this constraint by rapidly converting initially heterozygous mutations to homozygosity (Gerstein et al., 2014). Therefore, LOH may enable asexually evolving populations to approach the adaptive rates seen in sexual organisms with recombination. Here we also saw these dynamics at play because recessive *FPR1* mutations needed a second hit by LOH. Additionally, the process gained a new dimension: although these rearrangements were mostly copy number-neutral, they led to fitness increments by changing scores of background variation from the heterozygous to the homozygous state in a single step. As a result, certain passenger mutations hitchhiking with a beneficial driver may provide an additional fitness advantage distributed across one or multiple loci (Figure 4). The implications of the ongoing diversification by chromosomal rearrangements are worthwhile pursuing further, both theoretically and experimentally. Even if a driver mutation were to fully fix, a substantial amount of genetic variation would remain. Multiple genetic backgrounds with the same driver mutation would diverge (Hermisson and Pennings, 2005), and it may drastically alter the theoretical expectation of a sharp transition between evolutionary regimes at the selective threshold (Good et al., 2012; Schiffels et al., 2011). Experimentally, recently developed genome-editing techniques may enable localizing and measuring the fitness effect of specific LOH regions (Sadhu et al., 2016).

We carried out background-averaged fitness measurements of a recombinant library of pre-existing and *de novo* mutations. We found that large-effect mutations, such as those in the TOR pathway, confer resistance to rapamycin regardless of the genetic background where they arise. These mutations were of sufficient magnitude to surpass the bulk of the fitness distribution and can be interpreted to be above the selective threshold. Conversely, the pre-existing fitness variance influenced the fate of *de novo* drivers like *RNR2* and *RNR4* mutations, which needed to land on a favorable background to be competitive. Thus far, most biological systems have been found at the edge of the two regimes. Large-effect mutations being amplified on well-adapted background genotypes have been observed in laboratory populations (Lang et al., 2011) and in the wild (e.g., in the seasonal influenza virus; Illingworth and Mustonen, 2012; Luksza and Lässig, 2014), which suggests that these dynamics represent a general mode of adaptation. Interestingly, our combinatorial strategy of background averaging shows that both of the limit cases can be true. Thus, the predictability of the outcomes of selection will hinge on characterizing the background fitness variance and finding a common framework to describe the selective potential of a population (Boyer et al.,

2016). Detecting a known driver mutation without a measurement of the background fitness distribution will be insufficient to predict its ultimate fate. This is a necessary requisite to eventually rationalize the design of therapies for the treatment of bacterial and viral infections or cancer. It may also be possible to balance and control the fitness effects of pre-existent and *de novo* mutations—i.e., to change the selective threshold; for example, by modulating the dose-dependent effects of inhibitors (Cheverreau et al., 2015) or by inhibiting global regulators (Jarosz and Lindquist, 2010).

Taken together, our findings can help us understand the evolution of large asexual populations with extensive genetic variation. Bacterial infections and cancer, which easily reach sizes of billions of cells, host a comparable mutation load before any selective treatment is applied. For example, the number of pre-existing variants in our experiment is comparable with the typical number of somatic mutations accrued before treatment during carcinogenesis, which varies between 10^2–10^5 depending on the cancer type (Lawrence et al., 2013), and it is also comparable with the genetic diversity in bacterial communities (e.g., in cystic fibrosis patients; Lieberman et al., 2014). In either of these cases, the number of possible mutations available to escape antimicrobial or chemotherapy drugs is limited, and it is comparable with the balance we observe between the number of drivers and passengers. Clearly, whether these results hold true more generally needs to be studied across systems. Overall, we hope that our results will encourage new theoretical and empirical investigations of the complex interplay of selection simultaneously acting on pre-existing and *de novo* genetic variation and of the role of genomic instability continuously molding the genomes in a population.

EXPERIMENTAL PROCEDURES

A summary of the experimental protocols of this study is presented here. A full exposé of the experimental methods is given in the Supplemental Experimental Procedures, where we describe protocols for clone isolation, engineering genetic constructs, genetic crossing, fluctuation assays, and growth phenotyping. This is followed by a presentation of the theory and data analysis, where we define the model for localization of drivers among hitchhiking passengers and the probabilistic inference method for subclonal reconstruction. Furthermore, we also discuss the model for the estimation of variance components from background-averaged fitness measurements.

Study Design

In our study, we begin with two yeast strains that have diverged over millions of generations (divergence phase) and are randomly mated by meiotic recombination to generate a large pool of recombinant mosaic haplotypes (crossing phase), followed by applying a selective constraint of the population under stress (selection phase).

Divergence Phase

Parental strains were derived from a WA strain (DBVPG6044; *MATα*, *ura3::KanMX*, *lys2::URA3*, *ho::HphMX*) isolated from palm wine and a NA strain (YPS128; *MAT*a, *ura3::KanMX*, *ho::HphMX*) isolated from the oak tree. These strains were selected from two diverged *S. cerevisiae* lineages and feature 52,466 single-nucleotide differences uniformly distributed across the genome.

Crossing Phase

The selection experiments were carried out using WA, NA, WAxNA F_2, and WAxNA F_{12} founder populations derived from hybrids between WA and NA. The WAxNA F_2 and F_{12} populations were, respectively, generated from the F_1 and F_{11} hybrids between WA and NA. The WAxNA F_1/F_{11} diploid populations were expanded in YPD and sporulated in solid potassium acetate (KAc) medium (2% potassium acetate, 2% agar) for 14 days at 23°C. Sporulation of diploids was confirmed by visual inspection of asci. Over 90% of sporulation efficiency was observed after 14 days. Any remaining unsporulated cells were selectively removed using the ether protocol (Parts et al., 2011). The haploid population was subjected to mass mating according to the protocol described by Parts et al. (2011). Briefly, the asci were resuspended in 900 μL of sterile water and digested with 100 μL of zymolase (10 mg mL^{-1}) for 1 hr at 37°C. The cells were washed twice with 800 μL of sterile water, vortexed for 5 min to allow spore dispersion, plated in YPD, and incubated for 2 days at 23°C. The YPD plates were replica-plated in minimal medium to select diploid cells (*MAT*a/*MATα*, *LYS2*/*lys2::URA3*). The WAxNA F_2/F_{12} generation was collected from the plates and used as a founder population for the selection experiments and stored at −80°C as a frozen stock.

Selection Phase

In the selection phase, WA, NA, WAxNA F_2, and WAxNA F_{12} founder populations (referred to as ancestral) were evolved asexually in two selective environments and one control environment. Each of the ancestral populations consisted of a total population size of 3.2×10^7 cells, determined by plating and counting colony-forming units. We serially propagated multiple replicate populations over a period of 32 days, which we refer to as evolved populations. Every 48 hr, 1:10 of the total cell population was transferred to fresh plates, avoiding severe bottlenecks to minimize the effect of genetic drift. We estimated that 1.74 generations per day took place in hydroxyurea and 1.63 generations per day in rapamycin, based on the mean growth rate of three representative populations in each environment and accounting for acceleration and deceleration of growth every 48 hr cycle (Supplemental Experimental Procedures). These empirical estimates amount to ∼54 generations between 0 and 32 days, in agreement with a theoretical bound on the number of generations, assuming exponential growth with a 1:10 dilution factor every 48 hr.

Where indicated, the selective media were supplemented with hydroxyurea (HU) at 10 mg mL^{-1} or rapamycin (RM) at 0.025 μg mL^{-1} and maintained at constant drug concentration until day 34. The drug concentrations were chosen based on the dose response of the WA and NA strains. We selected concentrations that maximized the differential growth between the two diploid parents in each environment. We observed a clear dose response in hydroxyurea, with at least 10-fold differential growth between the two diploid parent strains at 10 mg mL^{-1} (Figure S7). For rapamycin, we used 0.025 μg mL^{-1}, which also results in a 10-fold difference between the parent strains (Figure S8). This concentration is well below the minimum inhibitory concentration of 0.1 μg mL^{-1} originally used to identify the highly penetrant *TOR1* mutations in the lab strain (Heitman et al., 1991).

Whole-Genome Sequencing and Phenotyping

We followed the evolution of these populations over the course of the experiment using whole-genome sequencing and phenotyping of the bulk population and of ancestral and evolved isolates. WA and NA populations are labeled by their background, the environment in the selection phase, and the selection replicate; e.g., NA RM 1. WAxNA populations are labeled by background, number of crossing rounds, cross replicate, selection environment, and selection replicate; e.g., WAxNA F12 2 HU 1. Time series samples are labeled from T0 to T32, and isolate clones carry a suffix; e.g., C1, C2, etc. Whole-population sequencing was performed after t = 0, 2, 4, 8, 16, and 32 days, and ancestral and evolved individuals were also sequenced (Table S1). Genomic DNA was extracted from the samples using the Yeast MasterPure kit (Epicenter, USA). The samples were sequenced with Illumina TruSeq SBS v4 chemistry using paired-end sequencing on Illumina HiSeq 2000/2500 at the Wellcome Trust Sanger Institute. Phenotyping of ancestral and evolved individuals was performed by monitoring growth after t = 0 and 32 days using transmissive scanning (Supplemental Experimental Procedures).

DATA AND SOFTWARE AVAILABILITY

The study accessions for the sequence data reported in this paper are available from the European Nucleotide Archive (ENA) and the NCBI BioProject.

The dataset in study accession PRJEB2608 corresponds to raw DNA sequence reads previously reported in Parts et al. (2011). The dataset in study accession PRJEB4645 corresponds to raw DNA sequence reads newly reported in this study. The dataset in study accession PRJEB13491 corresponds to mutation calls in the two aforementioned datasets. All datasets have been jointly analyzed in this manuscript.

Phenotype data, fluctuation assay data, code, and notebooks are available from the GitHub repository (https://github.com/ivazquez/clonal-heterogeneity)

SUPPLEMENTAL INFORMATION

Supplemental Information includes Supplemental Experimental Procedures, twelve figures, and six tables and can be found with this article online at https://doi.org/10.1016/j.celrep.2017.09.046.

AUTHOR CONTRIBUTIONS

I.V.-G., J.W., V.M., and G.L. designed the research. F.S., J.L., B.B., J.H., A.B., and E.A.P. conducted the experiments. I.V.-G., A.F., and V.M. developed the theory, implemented computational methods, and analyzed data. I.V.-G., V.M., and G.L. wrote the paper.

ACKNOWLEDGMENTS

We thank Agnès Llored, Jordi Tronchoni, and Martin Zackrisson for technical help; Elizabeth Gibson for support with library preparation and sequencing; and Erik Garrison, Daniel Kunz, Leopold Parts, David Posada, and Magda Reis for critical reading of the manuscript. We also thank participants of the program on Evolution of Drug Resistance held at the Kavli Institute for Theoretical Physics (University of California, Santa Barbara) for discussions. I.V.-G. is a recipient of a Wellcome Trust Ph.D. fellowship and a Sanger Early Career Innovation Award. This research was supported by Wellcome Trust grants WT097678 (to I.V.-G.) and WT098051 (to V.M.), Fundación Ibercaja (to I.V.-G.), ATIP-Avenir (CNRS/INSERM), Fondation ARC grant SFI20111203947, FP7-PEOPLE-2012-CIG grant 322035, French National Research Agency grant ANR-13-BSV6-0006-01, Cancéropôle PACA (AAP Emergence), and a DuPont Young Professor Award (to G.L.). F.S. was supported by ATIP-Avenir (CNRS/INSERM), Becas Chile grant 74130015, CONICYT/FONDECYT grant 3150156, and MN-FISB grant NC120043 postdoctoral fellowships. A.F. was supported by German Research Foundation grant FI 1882/1-1, J.L. by Fondation ARC grant PDF20140601375, B.B. by La Ligue Contre le Cancer grant GB-MA-CD-11287, and J.H. by French National Research Agency grant 11-LABX-0028-01.

Received: March 25, 2017
Revised: May 25, 2017
Accepted: September 14, 2017
Published: October 17, 2017

REFERENCES

Balaban, N.Q., Merrin, J., Chait, R., Kowalik, L., and Leibler, S. (2004). Bacterial persistence as a phenotypic switch. Science *305*, 1622–1625.

Barbera, M.A., and Petes, T.D. (2006). Selection and analysis of spontaneous reciprocal mitotic cross-overs in Saccharomyces cerevisiae. Proc. Natl. Acad. Sci. USA *103*, 12819–12824.

Barrick, J.E., and Lenski, R.E. (2013). Genome dynamics during experimental evolution. Nat. Rev. Genet. *14*, 827–839.

Boyer, S., Biswas, D., Kumar Soshee, A., Scaramozzino, N., Nizak, C., and Rivoire, O. (2016). Hierarchy and extremes in selections from pools of randomized proteins. Proc. Natl. Acad. Sci. USA *113*, 3482–3487.

Burke, M.K., Dunham, J.P., Shahrestani, P., Thornton, K.R., Rose, M.R., and Long, A.D. (2010). Genome-wide analysis of a long-term evolution experiment with *Drosophila*. Nature *467*, 587–590.

Burke, M.K., Liti, G., and Long, A.D. (2014). Standing genetic variation drives repeatable experimental evolution in outcrossing populations of Saccharomyces cerevisiae. Mol. Biol. Evol. *31*, 3228–3239.

Chevereau, G., Dravecká, M., Batur, T., Guvenek, A., Ayhan, D.H., Toprak, E., and Bollenbach, T. (2015). Quantifying the determinants of evolutionary dynamics leading to drug resistance. PLoS Biol. *13*, e1002299.

Desai, M.M., and Fisher, D.S. (2007). Beneficial mutation selection balance and the effect of linkage on positive selection. Genetics *176*, 1759–1798.

Dunham, M.J., Badrane, H., Ferea, T., Adams, J., Brown, P.O., Rosenzweig, F., and Botstein, D. (2002). Characteristic genome rearrangements in experimental evolution of Saccharomyces cerevisiae. Proc. Natl. Acad. Sci. USA *99*, 16144–16149.

Fischer, A., Vázquez-García, I., Illingworth, C.J.R., and Mustonen, V. (2014). High-definition reconstruction of clonal composition in cancer. Cell Rep. *7*, 1740–1752.

Flot, J.-F., Hespeels, B., Li, X., Noel, B., Arkhipova, I., Danchin, E.G.J., Hejnol, A., Henrissat, B., Koszul, R., Aury, J.-M., et al. (2013). Genomic evidence for ameiotic evolution in the bdelloid rotifer Adineta vaga. Nature *500*, 453–457.

Gerlinger, M., Rowan, A.J., Horswell, S., Math, M., Larkin, J., Endesfelder, D., Gronroos, E., Martinez, P., Matthews, N., Stewart, A., et al. (2012). Intratumor heterogeneity and branched evolution revealed by multiregion sequencing. N. Engl. J. Med. *366*, 883–892.

Gerrish, P.J., and Lenski, R.E. (1998). The fate of competing beneficial mutations in an asexual population. Genetica *102-103*, 127–144.

Gerstein, A.C., Kuzmin, A., and Otto, S.P. (2014). Loss-of-heterozygosity facilitates passage through Haldane's sieve for Saccharomyces cerevisiae undergoing adaptation. Nat. Commun. *5*, 3819.

Good, B.H., Rouzine, I.M., Balick, D.J., Hallatschek, O., and Desai, M.M. (2012). Distribution of fixed beneficial mutations and the rate of adaptation in asexual populations. Proc. Natl. Acad. Sci. USA *109*, 4950–4955.

Heitman, J., Movva, N.R., and Hall, M.N. (1991). Targets for cell cycle arrest by the immunosuppressant rapamycin in yeast. Science *253*, 905–909.

Hermisson, J., and Pennings, P.S. (2005). Soft sweeps: molecular population genetics of adaptation from standing genetic variation. Genetics *169*, 2335–2352.

Illingworth, C.J.R., and Mustonen, V. (2012). Components of selection in the evolution of the influenza virus: linkage effects beat inherent selection. PLoS Pathog. *8*, e1003091.

Illingworth, C.J.R., Parts, L., Schiffels, S., Liti, G., and Mustonen, V. (2012). Quantifying selection acting on a complex trait using allele frequency time series data. Mol. Biol. Evol. *29*, 1187–1197.

Jarosz, D.F., and Lindquist, S. (2010). Hsp90 and environmental stress transform the adaptive value of natural genetic variation. Science *330*, 1820–1824.

Landau, D.A., Carter, S.L., Stojanov, P., McKenna, A., Stevenson, K., Lawrence, M.S., Sougnez, C., Stewart, C., Sivachenko, A., Wang, L., et al. (2013). Evolution and impact of subclonal mutations in chronic lymphocytic leukemia. Cell *152*, 714–726.

Lang, G.I., Botstein, D., and Desai, M.M. (2011). Genetic variation and the fate of beneficial mutations in asexual populations. Genetics *188*, 647–661.

Lang, G.I., Rice, D.P., Hickman, M.J., Sodergren, E., Weinstock, G.M., Botstein, D., and Desai, M.M. (2013). Pervasive genetic hitchhiking and clonal interference in forty evolving yeast populations. Nature *500*, 571–574.

Lawrence, M.S., Stojanov, P., Polak, P., Kryukov, G.V., Cibulskis, K., Sivachenko, A., Carter, S.L., Stewart, C., Mermel, C.H., Roberts, S.A., et al. (2013). Mutational heterogeneity in cancer and the search for new cancer-associated genes. Nature *499*, 214–218.

Levy, S.F., Blundell, J.R., Venkataram, S., Petrov, D.A., Fisher, D.S., and Sherlock, G. (2015). Quantitative evolutionary dynamics using high-resolution lineage tracking. Nature *519*, 181–186.

Lieberman, T.D., Flett, K.B., Yelin, I., Martin, T.R., McAdam, A.J., Priebe, G.P., and Kishony, R. (2014). Genetic variation of a bacterial pathogen within

individuals with cystic fibrosis provides a record of selective pressures. Nat. Genet. *46*, 82–87.

Luksza, M., and Lässig, M. (2014). A predictive fitness model for influenza. Nature *507*, 57–61.

Luria, S.E., and Delbrück, M. (1943). Mutations of bacteria from virus sensitivity to virus resistance. Genetics *28*, 491–511.

Marusyk, A., Tabassum, D.P., Altrock, P.M., Almendro, V., Michor, F., and Polyak, K. (2014). Non-cell-autonomous driving of tumour growth supports subclonal heterogeneity. Nature *514*, 54–58.

McLaren, W., Gil, L., Hunt, S.E., Riat, H.S., Ritchie, G.R.S., Thormann, A., Flicek, P., and Cunningham, F. (2016). The Ensembl Variant Effect Predictor. Genome Biol. *17*, 122.

Neher, R.A. (2013). Genetic draft, selective interference, and population genetics of rapid adaptation. Annu. Rev. Ecol. Evol. Syst. *44*, 195–215.

Orr, H.A., and Betancourt, A.J. (2001). Haldane's sieve and adaptation from the standing genetic variation. Genetics *157*, 875–884.

Parsons, A.B., Brost, R.L., Ding, H., Li, Z., Zhang, C., Sheikh, B., Brown, G.W., Kane, P.M., Hughes, T.R., and Boone, C. (2004). Integration of chemical-genetic and genetic interaction data links bioactive compounds to cellular target pathways. Nat. Biotechnol. *22*, 62–69.

Parts, L., Cubillos, F.A., Warringer, J., Jain, K., Salinas, F., Bumpstead, S.J., Molin, M., Zia, A., Simpson, J.T., Quail, M.A., et al. (2011). Revealing the genetic structure of a trait by sequencing a population under selection. Genome Res. *21*, 1131–1138.

Peter, B.M., Huerta-Sanchez, E., and Nielsen, R. (2012). Distinguishing between selective sweeps from standing variation and from a de novo mutation. PLoS Genet. *8*, e1003011.

Rouzine, I.M., and Coffin, J.M. (2005). Evolution of human immunodeficiency virus under selection and weak recombination. Genetics *170*, 7–18.

Sadhu, M.J., Bloom, J.S., Day, L., and Kruglyak, L. (2016). CRISPR-directed mitotic recombination enables genetic mapping without crosses. Science *352*, 1113–1116.

Schiffels, S., Szöllosi, G.J., Mustonen, V., and Lässig, M. (2011). Emergent neutrality in adaptive asexual evolution. Genetics *189*, 1361–1375.

Selmecki, A.M., Maruvka, Y.E., Richmond, P.A., Guillet, M., Shoresh, N., Sorenson, A.L., De, S., Kishony, R., Michor, F., Dowell, R., and Pellman, D. (2015). Polyploidy can drive rapid adaptation in yeast. Nature *519*, 349–352.

Stephens, P.J., Greenman, C.D., Fu, B., Yang, F., Bignell, G.R., Mudie, L.J., Pleasance, E.D., Lau, K.W., Beare, D., Stebbings, L.A., et al. (2011). Massive genomic rearrangement acquired in a single catastrophic event during cancer development. Cell *144*, 27–40.

Toprak, E., Veres, A., Michel, J.-B., Chait, R., Hartl, D.L., and Kishony, R. (2011). Evolutionary paths to antibiotic resistance under dynamically sustained drug selection. Nat. Genet. *44*, 101–105.

Venkataram, S., Dunn, B., Li, Y., Agarwala, A., Chang, J., Ebel, E.R., Geiler-Samerotte, K., Hérissant, L., Blundell, J.R., Levy, S.F., et al. (2016). Development of a comprehensive genotype-to-fitness map of adaptation-driving mutations in yeast. Cell *166*, 1585–1596.e22.

Zhu, Y.O., Siegal, M.L., Hall, D.W., and Petrov, D.A. (2014). Precise estimates of mutation rate and spectrum in yeast. Proc. Natl. Acad. Sci. USA *111*, E2310–E2318.

zur Wiesch, P.A., Kouyos, R., Engelstädter, J., Regoes, R.R., and Bonhoeffer, S. (2011). Population biological principles of drug-resistance evolution in infectious diseases. Lancet Infect. Dis. *11*, 236–247.

Cell Reports, Volume *21*

Supplemental Information

Clonal Heterogeneity Influences the Fate of New Adaptive Mutations

Ignacio Vázquez-García, Francisco Salinas, Jing Li, Andrej Fischer, Benjamin Barré, Johan Hallin, Anders Bergström, Elisa Alonso-Perez, Jonas Warringer, Ville Mustonen, and Gianni Liti

Supplemental Information

Supplemental Experimental Procedures

1 Experimental methods . . . .
  1.1 Random and targeted clone isolation . . . .
  1.2 Engineered genetic constructs . . . .
  1.3 Genetic cross . . . .
  1.4 Luria-Delbrück fluctuation assay . . . .
  1.5 Growth phenotyping . . . .
  1.6 Media composition . . . .
2 Theory and data analysis . . . .
  2.1 Sequence analysis . . . .
  2.2 Genome-wide scan of pre-existing variants under selection . . . .
  2.3 Reconstruction of subclonal composition . . . .
  2.4 Subclonal dynamics resolved by whole-population genome sequencing . .
  2.5 Adaptive *de novo* mutations and genomic instability in clonal isolates . . .
  2.6 Validation of putative driver genes . . . .
  2.7 Fitness distribution and population averaging . . . .
  2.8 Decomposition of background-averaged fitness effects of mutations . . . .

Supplemental Figures

Supplemental Tables

Supplemental References

Supplemental Experimental Procedures

1 Experimental methods

1.1 Random and targeted clone isolation

We isolated ancestral and evolved individuals from representative selection experiments to characterize their individual genome sequences and their fitness. Each sample underwent serial dilution to attain a single-cell bottleneck. We isolated individuals from both ancestral populations (WAxNA F12 1 YPD, WAxNA F12 2 YPD) and 6 evolved populations (WAxNA F12 1 HU 2, WAxNA F12 1 HU 3, WAxNA F12 2 HU 3, WAxNA F12 1 RM 3, WAxNA F12 1 RM 4, WAxNA F12 2 RM 2) to measure the initial and final fitness distribution. 96 colonies were randomly picked from each population to span a range of fitness. We measured their growth rate using the high-resolution scanning platform described in 'Growth phenotyping'.

Furthermore, we isolated individuals at the fitter end of the fitness distribution, possibly harboring driver mutations. Since adaptation to one environment typically results in fitness gains or losses in other environments, we profiled 96 individuals from each selection experiment with an array of 6 different environments (YPD, HU at $10\,\mathrm{mg\,mL^{-1}}$, RM at $0.025\,\mathrm{g\,mL^{-1}}$, galactose at 2%, heat at 40 °C and sodium arsenite at 1.5 mM) to discriminate cells based on their phenotypic response. After visual inspection of shared effects across environments, we tested genetic markers by PCR, digestion and targeted resequencing of *de novo* mutations identified from the genome analysis of whole populations. In the hydroxyurea experiment, a heterozygous mutation in *RNR4* was genotyped by PCR followed by *BanI* digestion. In the rapamycin experiment, heterozygous *DEP1* and *INP54 de novo* mutations were genotyped using PCR, followed by *AluI* digestion and confirmed by Sanger sequencing in a subset of samples. We chose a total of 44 clones (22 per environment) for whole-genome sequencing (Table S1).

1.2 Engineered genetic constructs

We selected two genes in which we found putative driver mutations in hydroxyurea (*RNR2*, *RNR4*) and five genes in rapamycin (*CTF8*, *DEP1*, *FPR1*, *TOR1*, *YNR066C*), and engineered gene deletions to investigate their phenotypic effect. We also built hemizygous strains to determine the adaptive value of background variation in putative driver genes, by engineering in or out ancestral or evolved alleles in opposite backgrounds. For pre-existing variants, the test for reciprocal hemizygosity uses one-step PCR deletion with *URA3* as a selectable marker. Starting from haploid versions of the WA and NA strains (either *MAT*a, *ho::HygMX*, *ura3::KanMX* or *MAT*α, *ho::NatMX*, *ura3::KanMX*), we deleted the candidate genes and constructed all possible combinations of reciprocal hemizygous strains (Figure S6). The deletion in the haploid strain was confirmed by PCR

and then crossed with the opposite mating type to generate the hemizygous hybrid strains. To test driver *de novo* mutations, we engineered reciprocal hemizygous deletions for two clones carrying the same background allele with heterozygous *de novo* mutations in *RNR2* and *TOR1*. The gene deletion was performed using the dominant selectable marker *NatMX* and we used Sanger sequencing to identify the deleted allele (wild-type or mutated copy).

1.3 Genetic cross

We sought to measure the fitness contributions of pre-existing and *de novo* mutations using a library of recombinant genomes. To do so, we designed a genetic cross where both ancestral and evolved genetic backgrounds were re-shuffled in new combinations and tested for fitness with and without drugs. The genetic cross included the parents, ancestral and evolved isolates. The WA and NA haploid parents were used in *MAT*a, *ura3* and *MAT*α, *lys2* configurations. We derived haploid lines by sporulation on KAc medium from the ancestral and evolved clones. Only tetrads with four viable spores were chosen for continuation in the experiment. Spores were genotyped for mating type (*MAT*a, *MAT*α) using tester strains and for auxotrophies (*ura3*, *lys2*) by plating on dropout medium. We chose spores from tetrad configurations with the mating marker co-segregating as *MAT*a, *ura3* or *MAT*α, *lys2*, allowing a systematic cross between all strains of opposite mating type. We then determined whether each spore inherited the wild-type or the mutated allele by Sanger sequencing of the candidate gene.

Eight ancestral haploid segregants (4 *MAT*α, *lys2* and 4 *MAT*a, *ura3*) were randomly isolated from the ancestral population. For the hydroxyurea environment, we probed individually beneficial *de novo* mutations in *RNR2* (Y169H) and *RNR4* (R34I), which reside on different chromosomes of the *S. cerevisiae* genome. The *RNR2* mutant was isolated from WAxNA F12 1 HU 3 (clone C3) and the *RNR4* mutant from WAxNA F12 2 HU 1 (clone C1) at $t = 32$ days. For rapamycin, three evolved clones isolated at $t = 32$ days were used: one clone with no identifiable driver from WAxNA F12 2 RM 2 (clone C1), a homozygous *FPR1* mutant (W66*) from WAxNA F12 2 RM 1 (clone C3); and a heterozygous *TOR1* mutant (W2038L) from WAxNA F12 1 RM 2 (clone C3). For the hydroxyurea experiment, 21 tetrads were taken for crossing (12 for *RNR2* and 9 for *RNR4*) resulting in 84 spores. For the rapamycin environment, 25 tetrads were used (1 without driver, 4 for *FPR1*, 20 for *TOR1*), resulting in 100 spores.

A genetic cross of size 48×48 in hydroxyurea yielded 2,304 hybrids, and 56×56 in rapamycin, giving 3,136 hybrids. We performed the genetic cross using the Singer RoToR HDA robot on YPD plates (see 'Media composition'). Subsequently, the hybrid populations were grown for two rounds on minimal medium to ensure colonies of solely diploid cells and avoid haploid leakage. A small number of crosses were not successful due to mating inefficiency or slow growth (56 in hydroxyurea and 654 in rapamycin), leaving a total of 2,248 and 2,482 hybrids, respectively. This

was due to mistyping of the mating locus in one *FPR1* spore and three *TOR1* spores, which were excluded together with their derived hybrids. Phenotypic measurements of the crosses were carried out using the high-throughput method of yeast colony growth described in 'Growth phenotyping'.

1.4 Luria-Delbrück fluctuation assay

We performed a fluctuation test to determine the rate of loss-of-heterozygosity (LOH) in different backgrounds, by following the loss of a heterozygous *URA3* marker that results in 5-FOA resistant colonies (Lang and Murray, 2008; Luria and Delbrück, 1943). In all strains tested the *URA3* gene was deleted from its native location in chromosome V and inserted in the *lys2* locus (*lys2::URA3*) in chromosome II ($\sim$470 kb). This genotype is the same used in the crossing phase and therefore shared by all individuals in the population. Our system does not have dedicated markers to distinguish different mechanisms leading to LOH but instead gives an aggregate measurement of the total LOH rate at the *URA3* locus (Figure 5B). The strains were first patched in dropout medium minus uracil and then streaked for single colonies in plates with YPD or YPD supplemented with the drugs (HU at $10\,\mathrm{mg\,mL^{-1}}$ or RM at $0.025\,\mathrm{g\,mL^{-1}}$). Colonies were grown for 3 days at $30\,^{\circ}\mathrm{C}$. Cells were resuspended in water and cell concentration was measured by flow cytometry to obtain a correct dilution factor in the subsequent plating. Cells from each replicate were plated in YPD to determine the total number of colony-forming units (CFUs), and in 5-FOA plates ($1\,\mathrm{g\,L^{-1}}$) to count the number of *URA3*-defective CFUs. For each genetic background, we confirmed the loss of the *URA3* marker in 96 colonies by diagnostic PCR. Four replicates per experiment were used to determine the LOH rate.

To ensure the absence of meiotic spores we inspected $\sim$100 cells per sample. This control was introduced for two reasons. First, the NA parent is a very fast and efficient sporulator (Gerke et al., 2006). We observed the induction of meiosis even without the specific KAc environmental signal required in the laboratory strain S288C (and its derivatives) to initiate sporulation. Second, rapamycin has been shown to promote sporulation by modulating the nutrient sensing pathway (Zheng and Schreiber, 1997). In contrast, hydroxyurea is a very potent meiotic inhibitor. We did not observe fully formed meiotic spores throughout the experiment, though we cannot exclude that meiotic events before the meiotic commitment point (e.g. double-strand breaks) may have occurred that could affect the LOH rate. Therefore, whilst meiotic sporulation can play an adaptive role to reveal recessive mutations (Figure 5), it is most likely neutral on its own in both stress environments.

1.5 Growth phenotyping

To carry out phenotype measurements we used a high-resolution scanning platform, Scan-o-matic, to monitor growth in a 1,536-colony design on solid agar medium (Zackrisson et al., 2016). Solid

media plates designed for use with the Singer RoToR HDA robot (Singer Ltd) were used throughout the experiment. Casting was performed on a leveled surface, drying for $\sim$1 day. We designed a randomized experimental layout by distributing genotypes of interest over 1,152 positions across each plate, keeping every fourth position for 384 controls used for removal of spatial bias. Controls were interleaved in the pre-culture step using a custom-made RoToR pinning program.

We recorded phenotypic measurements using high-quality desktop scanners (Epson Perfection V700 PHOTO scanners, Epson Corporation, UK) connected via USB to a standard desktop computer. Scanner power supplies were separately controlled by power managers (GEMBIRD EnerGenie PowerManager LAN, Gembird Ltd, Netherlands) that immediately shut down the scanner lamp between scans. Images were acquired using SANE (Scanner Access Now Easy). We performed transmissive scanning at 600 dpi using 8-bit grey scale, capturing four plates per image. Plates were fixed by custom-made acrylic glass fixtures. Orientation markers ensured exact software recognition of fixture position. Each fixture was calibrated by the scanner using a calibration model that provided positions for each feature of the fixture, relative to its orientation markers. Pixel intensities were normalized and standardized across instruments using transmissive scale calibration targets (Kodak Professional Q-60 Color Input Target, Kodak Company, USA). Scanners were maintained in a high-humidity environment at 30 °C (incubation room) and kept covered in custom-made boxes during experiments to avoid light influx and minimize evaporation.

Experiments were run for 3 days and scans were continuously performed every 20 minutes. Each image stack was processed in a two-pass analysis. The first-pass was performed during image acquisition and was responsible for setting up the information needed for growth estimations. Positions in each image were matched to the fixed calibration model using the fixture orientation markers, allowing detection and annotation of plates and transmissive scale calibration strips. In the second-pass analysis, images were segmented to identify the location of the plate and the transmissive scale calibration strip. The calibration strips were trimmed and the pixel intensities compared to the manufacturer's supplied values, such that normalized pixel values remained independent of fluctuations in scanner properties over time and space. The colonies were detected using a virtual grid across each plate based on pinning format, and the grid was adjusted for the intersections to match the center of the features detected. At every intersection, each colony and the surrounding area were segmented to determine the local background and pixel intensities. Differences in pixel intensity were converted to population size estimates by calibration to independent cell number estimates (spectrometer and FACS). Based on these, we obtained growth curves in physical units.

Raw measurements of population size $n(t)$ were smoothed in a two-step procedure. First, a median filter identified and removed local spikes in each curve. Second, a Gaussian filter reduced the influence of remaining local noise. Since we expect a population to double in size during the average time taken to progress through the cell cycle, we use an exponential growth model defined

as $n(t) = n(0)e^{\lambda t}$, where λ is the absolute growth rate. If the time that has passed is exactly the doubling time τ, it is trivial to show that within this time span the growth rate can be rewritten as $\lambda = \frac{\ln 2}{\tau}$. It then follows that the absolute growth rate λ can be obtained from the linear fit of any two log-transformed measurements of $n(t)$ in exponential phase, according to $\lambda = \frac{\ln n(t_f)/\ln n(t_i)}{t_f - t_i}$. Therefore, we define the absolute growth rate as the maximum rate during exponential phase, which we estimate by the steepest slope using local regression over five consecutive measurements of $n(t)$. For quality control, the residuals of the model are then used to determine goodness-of-fit and to flag growth curves suspected to be of poor quality, which are visually inspected for artefacts. Rejection rates averaged approximately 0.3% across experiments.

To account for systematic errors, we used an isogenic control at every fourth position in each plate. The fitter of the two parental strains (NA) was chosen as the isogenic control to ensure sustained and reproducible growth across the plate that would enable us to subtract systematic errors. We defined a two-dimensional reference matrix of the 384 controls (on each 1,536 plate) to correct for structured spatial bias in growth rate estimates. Controls with extreme values were removed and the remaining control positions were used to interpolate a normalization surface. This surface was first smoothed with a kernel filter to exclude any remaining noisy measurements, and then by Gaussian smoothing to soften the contours of the landscape. For a colony measured at position (i, j), the absolute growth rate was rescaled by taking the log-transformed difference between the observed estimate and the growth of the normalization surface, i.e. the relative growth rate is then $\lambda_{ij} \to \log_2 \frac{\lambda_{ij}}{\lambda_{ij}^{\mathrm{norm}}}$.

1.6 Media composition

During the crossing phase, the cells were expanded and maintained in YPD medium (1% yeast extract, 2% bacto peptone, 2% D-glucose and 1.7% agar). WAxNA F_1/F_{11} populations were sporulated in solid KAc medium (2% potassium acetate and 2% agar). WAxNA F_2/F_{12} populations were then selected in minimal medium lacking uracil and lysine (0.67% of yeast nitrogen base (YNB), 2% glucose and 0.2% of dropout mix minus uracil and lysine). The selection phase of the experiments was carried out in YPD medium supplemented with the drug. All selection experiments with drugs (as well as follow-ups) used media supplemented with hydroxyurea (HU) at $10\,\mathrm{mg\,mL^{-1}}$ or rapamycin (RM) at $0.025\,\mathrm{g\,mL^{-1}}$, supplied by Sigma-Aldrich.

As part of the follow-up assays, we used antibiotic resistance as a selectable marker to engineer gene deletions and build hemizygous strains, plating in YPD supplemented with the corresponding antibiotic (see 'Engineered genetic constructs'). We supplemented YPD medium with nourseothricin (Nat) at $100\,\mathrm{g\,mL^{-1}}$, hygromycin B (Hyg) at $200\,\mathrm{g\,mL^{-1}}$ and G418 at $400\,\mathrm{g\,mL^{-1}}$. Transformations of reciprocal hemizygous strains also relied on *URA3* as a selectable marker and were plated in minimal medium lacking uracil (0.67% YNB, 2% glucose and 0.2% dropout mix mi-

nus uracil). The fluctuation assay was carried out in YPD, or YPD supplemented with the drug (see 'Luria-Delbrück fluctuation assay'). Colonies defective in the *URA3* allele were selected in 5-FOA plates (YPD medium supplemented with 5-fluoroorotic acid at $1\,\mathrm{g\,mL^{-1}}$). In the genetic cross, the clones used were sporulated in solid KAc medium described above (see 'Genetic cross'). Haploid strains were derived from dissected spores and genotyped for their mating type, *URA3*/*LYS2* auxotrophies and known *de novo* mutations. Strains were crossed in YPD and selected in minimal medium depleted of uracil and lysine.

Growth phenotyping was performed on solid medium using Singer PlusPlates (Singer Ltd). Each plate was cast with 50 mL of synthetic complete (SC) medium, composed of 0.14% YNB, 0.5% ammonium sulphate, 0.077% Complete Supplement Mixture (CSM, ForMedium), 2% (w/v) glucose and pH buffered to 5.8 with 1% (w/v) succinic acid. The medium was supplemented with $20\,\mathrm{g\,L^{-1}}$ of agar. Due to the need for quantitative measurements we chose SC over YPD medium for phenotyping. Measurements in SC and YPD should be comparable since both environments are nutrient rich, though we cannot exclude potential interactions of SC or YPD with the different drugs. However, we deemed the potential error associated with this difference in medium to be substantially less than the error due to systematic biases when using YPD for high-throughput phenotyping (see 'Growth phenotyping').

2 Theory and data analysis

2.1 Sequence analysis

Short-read sequences were aligned to the *S. cerevisiae* S288C reference genome (Release R64-1-1, downloaded from the Saccharomyces Genome Database on February 5, 2011). Sequence alignment was carried out with Stampy v1.0.23 (Lunter and Goodson, 2011) and local realignment using BWA v0.7.12 (Li and Durbin, 2009). After removing PCR duplicates, the median genome-wide DNA coverage was 94× across whole-population samples, 23× across ancestral isolates and 30× across evolved isolates (ranging from 9× to 150×; first quartile 24× and third quartile 91×).

We detected single-nucleotide variants where the WA and NA parents differ, which comprises the background variation segregating in the cross (52,466 sites). We obtained allele counts on these loci using GATK UnifiedGenotyper v3.5-0-g36282e4 (DePristo et al., 2011). These counts were polarized to report WA alleles at each locus, as neither of the parents is the reference genome. The allele counts for segregating variants were first processed using the filterHD algorithm, which takes into account persistence along the genome due to linkage and allows for jumps in allele frequency if there are emerging subclones in the populations.

To detect *de novo* mutations we used three different algorithms: GATK UnifiedGenotyper v3.5-0-g36282e4 (DePristo et al., 2011), Platypus v0.7.9.1 (Rimmer et al., 2014) and SAMtools v1.2-10

(Li, 2011). We focused on single-nucleotide variants (SNVs) and small insertions and deletions (indels). We first performed calling of both SNVs and indels for all ancestral isolates, evolved isolates and the parents. Using BCFtools (Li, 2011), we subtracted parental variation from all derived samples (ancestral and evolved), and excluded variation found in ancestral isolates from all evolved samples to account for segregating variation that was missed. We then required to see a given variant in more than six reads, be covered by more than ten reads and pass the default flags for the algorithms. For clonal isolate sequences, we further required that only a single alternative allele is observed. We then used GATK UnifiedGenotyper to genotype variants identified by at least two of the algorithms. For whole-population sequences, we allowed calls reporting more than one allele and we changed Platypus filtering to allow also 'allele bias' calls. To detect allele frequency changes over time, we only considered loci where the minimum variant allele count across time points was less than two and the maximum more than six reads. To avoid an increase of false positives in whole-population samples, we used more stringent filters on mapping and base quality biases and goodness of fit than for isolate samples. This is particularly important in complex regions where subclonal heterogeneity (e.g. due to variation in copy number) could cause difficulties in calling mutations. Finally, to increase our sensitivity of detection of putative *de novo* variants in recurrent target genes, we kept mutations in *CTF8*, *RNR2*, *RNR4*, *FPR1* or *TOR1* that were only called by a single algorithm.

2.2 Genome-wide scan of pre-existing variants under selection

We observed patterns of selective sweeps when a driver allele with a significant fitness advantage starts to gain in frequency due to the selective pressure applied (Figures 2 and S2). This movement also causes allele frequency changes at nearby loci containing passenger alleles that are genetically linked with the driver, in a process called genetic hitchhiking.

To discern drivers and passengers, we consider a model of a population evolving in a regime of strong selection, where there is a favored allele (driver) at locus i, and a set of linked passengers. We have previously developed a computational approach to analyze selection acting on pre-existing genetic variation that results from a cross (Illingworth et al., 2012). Genetic drift plays a negligible role for allele frequency changes in the selection phase as the population size ($\sim 10^7$ cells) is much larger than its duration ($\sim$54 generations). Therefore, we can assume that the allele frequencies change deterministically and the remaining noise is due to sampling caused by finite sequencing depth.

A selective sweep is then well approximated by a model of the frequency x_i^{WA} of the WA allele at locus i which satisfies the logistic equation,

$$\frac{dx_i^{\mathrm{WA}}}{dt} = \frac{\sigma_i}{2}\, x_i^{\mathrm{WA}}(1 - x_i^{\mathrm{WA}}). \tag{S1}$$

The frequency of the NA allele at locus i is $x_i^{\mathrm{NA}} = 1 - x_i^{\mathrm{WA}}$. Here, the selection coefficient σ_i is the fitness difference $f_i^{\mathrm{WA}} - f_i^{\mathrm{NA}}$ between the alleles, and the pre-factor reflects a diploid population with additive selection. This growth model is a deterministic approximation to the stochastic evolution of $x_i(t)$, which is commonly described by the Wright-Fisher model with directional selection.

To account for the effects of linkage between mutations, we consider a model with two alleles possible at each locus, in which the driver mutation is at locus i and passengers at loci j. We refer to the two alleles at the i locus as $a \in \{\mathrm{WA}, \mathrm{NA}\}$, and the alleles at the j loci as $b \in \{\mathrm{WA}, \mathrm{NA}\}$.

According to our model, the dynamics of passenger alleles are fully specified by the motion of the local driver. The effect of the selected allele on existing variation at a passenger locus j is given by

$$x_j^b(t) = \sum_{a \in \{0,1\}} x_i^a(t) \frac{x_{ij}^{ab}(t_0)}{x_i^a(t_0)}, \quad \text{with} \quad j \neq i \tag{S2}$$

where the two-locus haplotype frequency is $x_{ij}^{ab}(t_0) = x_i^a(t_0) x_j^b(t_0) + (-1)^{a+b} D_{ij}$, and D_{ij} denotes linkage disequilibrium.

We note that due to short-read sequencing of a mixed population we cannot directly measure the two-locus haplotype frequency x_{ij}^{ab} or linkage disequilibrium D_{ij}, but we can parameterize D_{ij} in terms of the recombination which took place during the crossing phase. After N_c generations of sexual recombination, linkage disequilibrium is given by $D_{ij}(t) = (1 - \rho_{\mathrm{tot}})^{N_c} D_{ij}(t_0)$, where the total recombination rate depends on the distance between the loci Δ_{ij} in base pairs (bp) and the local recombination rate ρ in units of $\mathrm{bp}^{-1}\,\mathrm{gen}^{-1}$.

Therefore for a given driver locus and a set of passengers the model is fully specified by the strength of selection, the pairwise linkage structure (or recombination landscape), and allele frequencies at both driver and passenger loci at $t = 0$ days.

We learn these parameters via a maximum likelihood approach with a binomial noise model accounting for sequencing noise. We would like to carry out a systematic driver scan, rather than using a search heuristic for proposing candidate driver locations. To achieve this, we first parameterized the linkage structure with a recombination landscape inferred for this cross in a separate study (Illingworth et al., 2013), avoiding the need to estimate a local recombination rate from allele frequency changes. Secondly, we use the posterior mean of the allele frequency at $t = 0$ days, as obtained with the filterHD algorithm, to fix the initial condition. Setting the initial frequency factors out any frequency deviations of either allele that took place during the crossing phase, which are due to selection on differential mating efficiency. As a result, for each driver-passenger model we only need to learn the strength of selection and we can therefore systematically scan through each of the 52,466 segregating sites, testing the alleles at each locus to be under selection. The

resulting log-likelihood score is compared to a null model where selection on the driver locus is set to zero. This null model corresponds to no frequency changes during the experiment and does not have any parameters to be learned.

We performed a systematic driver scan including passengers within variable window sizes ± 2 kb, 5 kb, 10 kb, 30 kb, 50 kb. Emerging subclones result in global allele frequency changes that supersede the local signal, which is the hallmark of selection acting on pre-existing variation. In consequence, we only considered time points when populations had not yet become clonal, up to $t = 4$ days. For each scan we selected the top 200 loci (out of 52,466) and then required that a given window was identified to be among the top scoring ones in at least two populations. The remaining windows were merged if their passenger loci overlapped. Finally, we required that the region was not identified among those scoring highly in the control environment.

The scan identified a region of interest for rapamycin resistance, found in chromosome VIII (460–490 kb) as discussed in the main text. The signal is visible in all rapamycin populations but not in the control. However, we were not able to localize it fully due to a low recombination rate in this region and possibly also caused by the presence of multiple drivers. The top hits with different passenger window sizes show substructure in terms of peak location. Smaller windows contain multiple peaks, which then get merged to single peaks in larger windows. We note that theoretically the passenger window size should not matter provided the linkage model is adequate and there are no multiple drivers affecting the passenger dynamics. In summary, the region as a whole has strong support across populations to contain pre-existing variation where NA allele(s) are beneficial in rapamycin, albeit we cannot statistically map the signal more finely. We followed up two candidate genes in the region (*CTF8* and *KOG1*), and we validated *CTF8* to have a resistance phenotype (see 'Validation of putative driver genes'). *KOG1*, which is part of the target-of-rapamycin (TOR) pathway, harbors pre-existing missense variants in the population and is thus a plausible target of selection. We did not find regions that replicated across all populations in hydroxyurea.

2.3 Reconstruction of subclonal composition

In the late stages of the selection experiment we identified global allele frequencies changes of pre-existing, segregating variants caused by one or multiple *de novo* mutations (or a particularly favorable combination of the background variation itself) in subclones that are under positive selection. During the selection phase, which is asexual, mutations in the genome of a cell are physically linked. Thus after a cell acquires a beneficial *de novo* mutation this can outweigh all its background variants, which become passengers (they may of course contribute to the fitness of that cell as well). At the genomic level, such an expanding subclone leaves a large imprint on the data at polymorphic sites, with long-range correlations reflecting the genotype of the cell hit by the beneficial *de novo* mutation. This signal with global, long-range correlations and sudden jumps

corresponding to the expanding genotype is qualitatively different from the signal resulting from the localized sweep picture discussed in the previous section.

In this section, we describe how we extend and use the cloneHD algorithm (Fischer et al., 2014) to reconstruct the emerging subclone dynamics in a cell population. The cloneHD algorithm was developed to explain data from short-read DNA sequencing experiments of mixed cell populations (read depth and variant counts) under the following assumptions: (i) The cells evolve asexually (without recombination). This ensures that there are long-range correlations along the genome, which can, in principle, be reconstructed from short-read data. (ii) The population consists of a mixture of subclones, i.e. groups of genetically identical cells. The total number of subclones and their relative fractions in the population are unknown. The number of subclones, which can be reconstructed from real data, is small and depends on how different they are and what their population fractions are. (iii) Each subclone carries a unique copy number profile and genotype. Both of which are unknown. (iv) There is a distinct bulk component of the population which differs from the subclones, e.g. by having a different set of genotypes. Its fraction is also unknown. (v) When several samples are jointly analyzed, the same subclonal populations are assumed to be present in all samples. However, their frequencies in some of the samples can be zero.

Previously, cloneHD was used to explain subclonal heterogeneity found in human cancers. With a few extensions, this methodology can also be used for the yeast evolution experiment studied here. After the crossing phase, the populations evolve asexually under selective pressure. The rounds of crossing of the two original strains have produced a diverse pool of recombinants, where the genotype of each cell is – for all practical purposes – unique. This ancestral population of diploid cells is modeled here as the bulk component. Its allele frequency profile can be seen in Figures 2 and S2.

At the later stages of the evolution, a small number of individual yeast cells start to outgrow the rest of the population, maybe due to a beneficial combination of pre-existing variation or due to *de novo* mutations. These cells grow clonally to measurable fractions of the population and leave their fingerprint in the allele frequency profile genome wide. In the extreme case, a single cell grows clonally to take over the entire population and its individual genotype that can be directly observed in the sequencing data. In the general case, there will be a mixture of subclones and bulk population as described above. As an added complication, subclone copy number profiles need not be pure diploid.

This scenario is already covered in principle in the model underlying cloneHD (see Section 4 in the Supplemental Information of Fischer et al. (2014)). In the current study, the population is sequenced at several time points such that there are multiple related samples available for inference

with cloneHD. For the read depth N_i^t at locus i and time point t, the emission probability is

$$N_i^t \sim \text{Pois}\left(N_i^t \,|\, M^t \, \langle c\rangle_i^t\right), \tag{S3}$$

$$\text{with} \quad \langle c\rangle_i^t \equiv c_0\,(1-F^t) + \sum_{j=1}^{n} c_{ij}\, f_j^t \tag{S4}$$

where M^t is the mean sequencing depth per haploid DNA, c_{ij}^t is the total copy number of subclone j at locus i, c_0 is the total copy number of the reference compartment (2 for diploid) and f_j^t is the frequency of subclone j (with $F^t \equiv \sum_{j=1}^n f_j^t$).

The number of WA reads n_i^t determines the observed allele frequency x_i^t and is assumed to be binomially distributed

$$x_i^t \approx \frac{n_i^t}{N_i^t}, \quad \text{with} \quad n_i^t \sim \text{Bin}\left(n_i^t | N_i^t,\, x_i^t\right) \tag{S5}$$

$$\text{where} \quad x_i^t \equiv \frac{\langle g\rangle_i^t}{\langle c\rangle_i^t}, \quad \text{with} \quad \langle g\rangle_i^s \;\equiv\; x_i^{t_0}\left(1-F^t\right) + \sum_{j=1}^{n} g_{ij}\; f_j^s \tag{S6}$$

where g_{ij} is the genotype of subclone j at locus i and $x_i^{t_0}$ is the initial allele frequency spectrum. The only substantial difference to the situation in the cancer setup is that here the genotype of a particular subclone j is persistent across large regions of the yeast genome, reflecting the haplotype structure resulting from the cross. In cancer, these correlations along the genome are missing since the aforementioned model is only applied to somatic point mutations which occur randomly along the genome. Altogether, the subclonal structure of the yeast cell populations can be reconstructed with cloneHD in `cna+snv` mode, where both the CNA and SNV data are modeled with persistence along the genome. The rest of the cloneHD workflow fully applies. First, the read depth and the allele frequency data are analyzed with filterHD, thus finding a segmentation of both data tracks for all samples jointly (in later stages subclones are larger and the transition points become more prominent). This information and the initial allele frequency profile $x_i^{t_0}$ are provided to cloneHD together with the read depth and pre-existing variant allele data in `cna+snv` mode. The maximum likelihood set of subclonal genomes (including their copy number profiles and genotypes) and their cell fractions is then found by cloneHD at each time point. Figure S1 shows the general setup and the cloneHD reconstruction for simulated data in one population.

We assessed the ability of our algorithm to recover several features of interest from simulated jump-diffusion processes over a range of plausible parameters. For each parameter set, we simulate a 1 Mb region with $L = 10{,}000$ observations and 60 reads per locus on average, then compute maximum likelihood estimates using different numbers of subclones. Our choice of jump probability for simulated data is set to 4×10^{-5} per base. This reflects the size of linkage blocks with plausible recombination scenarios during crossing. The clones are added in a chosen background

assuming the bulk has reached a steady profile. We would like to reconstruct three features: (i) the total number of subclones, (ii) their subclonal frequency, and (iii) obtain posterior estimates of subclonal genotypes.

The maximum-likelihood estimates of the subclonal fractions are approximately equal to the true values. The reconstruction is shown in Figure S1B as a black solid line, which is the cloneHD solution for the mean posterior SNV emission rate. We can recapitulate the correct number of breakpoints and their location. The fidelity of our reconstruction to the true subclonal genotype is corroborated by the close correlation between our estimates from whole-population sequencing and the true genotypes derived from clonal isolates.

2.4 Subclonal dynamics resolved by whole-population genome sequencing

To reconstruct the subclonal composition of each WAxNA replicate from whole-population sequencing, we used cloneHD providing the jumps found by filterHD and the posterior mean allele frequencies of the ancestral population to act as a bulk component for the inference (see 'Reconstruction of subclonal composition'). We used cloneHD in `snv` mode, as visual inspection did not reveal copy-number aberrations from whole-population sequencing. For each population, we systematically tested 0–4 subclones and determined the total data likelihoods under each model. The number of subclones per population are summarized in Table S1, together with the time evolution of subclone frequencies in Figure S3. We required a log-likelihood gain greater than 20,000 units for the inclusion of an additional subclone. This conservative cut-off only allows genome-wide signals to be associated with a subclone. This is necessary as the bulk component of the population can also change throughout the experiment. This prevents that, with a less conservative cut-off, other solutions would be favored that would introduce artifactual subclones with suitable genotypes to improve fits in regions where selection acts on the bulk (see 'Genome-wide scan of pre-existing variants under selection').

To ascertain the expansion of subclones throughout the experiment, we determined the allele frequency of *de novo* mutations in WA, NA and WAxNA populations during the selection phase from whole-population sequencing. We found that these mutations typically did not reach detectable frequency (i.e. between 1–5%) until more than 8 days had passed, with steady increases thereafter (Figures S3 and S4). Across populations, we found 66 point mutations by whole-population sequencing spanning 41 unique loci, out of which 50 fall onto coding sequence. These loci contain 32 functional driver mutations: 4 in *RNR2*, 10 in *RNR4*, 11 in *FPR1*, and 7 in *TOR1*. This includes two tri-allelic loci: one corresponding to *FPR1* driver mutations W66* and W66S, and another to a SNV and an insertion in *FPR1*.

2.5 Adaptive *de novo* mutations and genomic instability in clonal isolates

Overall, we identified 91 SNVs and indels in 173 ancestral haploid isolates, and 140 SNVs and indels in 44 evolved diploid isolates. We detected 82 SNVs and 1 insertion across 22 evolved isolates in hydroxyurea (range 1–8 per isolate), containing 10 adaptive mutations in *RNR2* and 12 in *RNR4* (Figure 4A). There were 56 SNVs and 1 deletion across 22 evolved isolates in rapamycin (range 0–6 per isolate), which contained 8 adaptive mutations in *FPR1* and 5 in *TOR1* (Figure 4B). 33 out of 36 mutations detected by whole-population sequencing across WAxNA populations could be found in clonal isolates. All *de novo* driver mutations found by clone sequencing were confirmed by targeted Sanger sequencing. Assuming the ancestral genomes to have passed through $\sim$150 generations during the crossing phase, we estimated a point mutation rate $\mu = 2.89 \times 10^{-10}$ per base per generation; and similarly for evolved genomes going through $\sim$54 generations in the selection phase ($\mu = 5.32 \times 10^{-9}\,\mathrm{bp}^{-1}\,\mathrm{gen}^{-1}$). We detected two instances of cross-contamination between populations, so the derived events in clones isolated from these populations are valid to estimate the mutation rate but should not be counted to have arisen independently.

Sequence analysis revealed that 3 out of 4 unique variants in *RNR2* (N151H, E154G and Y169H) and 2 out of 3 unique variants in *RNR4* (R34G/I) mapped to a conserved domain of the ribonucleotide-diphosphate reductase small chain. *FPR1* mutations occurred at W66, either introducing a premature stop codon or changing to serine. Previous studies indicate that the majority of non-synonymous changes in *FPR1* affect protein stability (Koser et al., 1993). Furthermore, the premature stop at W66 truncated the residue required for rapamycin binding (Y89). We observed clones carrying the W66* mutation selected multiple times from the same founder population indicating a pre-existent individual carrying a heterozygous mutation and independent LOH events that render the loss-of-function mutation homozygous (Figure 4B). All five driver SNVs in *TOR1* (S1972I/R, W2038L/C and F2045L) mapped to the FKBP12-rapamycin-binding (FRB) domain, which is $\sim$100 aa long, providing a mechanistic explanation of the drug resistance (Figure 4B). Previous studies have found dominant mutations in S1972 and equivalent mutations in the mammalian TOR (mTOR) have a similar effect on drug binding. Substitutions at W2038 with a similar dominant effect are equivalent by homology to those previously described in *TOR2* (W2042) (Lorenz and Heitman, 1995).

To identify copy-number aberrations from clone sequencing, we segmented the coverage depth as a function of genomic position with cloneHD. We found one copy number gain (`n>2n`) of chromosome IX in ancestral haploid isolates. Evolved diploid isolates accrued copy number gains (`2n>3n`) in chromosomes VIII, IX and X in hydroxyurea and chromosome IX in rapamycin, as well as whole-genome copy loss (`2n>n`) in rapamycin.

Using background variants as markers, we could detect mis-segregation of chromosomes leading to loss-of-heterozygosity. The presence or absence of the WA or the NA allele provides a

robust signal of heterozygosity or LOH that is not affected by sampling noise in coverage. We used cloneHD to genotype the sequenced isolate samples at segregating sites. We then grouped isolate sequences by subclone lineage, requiring at least 80% genotype similarity to belong to the same lineage. In hydroxyurea, this resulted in 22 isolates stemming from 8 subclone lineages, with more than a single isolate each. In rapamycin, 22 isolates were assigned to 4 subclone lineages, with more than a single isolate each. For each subclone lineage, we inferred its ancestral genotype. In case of a locus with a unique genotype across all isolates we assigned this to be the ancestral state. In all other cases we inferred the ancestral state to be heterozygous, as lost alleles cannot be regained. We then annotated all the isolates from each clone for LOH events. Figure 4 shows the inferred ancestral genotypes and the derived SNVs, indels, LOH events and copy number variants, grouped by population and subclone lineage. Whilst we did not find evidence of copy-number aberrations to be adaptive, we characterized fitness increases associated with LOH caused by changes in the allelic state of pre-existing and *de novo* variants (Figure 3B). To determine the rate of LOH events, we counted the number of independent events within a chromosome that have led to the gain or loss of the ancestral allele in the evolved isolate sequences. This estimate is challenging given that the ancestral states contain both homozygous and heterozygous loci, so that the precise end points of individual LOH events are uncertain. To obtain a lower bound, we counted whether any isolate had undergone LOH affecting $\geq$10 consecutive background variants, for each chromosome in each clone. We found 48 events in hydroxyurea and 24 events in rapamycin (6 per genome per clone). We excluded two haploid individuals from this counting as well as from the length distribution of homozygosity tracts in Figure 5A.

We compared our genome-wide estimates of the point mutation and LOH rates based on the mutation counts in clone genome sequences with locus-specific measurements of the LOH rate using a fluctuation test (see 'Luria-Delbrück fluctuation assay'). We fitted the fluctuation data to a model of the Luria-Delbrück distribution. We determined the average number of LOH events per culture m, such that LOH rate can be estimated by $\mu = \frac{m}{N}$, where N is the average number of cells per culture. To determine the mean number of LOH events m, we used the probability generating function of the Luria-Delbrück distribution defined by Hamon and Ycart (2012). In the control environment, we observed a rate of $\mu = 2.59 \times 10^{-5}$ per generation in the NA background, consistent with previous reports (Barbera and Petes, 2006). We observed an intermediate rate in the WA background ($\mu = 8.01 \times 10^{-6}\,\mathrm{gen}^{-1}$) and the WAxNA F_1 hybrid had an approximately ten-fold lower rate ($\mu = 4.01 \times 10^{-6}\,\mathrm{gen}^{-1}$). These data indicate that LOH rates can vary between genetic backgrounds. There was a sharp increase of LOH rates when colonies were grown in hydroxyurea, irrespective of the background tested. This finding is consistent with previous studies in the laboratory strain S288C reporting that replication stress promotes recombinogenic DNA lesions (Barbera and Petes, 2006). We also observed a background-dependent increase in LOH rate

in the presence of rapamycin, especially in the NA founder. Our estimates of the point mutation rate based on the mutation counts in ancestral and evolved clones ($\sim 10^{-10}\,\text{bp}^{-1}\,\text{gen}^{-1}$) and of the LOH rate based on the fluctuation assay ($\sim 10^{-5}\,\text{gen}^{-1}$), suggest that any recessive genes will be likely to lose the wild-type allele by LOH. Given that the LOH rate is much higher than the point mutation rate and it typically affects large regions (100-1,000 kb, see Figure 5A), recessive mutations can feasibly be 'rescued' by LOH.

2.6 Validation of putative driver genes

To test candidate driver mutations, we measured the growth rate of engineered gene deletions to confirm whether their knockouts are beneficial. We also measured the growth of hemizygous strains to test allelic differences in driver genes with pre-existing and newly acquired mutations. The engineered genetic constructs are listed in Supplemental Tables. We performed $n_r = 64$ replicate measurements of each construct in two independent runs, which were initiated from a single pre-culture plate, evenly distributed over 16 experimental plates and simultaneously run in 4 scanners. The growth rate of each of these strains is shown in Figures S7 and S8, labeled by genetic background b and genotype g.

We deleted one copy of *RNR2* in WA and NA diploids and sporulation of these strains resulted in tetrads with two viable spores and two unviable *rnr2*Δ mutants, indicating that this gene is essential in both backgrounds. *RNR2* is also essential in the laboratory S288C background. Furthermore, the heterozygous deletions of *RNR2* diploids show strong haploinsufficiency for hydroxyurea resistance (Figure S7). In contrast to its interaction partner, *RNR4* is not essential in the laboratory background. However, deletion of this gene in diploid WA and NA backgrounds proved it to be essential in the WA background. The NA strain is viable after deletion, though with severe growth defects. Diploid hemizygous strains for *RNR4* deletions in both backgrounds show increased sensitivity due to dosage effects (Figure S7).

FPR1 and *TOR1* are not essential genes and we performed deletions in both haploids and diploids. *FPR1* directly binds rapamycin inhibiting the TOR pathway and its deletion is highly resistant (Figure S8). Deletion of one copy of *FPR1* does not increase the growth rate in rapamycin, indicating that both copies of the gene need to be inactivated to drive resistance. Consistently with this observation, all mutations observed in *FPR1* are homozygous. Large colonies in the *FPR1* plating assay all acquired double-hit events (*de novo* SNV or indel plus LOH) that inactivated both functional copies of the gene (inset in Figure S8). Estimates of the number of colonies for parent and hybrid backgrounds follow a similar trend to the estimates obtained with the fluctuation test. In contrast, *TOR1* deletion results in high sensitivity to rapamycin and a single deleted copy does not alter the drug response (Figure S8).

Reciprocal hemizygosity tests in ancestral hybrids confirmed background-dependent effects in

CTF8, with strong positive selection acting on the NA allele as predicted by our model of driver-passenger dynamics (Figure S8). *KOG1*, which is a component of the TOR signaling pathway, did not show any allelic differences but deleting either copy caused haploinsufficiency in rapamycin. We also deleted either the wild-type or the mutated allele of evolved mutant clones, generating pairs of clones identical throughout the genome except for the candidate driver mutation. The four genes harboring *de novo* driver mutations do not appear to show allelic differences between the two parental backgrounds as shown by the reciprocal hemizygosity test (Figure S8). No allelic differences were observed for *DEP1*, *INP54* and *YNR066C*, which are confirmed as passengers.

2.7 Fitness distribution and population averaging

To characterize the fitness of cells in a heterogeneous population with multiple subclones, i.e. where several haplotypes may be present, we measured the growth properties of an ensemble of cells. With an ensemble method, we will typically measure the population average. However, since we found subclones co-existing, these may be found in states that are far from the population mean. Hence, we determined the intra-population growth rate of the populations at the start and the end of the selection phase (Figures 3 and S9). For each population, we estimated the probability distribution $P(\lambda^t)$ of the growth rate λ at time t by sampling n_k isogenic individuals. With an ensemble of $n_k = 96$ individuals per time point we took $n_r = 32$ replicate measurements per individual. The replicates were measured in two independent runs, evenly distributed over 16 experimental plates which were initiated from a single pre-culture plate and run in 4 scanners, all in parallel.

We modeled the probability distribution of the data, $\{\lambda_n^t\}_{n=1}^{n_k}$, as a mixture model of normal distributions,

$$P(\lambda^t) = \sum_{k=1}^{K} \pi_k \mathcal{N}\left(\lambda^t | \mu_k, \sigma_k^2\right), \tag{S7}$$

where K is the number of components. We can interpret the mixing coefficients, π, as the bulk and multiple clonal components. We determined the fraction of cells in the fitter, faster clonal state(s) and the slower, bulk component by fitting $p(\lambda)$ to a mixture of normal distributions with $K \in \{1,2,3\}$ components. There are $2K+1$ fitting parameters, which are learned by maximizing the likelihood function $P(\lambda^t)$: the component means $\{\mu_k\}$ and variances $\{\sigma_k^2\}$, and the relative weights between them. In multimodal populations, the weights are in good agreement with the average of two consecutive inflection points surrounding the trough between the bulk and the clonal subpopulations (Figure S9).

2.8 Decomposition of background-averaged fitness effects of mutations

We carried out a genetic cross to reconstruct a fraction of the genotypes that a population can explore and examined the average mutational effect of beneficial variants in multiple genetic back-

grounds. We isolated isogenic individuals from parents, ancestral and evolved populations. As described in the 'Genetic cross' section, we sporulated these diploid cells and selected haploid segregants of each mating type (48 in hydroxyurea and 56 in rapamycin), parameterized by an index a or α. We crossed the *MAT*a and *MAT*α versions to create hybrids. The cross forms a two-dimensional lattice that is conveniently parameterized by the set of lattice positions (a,α).

We obtained a set of measurements for the growth rate λ of individuals, each of which has a unique combination of background genotype b, *de novo* genotype d, sampling time t and auxotrophy x. Every haploid genome being crossed is an independent background indexed by $b_{\{a,\alpha\}} = 1,2,\ldots,n_b$ ($n_b = 48$ in HU and $n_b = 56$ in RM, either a or α), such that reshuffled diploid hybrids are parameterized by $b_{a\alpha}$. Genetic backgrounds are sampled before the cross (parents), before selection starts at $t = 0$ (ancestral) or after $t = 32$ days (evolved), such that $t_{\{a,\alpha\}} = 1,2,\ldots,n_t$ ($n_t = 2$ for the parents; $n_t = 4$ at $t = 0$; $n_t = 42$ in HU and $n_t = 46$ in RM at $t = 32$). We denote *de novo* genotypes by $d_{\{a,\alpha\}} = 1,2,\ldots,n_d$ ($n_d = 12$ for *RNR2*; $n_d = 9$ for *RNR4*; $n_d = 1$ without driver; $n_d = 4$ for *FPR1*, $n_d = 20$ for *TOR1*). Haploid spores are auxotroph and segregate with the mating locus, such that $x_{\{a,\alpha\}} \in \{$*ura3-*, *lys2-*$\}$, whereas diploid hybrids do not have amino acid deficiencies. To estimate the measurement error, we carried out replicate measurements of each unique spore ($n_r = 12$ in HU and $n_r = 6$ in RM) and of each hybrid genotype combination ($n_r = 3$). Replicates were initiated from the same pre-culture plate, evenly distributed over 32 plates that and run in 4 scanners, all in parallel.

The data matrix shows the fitness effect of every *de novo* genotype d at each background b sampled at time t, averaged over measurement replicates and measured relative to the ancestral population (Figure S10). Based on these measurements, we observed that *de novo* mutations are beneficial, yet their associations to genetic backgrounds have idiosyncratic effects. The effects of *de novo* mutations are mediated by background fitness as evidenced by the large phenotypic variance. We note that genetic crosses between different backgrounds need not give rise to a 'symmetric' phenotype data matrix, as we only enforce 2:2 segregation for the mating locus *MAT*a/α. Whilst background variants will co-segregate with the mating locus, *de novo* mutations need not.

To examine the average fitness effects of functional genotypes in hydroxyurea (*RNR2*, *RNR4*) or rapamycin (*FPR1*, *TOR1*), we calculated an ensemble average of the growth rate λ. The ensemble average $\langle\lambda\rangle$ is either taken over single spore backgrounds $b_{\{a,\alpha\}}$ or pairs of hybrid backgrounds $b_{a\alpha}$ with different degrees of relatedness,

$$\langle\lambda\rangle^{td}_{\{a,\alpha\}} = \frac{1}{n_b}\sum_{b=1}^{n_b} \lambda^{btd}_{\{a,\alpha\}} \quad \text{and} \quad \langle\lambda\rangle^{td}_{a\alpha} = \frac{1}{n_b}\sum_{b=1}^{n_b} \lambda^{btd}_{a\alpha}, \tag{S8}$$

where $\langle\cdots\rangle$ denotes the mean over genetic backgrounds. We found that, on average, *RNR2*, *RNR4* and *TOR1* mutations are dominant and highly penetrant (Figures 6D and 6F). In contrast, *FPR1* is recessive, only increases fitness when the mutation is homozygous and carries a fitness cost in the

absence of rapamycin (Figures 6F and S11D, respectively).

We partitioned the variation in fitness contributed by background and *de novo* driver mutations using linear mixed models. To model genetic backgrounds containing beneficial mutations we need to describe how likely a phenotype is in the presence or absence of any mutation. We restricted our model to pairs of individuals that are not closely related to avoid spurious correlations by population structure, so we retained ancestral and evolved individuals and excluded the parents. We are interested in the aggregate effect across all mutations within a spore or hybrid rather than the effects of individual variants. As the data represents a finite sample from the distribution of all possible genetic backgrounds, the background contribution to the phenotype is naturally modeled as a random-effect term (i.e. individual genetic backgrounds are drawn at random from a population, and the variance of the underlying distribution is to be inferred). In addition, other systematic effects that potentially contribute to fitness are modeled as fixed-effect terms: (i) time t when the individual was sampled, i.e. at $t = 0$ (ancestral) or $t = 32$ (evolved); (ii) *de novo* driver mutation status d of the individual, e.g. *FPR1* driver mutation in homozygous state; and (iii) auxotrophy, denoted by x, e.g. *ura3*- or *lys2*-. We implemented four nested linear mixed models outlined below.

Model 1 We first considered a model where we only included the background without other effects. This means that the observed growth rate λ_b for a background b conditioned on the random effect taking a value β_b is distributed as

$$\lambda_b|_{\mathcal{B}=\beta_b} \sim \mathcal{N}\left(\beta_0 + \beta_b x_b, \sigma_\varepsilon^2\right), \tag{S9}$$

where β_0 is a shared constant baseline per background that must be inferred, σ_ε^2 represents measurement noise, x_b is an element from the model design matrix (here 1 for each b as they all are assigned a value). Finally, the background growth rate is distributed as $\mathcal{B} \sim \mathcal{N}\left(0, \Sigma^2\right)$ and its variance Σ^2 is a model parameter to be inferred. We note that for each background b we have multiple measurement replicates of λ_b. Altogether, Model 1 has three modeling parameters, β_0, Σ^2 and σ_ε^2.

Models 2, 3 and 4 Model 2 includes the same factors as Model 1, but the time of sampling t is nested as a fixed effect. Model 3 also accounts for *de novo* driver mutation status denoted by d. In addition, Model 4 includes a fixed effect accounting for amino acid deficiencies (or auxotrophy), denoted by x. Altogether the growth rate λ_{btdx}, conditioned on the random effect taking a value β_b, is distributed as:

$$\lambda_{btdx}|_{\mathcal{B}=\beta_b} \sim \mathcal{N}\left(\beta_0 + \underbrace{\beta_b x_b}_{\text{random}} + \underbrace{\beta_t x_t + \beta_d x_d + \beta_x x_x}_{\text{fixed}}, \sigma_\varepsilon^2\right), \tag{S10}$$

where $\beta_t, \beta_d, \beta_x$ are fixed-effect terms to be inferred and x_t, x_d, x_x are elements of the model design matrix. Compared to Model 1, Models 2, 3 and 4 have extra parameters β_t, β_d, and β_x. The number

of free parameters depends on how many unique levels each factor contains, e.g. how many driver mutations are sampled in the experiment.

The likelihood for a data vector λ given the full model (Model 4) can then be written as

$$\begin{aligned}P(\lambda \mid \text{model}) &= P\left(\lambda \,\middle|\, \beta_0, \beta_t, \beta_d, \beta_x, \Sigma^2, \sigma_\varepsilon^2\right) \\ &= \prod_{\langle a,\alpha \rangle} \prod_{r=1}^{n_r} \int P\left(\lambda_{btdx} \,\middle|\, \beta_b, \beta_0, \beta_t, \beta_d, \beta_x, \Sigma^2, \sigma_\varepsilon^2\right) \\ &\quad \times P\left(\beta_b \,\middle|\, \Sigma^2\right) d\beta_b\end{aligned}$$

where the integrand is the product of the probability density given by Equation S10 and the posterior distribution over the random effects.

Next, we applied all four models to the phenotypes of the genetic cross: a genetic cross based on hydroxyurea selection, measured in hydroxyurea and a control environment; and a genetic cross based on rapamycin selection, measured in rapamycin and a control environment, both for spores and hybrids. We fitted each model using restricted maximum likelihood with the R-package lme4 (Bates et al., 2015), summarized in Table S2. Using Akaike's Information Criterion (AIC) for model selection all conditions had a score supporting Model 4 apart for those selected and measured in hydroxyurea, where both spores and hybrids supported Model 3. We compared the fitted and observed values and in all cases the fits were good, as shown in Figure S12 for Model 4.

We can assess the overall goodness-of-fit of the models by the proportion of variance explained. In particular, we would like to know the contribution of various model components to the overall fit, and to do so we obtain separate measures for the partial contributions of fixed and random effects (Gelman and Hill, 2006)

$$r^2 = \frac{\sigma_F^2 + \sigma_R^2}{\sigma_F^2 + \sigma_R^2 + \sigma_\varepsilon^2}, \tag{S11}$$

where σ_R^2 is the variance contribution by random effects, any incremental fixed effect contributes additively to the fixed-effect variance, s.t. $\sigma_F^2 = \mathrm{Var}(\beta_t x_t + \beta_d x_d + \beta_x x_x)$, and r^2 represents the proportion of variance explained by the fixed and random effects combined. Dropping the σ_R^2 term from the numerator, we can evaluate r^2 and the fixed-effects variance r_F^2 for linear mixed models, and estimate the background contribution to the variance by $r^2 - r_F^2$. Then to further assign the fixed-effect variances to individual variance components shown in Figure 6B, we used the simpler models and their estimated r_F^2. We note that modeling the background component using fixed effects instead leads to a variance decomposition that is nearly identical to the decomposition with linear mixed models described here. However, modeling the background as a fixed effect requires fitting a large number of parameters (one extra parameter per background) and thus describing the background by random effects is a better model for the data.

Supplemental Figures

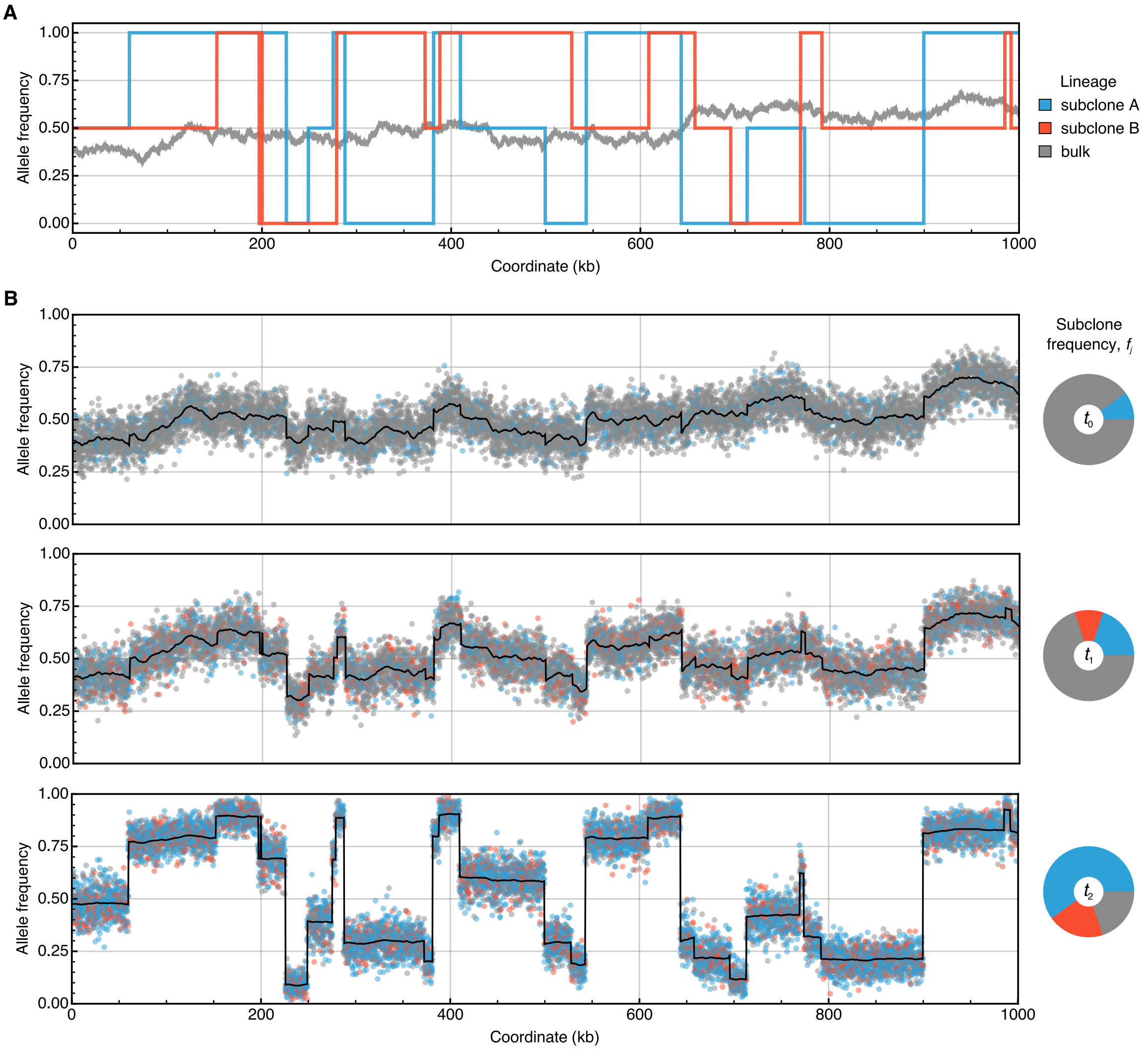

Figure S1: Subclonal reconstruction of a simulated example. Related to Figures 2, 3A and 3C. Subclonal reconstruction for a simulated example of two macroscopic subclones using cloneHD. The physical locations of the segregating sites are represented along the x axis and the y axis shows the allele frequency at every locus. (**A**) The true allele frequency of the bulk (grey) and the true genotypes g_{ij} of two subclones (blue and red) at locus j for the simulated example. (**B**) Simulated jumps (subclones) and diffusion (bulk), in the presence of two subclones of size $f_j = (0.1, 0.0)$ at t_0, $f_j = (0.2, 0.1)$ at t_1 and $f_j = (0.6, 0.2)$ at t_2. The path along the genome is described by a mixture of the two, with jump probability $p = 4 \times 10^{-5}\,\mathrm{bp}^{-1}$, diffusion constant $\sigma = 5 \times 10^{-4}$, and binomial draws as emissions. The simulated observations are probabilistically color-coded according to the bulk fraction or each of the subclone fractions. The mean posterior estimate of the SNV emission in black solid line shows the accuracy of the reconstruction. The pie charts indicate the inferred bulk and subclone frequency estimates, $\hat{f}_j$.

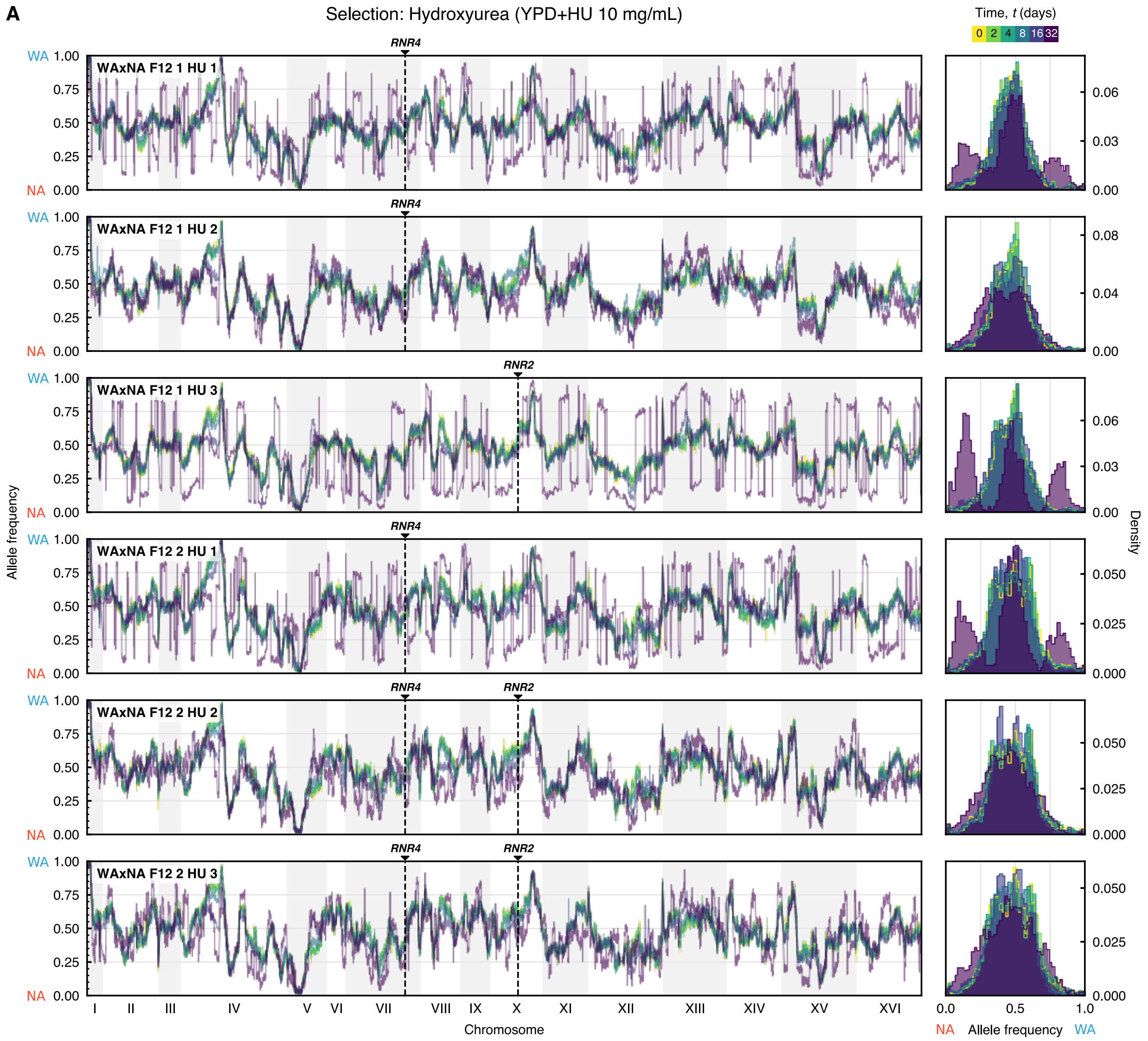

Figure S2: Genome-wide allele frequency changes of pre-existing genetic variation. Related to Figures 2, 3A and 3C. Time series of genome-wide frequencies of pre-existing, parental variants after $t = (0, 2, 4, 8, 16, 32)$ days, measured by whole-population sequencing. From top to bottom, replicate populations were evolved in (**A**) hydroxyurea, (**B**) rapamycin and (**C**) a control environment. Left panels: Chromosomes are ordered along the x axis; the frequency of the WA allele at locus i, x_i^{WA}, is shown for 52,466 pre-existing variants on the y axis, colored by time point. The reciprocal frequency of the NA allele is equivalent since $x_i^{\mathrm{NA}} = 1 - x_i^{\mathrm{WA}}$. Allele frequencies are estimated from the mean posterior probability given by the filterHD algorithm. Pre-existing and *de novo* driver mutations are highlighted by dashed lines. Right panels: Changes in the allele frequency spectrum across time.

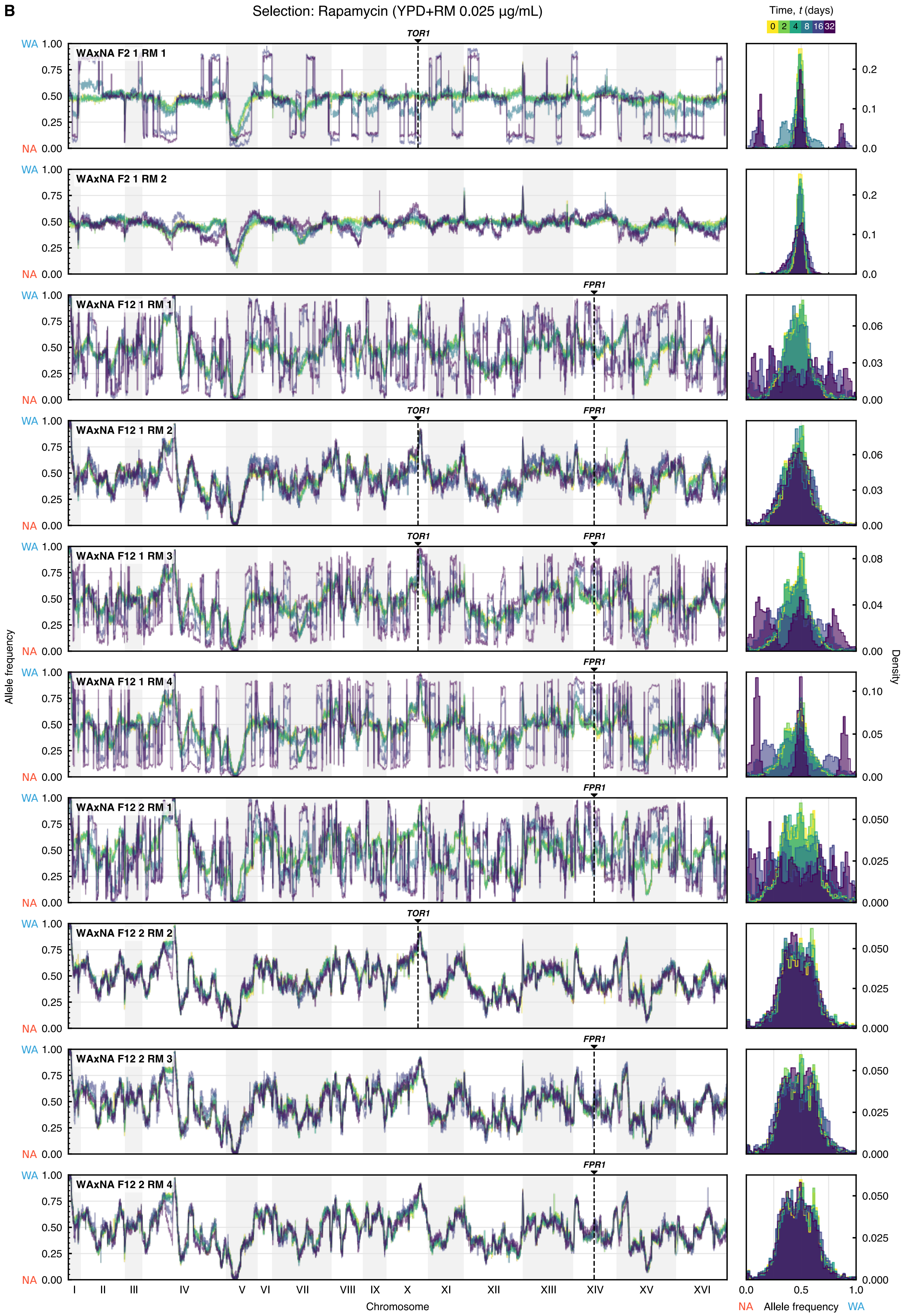

Figure S2: (continued)

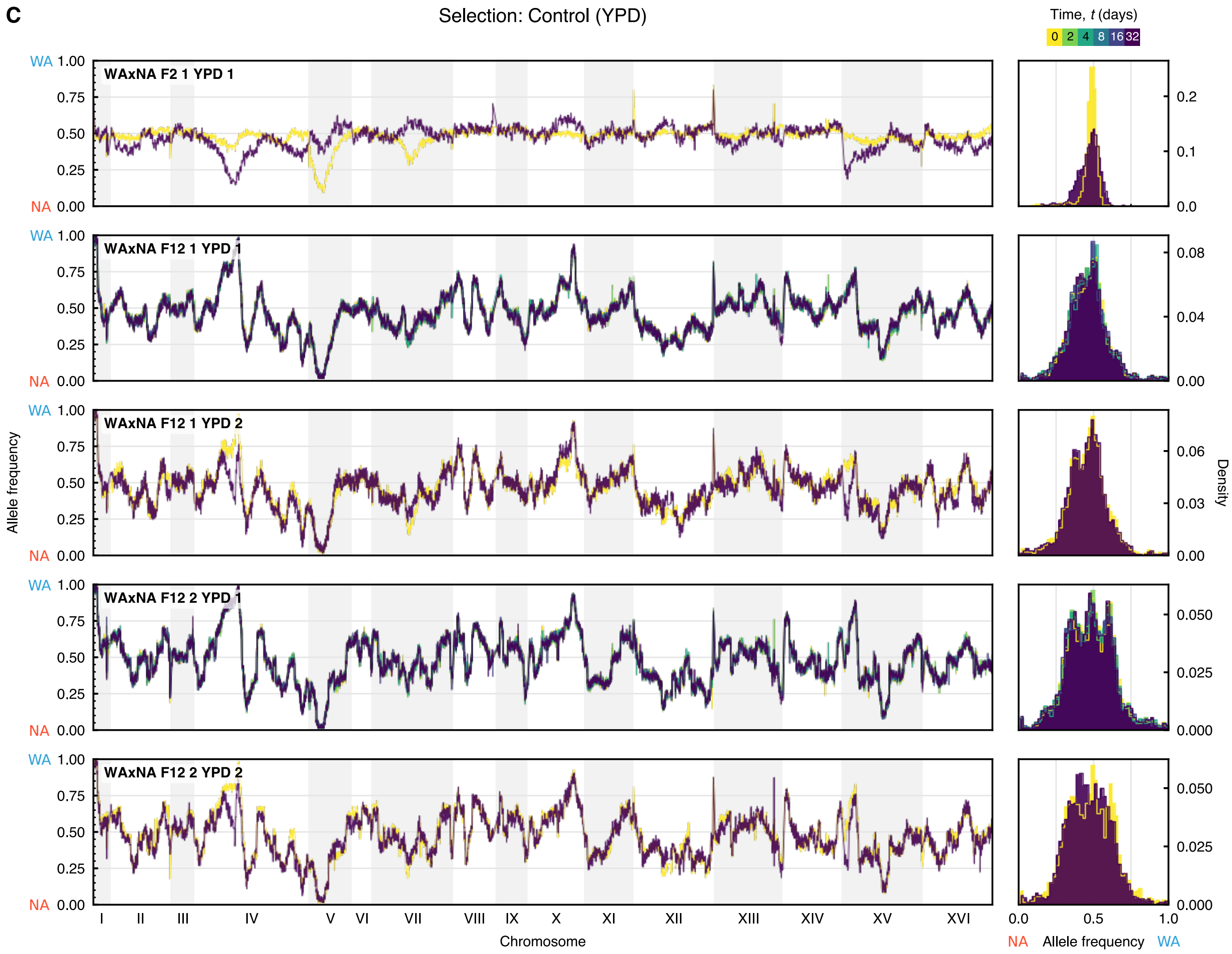

Figure S2: (continued)

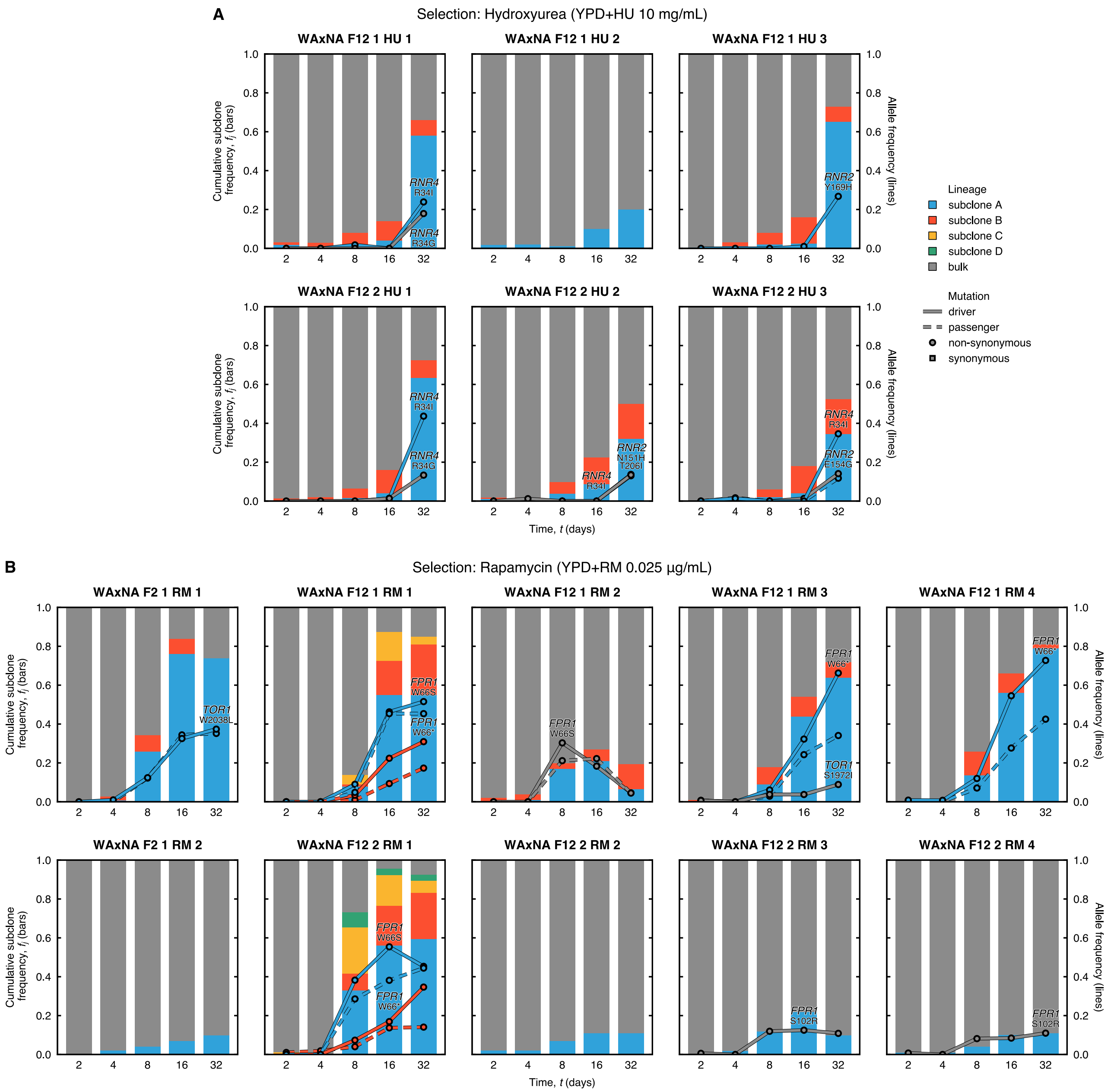

Figure S3: Subclonal dynamics in WAxNA populations. Related to Figures 3A and 3C. Subclonal dynamics in time for WAxNA founders evolved in (**A**) hydroxyurea and (**B**) rapamycin, measured by whole-population sequencing. Time is on the x axis, starting after crossing when the population has no competing subclones. Cumulative haplotype frequency of subclones (bars) and allele frequency of *de novo* mutants (lines) are on the y axis. The subclone frequencies are inferred from the frequency of pre-existing variants using cloneHD (see Figure S2). Driver mutations are solid lines and passenger mutations are dashed lines, colored by subclone assignment; circles and squares denote non-synonymous and synonymous mutations, respectively. For driver mutations, the mutated gene and codon are indicated above each line. No macroscopic subclones or *de novo* mutations were detected in any of the control replicates in YPD.

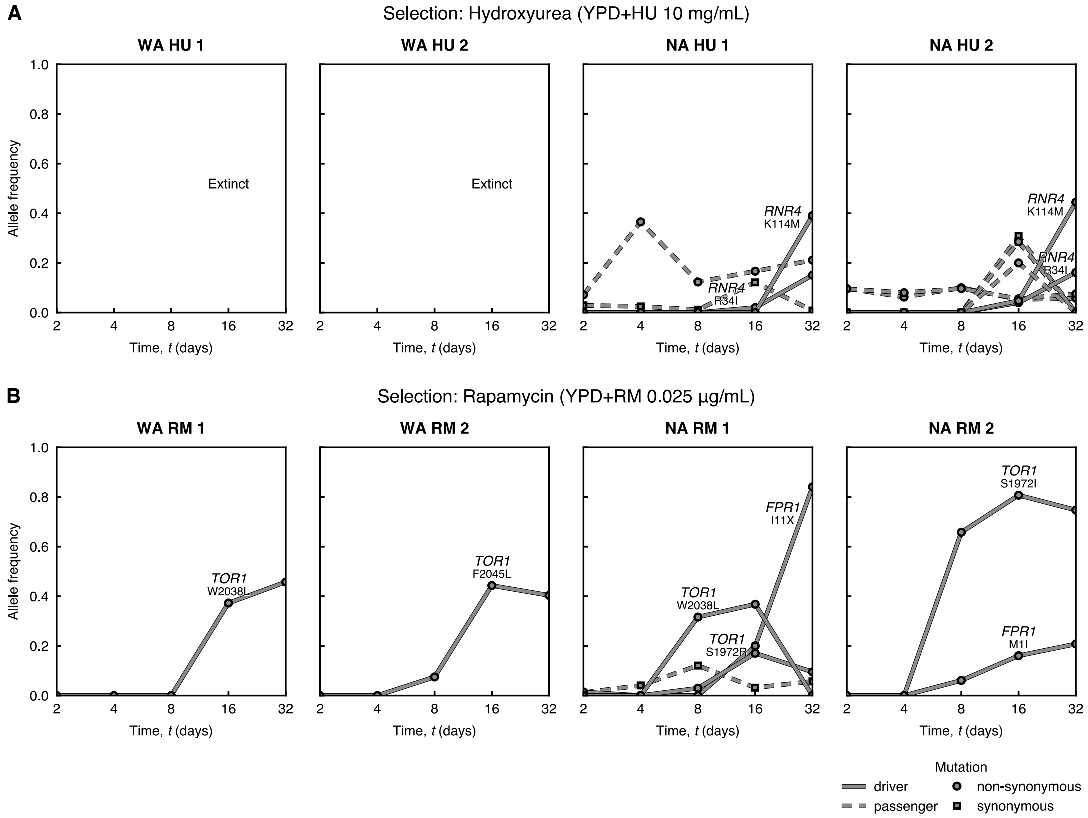

Figure S4: Subclonal dynamics in WA and NA populations. Related to Figures 3A and 3C. Subclonal dynamics in time for WA and NA founders evolved in (**A**) hydroxyurea and (**B**) rapamycin, measured by whole-population sequencing. WA founders evolved in hydroxyurea did not survive after $t = 4$ days. Driver mutations are solid lines and passenger mutations are dashed lines; circles and squares denote non-synonymous and synonymous mutations, respectively. For driver mutations, the mutated gene and codon are indicated above each line. No *de novo* mutations were detected in any of the control replicates in YPD.

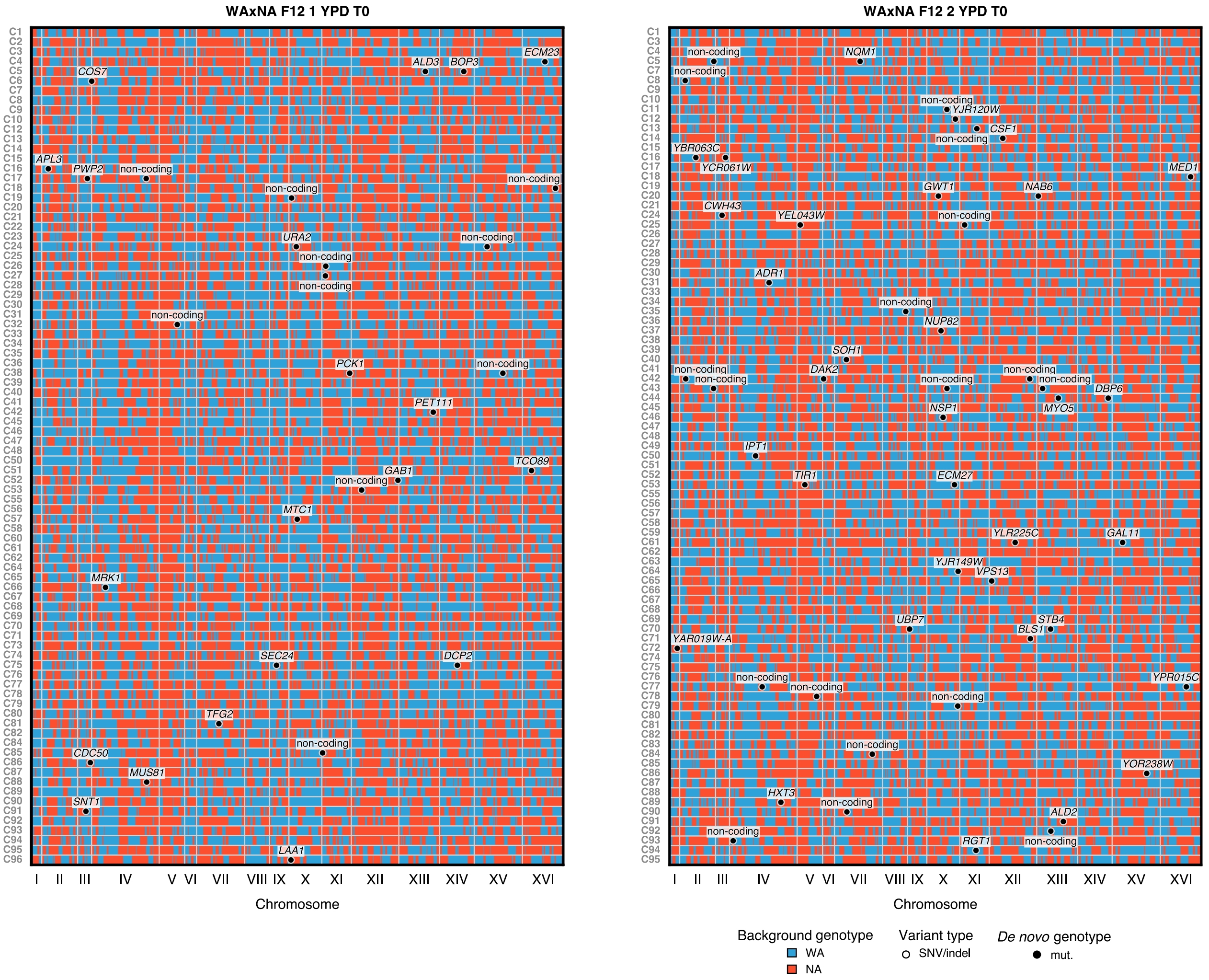

Figure S5: Genetic heterogeneity in sequences of ancestral clonal isolates. Related to Figures 4 and 5A. Whole-genome sequences of ancestral haploid clones sampled from the WAxNA F_{12} founder populations, which were obtained by bulk crossing between the WA and NA parents. Pre-existing and *de novo* SNVs and indels were detected by whole-genome sequencing in single-cell clones derived from ancestral populations at $t = 0$ days. Chromosomes are shown on the x axis; clonal isolates are listed on the left. WA (in blue) and NA (in red) represent haploid genotypes of pre-existing variants. Individual cells with unique background genotypes carry private *de novo* SNVs and indels (circles). A copy-number gain of chromosome IX (`n>2n`) was also found in clone C50 of WAxNA F12 2 YPD T0 (not shown).

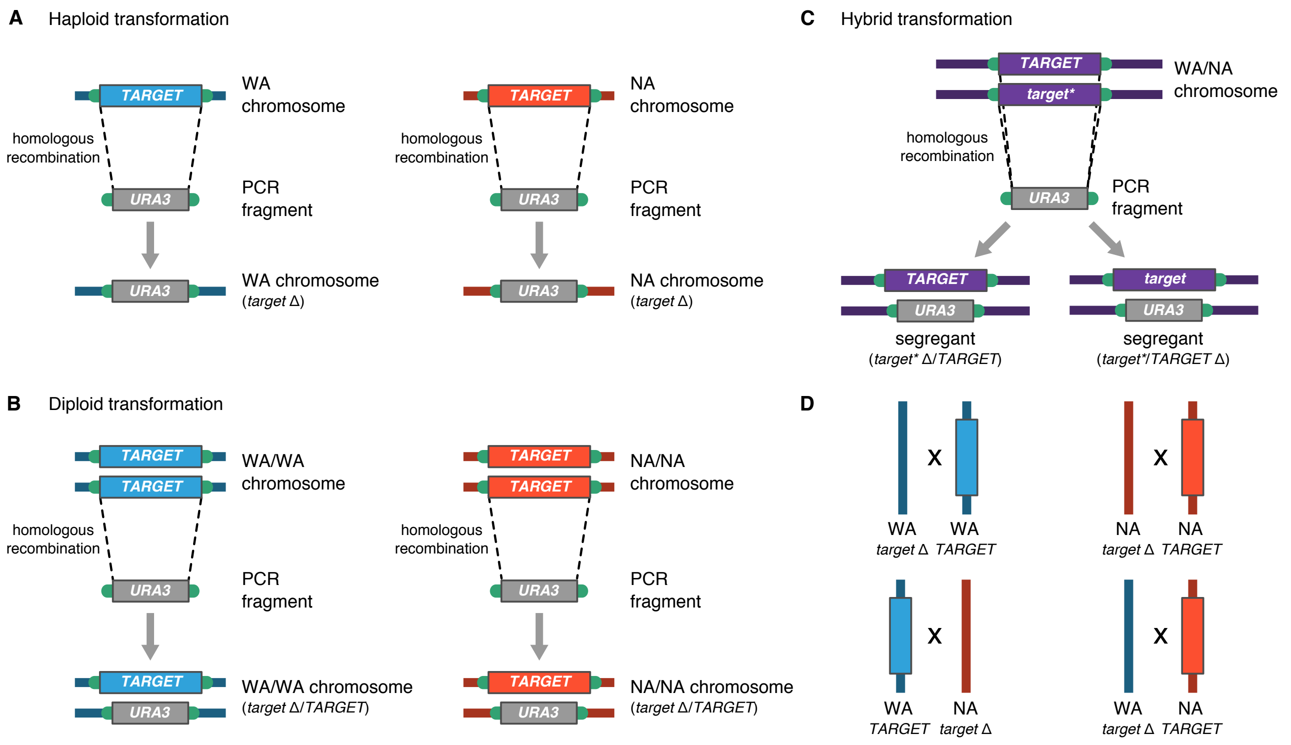

Figure S6: Strategy for strain construction. Gene deletions were mediated by homologous recombination between the terminals of the PCR product and the corresponding genomic sequence where the gene to be deleted ('target') is encoded. Blue and red lines indicate WA and NA chromosomes, respectively. Flanking regions in green indicate two different homologous sequences targeted for recombination, which are 30-40 bp long in *S. cerevisiae*. (**A**) Genes of interest were individually deleted in both WA and NA haploids, resulting in *rnr4*Δ, *fpr1*Δ and *tor1*Δ strains in both parental backgrounds. (**B**) A similar strategy was used to delete genes in WA and NA homozygous diploids. *RNR2* and *RNR4* were only deleted in one allele while there is the wild-type gene remaining in the other allele. (**C**) Evolved segregants with *de novo* mutations were isolated from the WAxNA F_{12} populations. Using the same strategy, *RNR2* or *TOR1* mutants could be rid of either the wild-type allele or the mutated allele. The primer sequences used are listed in Supplemental Tables. (**D**) We crossed the strain constructed in (A) with the parental strain with wild-type gene, to obtain strains with deleted genes in WA, NA homozygous diploid and WA/NA hybrid.

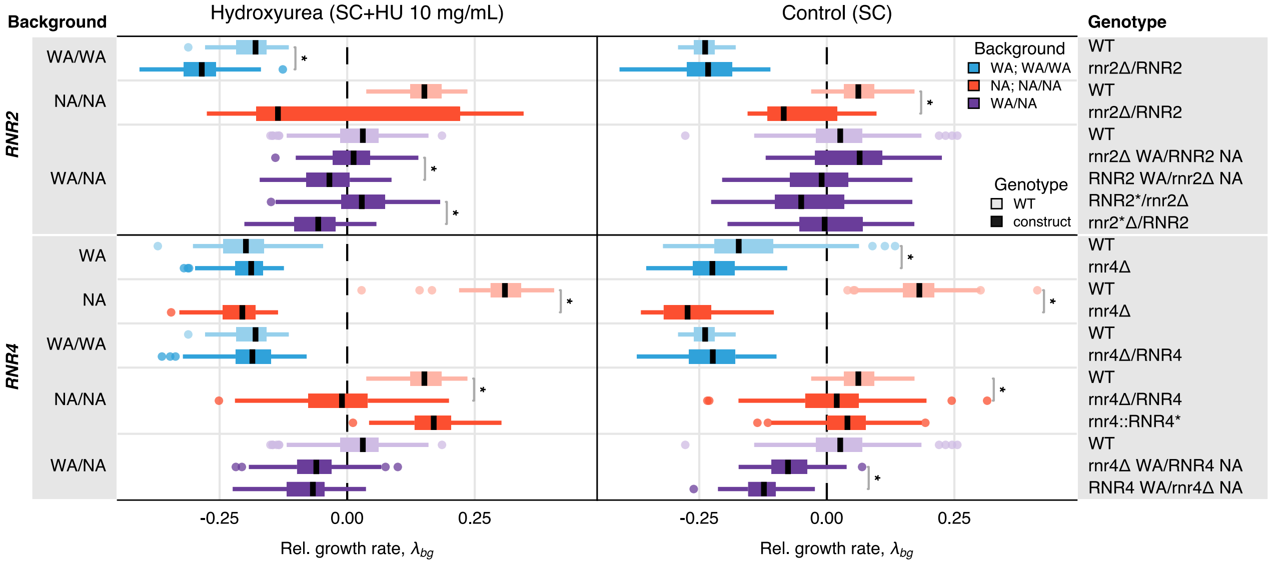

Figure S7: Validation tests for driver mutations in hydroxyurea. Validation tests for driver mutations in hydroxyurea, measured in SC+HU (left panel) and SC (right panel). The relative growth rate, λ_{bg}, of each construct is shown for $n_r = 64$ measurement replicates. Genetic constructs are grouped by candidate gene and by background of the construct, where the background b can be WA, NA (haploid); WA/WA, NA/NA (diploid); WA/NA (hybrid), and the genotype g can be wild-type for the gene, deleted or hemizygous. Relative growth rates are normalized with respect to the mean population growth rate $\langle \lambda_k \rangle_{t=0}$ at $t = 0$ days (see Figures 3B and S9A). Medians and 25%/75% percentiles are shown for each genetic construct, with medians as horizontal lines and outliers highlighted. The color of each of the boxes reflects the background (WA and WA/WA, blue; NA and NA/NA, red; WA/NA, purple). Lighter shades indicate a wild-type (WT) control for a specific background and darker shades are the candidate strains. For a given background, we compared deletion strains against their respective WT control (e.g. *rnr4*Δ vs WT in WA background) and hemizygous strains against the equivalent hemizygous strain where the opposite copy has been deleted (e.g. *rnr4*Δ WA/*RNR4* NA vs *RNR4* WA/*rnr4*Δ NA in WA/NA background). To test statistical significance we used a non-parametric Wilcoxon rank-sum test. Significance tests between two strains with $p < 10^{-4}$ are highlighted with an asterisk.

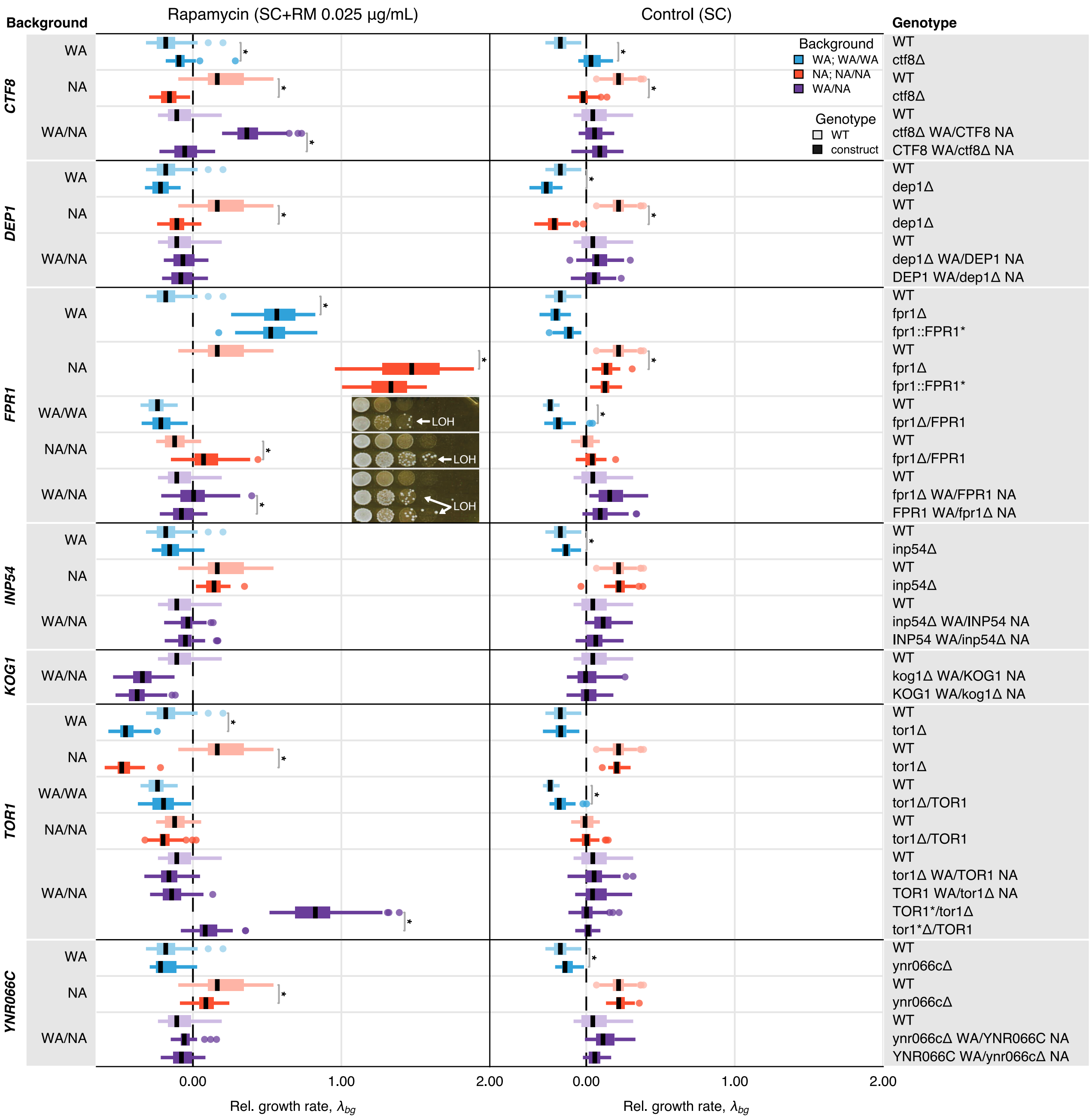

Figure S8: Validation tests for driver mutations in rapamycin. Related to Figure 2. Validation tests for driver and passenger mutations in rapamycin, measured in SC+RM (left panel) and SC (right panel). The relative growth rate, λ_{bg}, of each construct is shown for $n_r = 64$ measurement replicates. Genetic constructs are grouped by candidate gene and by background of the construct, where the background b can be WA, NA (haploid); WA/WA, NA/NA (diploid); WA/NA (hybrid), and the genotype g can be wild-type for the gene, deleted or hemizygous. Relative growth rates are normalized with respect to the mean population growth rate $\langle \lambda_k \rangle_{t=0}$ at $t = 0$ days (see Figures 3D and S9B). Medians and 25%/75% percentiles are shown for each genetic construct, with medians as horizontal lines and outliers highlighted. The color of each of the boxes reflects the background (WA and WA/WA, blue; NA and NA/NA, red; WA/NA, purple). Lighter shades indicate a wild-type (WT) control for a specific background and darker shades are the candidate strains. For a given background, we compared deletion strains against their respective WT control (e.g. *fpr1*Δ vs WT in WA background) and hemizygous strains against the equivalent hemizygous strain where the opposite copy has been deleted (e.g. *fpr1*Δ WA/*FPR1* NA vs *FPR1* WA/*fpr1*Δ NA in WA/NA background). To test statistical significance we used a non-parametric Wilcoxon rank-sum test. Significance tests between two strains with $p < 10^{-4}$ are highlighted with an asterisk. Visual inspection of *FPR1* heterozygous deletions using a spot assay (inset) manifests the immediate loss of the wild-type allele by LOH – validated by colony Sanger sequencing –.

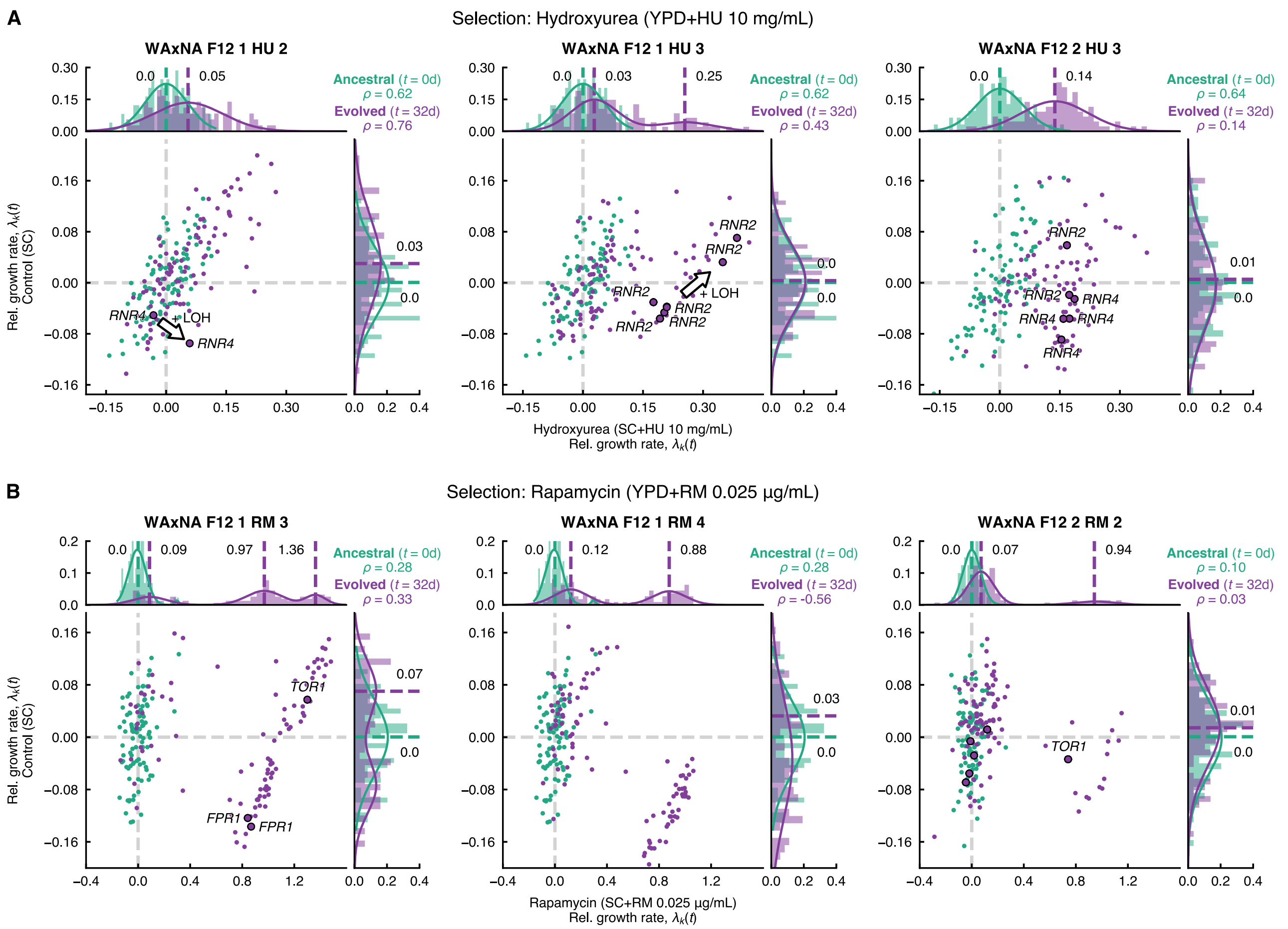

Figure S9: Variability in intra-population growth rate and fitness correlations. Related to Figures 3B and 3D. Fitness correlations of ancestral and evolved populations with and without stress, estimated by random sampling of individuals at initial ($t = 0$ days, green) and final time points ($t = 32$ days, purple), before and after selection in (**A**) hydroxyurea and (**B**) rapamycin. The relative growth rate $\lambda_k(t)$ per individual k is shown, calculated by averaging over $n_r = 32$ technical replicates per individual. The relative growth rates $\lambda_k(t)$ in the stress environment (x axis) are compared to the control environment (y axis). Relative growth rates are normalized with respect to the mean population growth rate $\langle\lambda_k\rangle_{t=0}$ at $t = 0$ days (see Figures 3B and 3D). Using a Gaussian mixture model, we found the posterior probability of the mixture modes of the the best-fit mixture (solid lines). The posterior means of the distribution modes are indicated as dashed lines. The fitter individuals carry driver mutations, as determined by targeted sampling and sequencing. Spearman's rank correlation, ρ, is shown on the top right of each panel, to assess the association between the relative growth rate of isolates in the stress and control environments at $t = 0$ and $t = 32$ days.

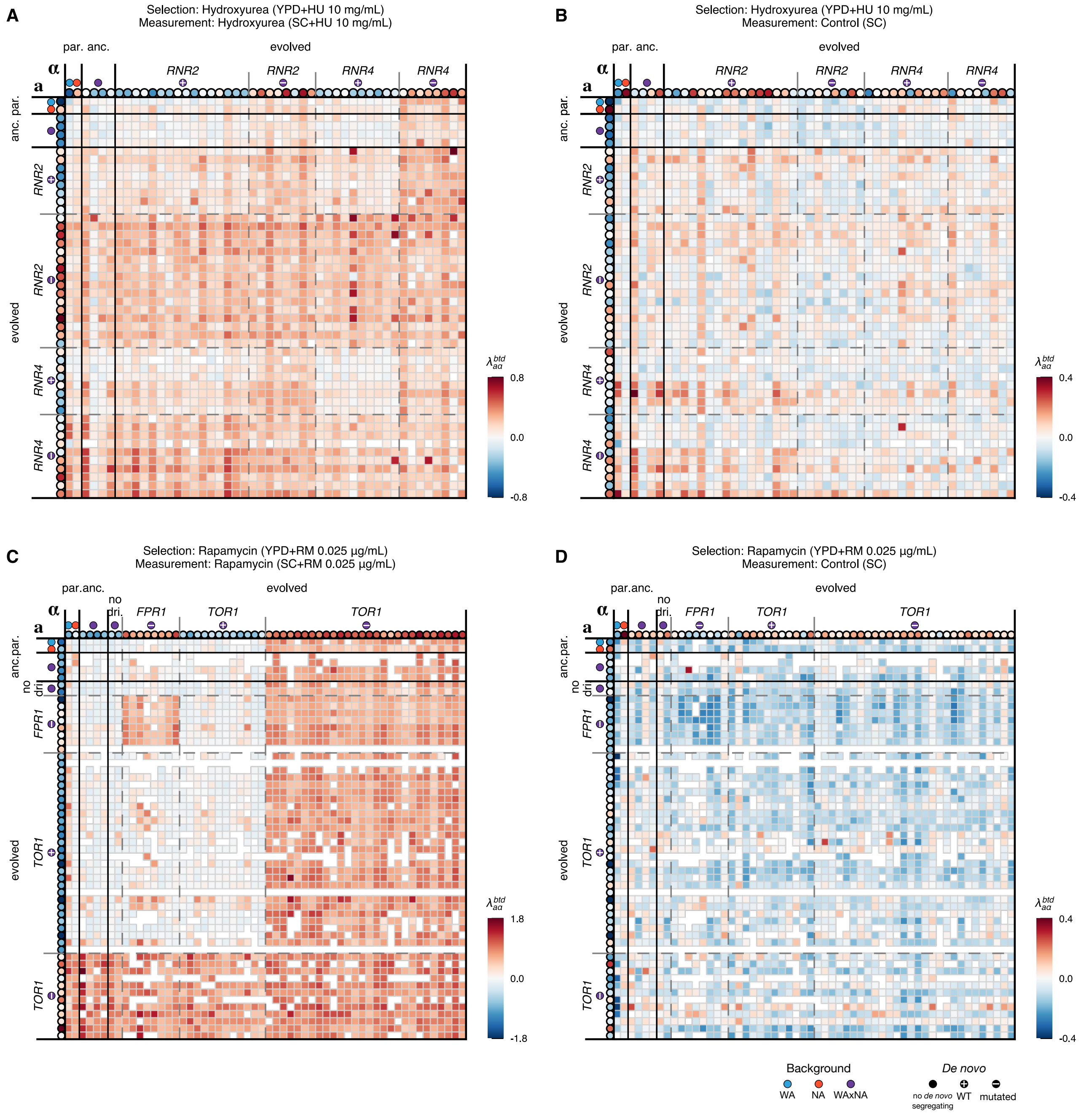

Figure S10: Fitness contribution of genetic background and *de novo* mutations. Related to Figure 6. Given an ensemble of n_b haploid spores with unique genetic backgrounds ($n_b = 48$ in hydroxyurea and $n_b = 56$ in rapamycin), every haploid spore is crossed against itself and all other haploid spores, and the two must be of opposite mating type (*MAT*a or *MAT*α) to construct a matrix of diploid hybrids of size $n_b \times n_b$. In each panel, spores are represented along the vertical and horizontal axes of the matrix and hybrids are shown as matrix elements. Symbols follow the Figure 6A legend and indicate combinations of the type of genetic background (WA parent: ●, NA parent: ●, WAxNA segregant: ●) and the genotype of *de novo* mutations (no *de novo* mutation: ●, wild-type: ⊕, mutated: ⊖). Relative growth rates of spores $\lambda^{btd}_{\{a,\alpha\}}$ and hybrids $\lambda^{btd}_{a\alpha}$ are shown, normalized with respect to the ancestral WAxNA cross. Each matrix element is labeled by background genotype b, *de novo* genotype d, and time of sampling during selection t. Measurements were taken in (**A**) SC+HU and (**B**) SC for populations selected in hydroxyurea; and (**C**) SC+RM and (**D**) SC for populations selected in rapamycin. The color scale for all matrices is shown to the right of each panel and indicates the growth rate difference with respect to the ancestral WAxNA cross. White boxes indicate missing data due to mating inefficiency and slow growth.

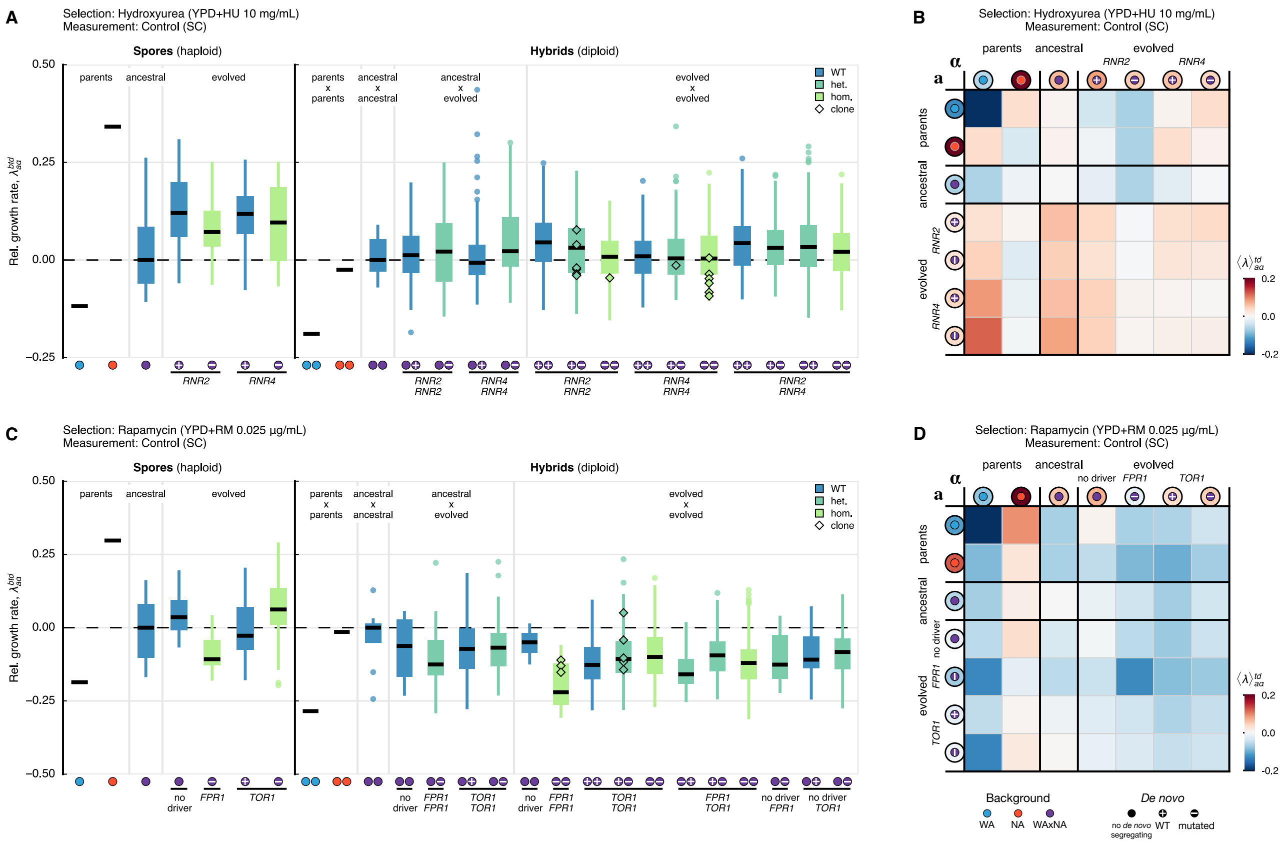

Figure S11: Ensemble average of fitness effects over genetic backgrounds. Related to Figure 6. To quantify the fitness effects of background variation and *de novo* mutations in the absence of stress, we measured the large recombinant library built with the genetic cross in a control environment (SC). Symbols follow the Figure 6A legend and indicate combinations of the type of genetic background (WA parent: ●, NA parent: ●, WAxNA segregant: ●) and the genotype of *de novo* mutations (no *de novo* mutation: ●, wild-type: ⊕, mutated: ⊖). (**A** and **C**) Relative growth rate of spores, $\lambda^{btd}_{\{a,\alpha\}}$, and hybrids, $\lambda^{btd}_{a\alpha}$, measured for multiple combinations of background and *de novo* genotypes with respect to the ancestral population, and averaged over measurement replicates. Measurements were taken in a control environment (SC) for cells selected in (A) hydroxyurea and (C) rapamycin. Medians and 25%/75% percentiles across groups are shown, with medians as horizontal lines and colored by *de novo* genotype (wild-type, blue; heterozygote, cyan; homozygote, green). Outliers (circles) and isolated, selected clones with matching genotypes (diamonds) are highlighted. (**B** and **D**) Ensemble average of the relative growth rate of spores, $\langle\lambda\rangle^{td}_{\{a,\alpha\}}$, and hybrids, $\langle\lambda\rangle^{td}_{a\alpha}$. Each matrix element is labeled by *de novo* genotype d, and time of sampling during selection t, and averaged over genetic backgrounds b. Measurements were taken in (B) hydroxyurea and (D) rapamycin. The color scale for all matrices is shown to the right of each panel and indicates the difference in the ensemble average with respect to the ancestral WAxNA cross.

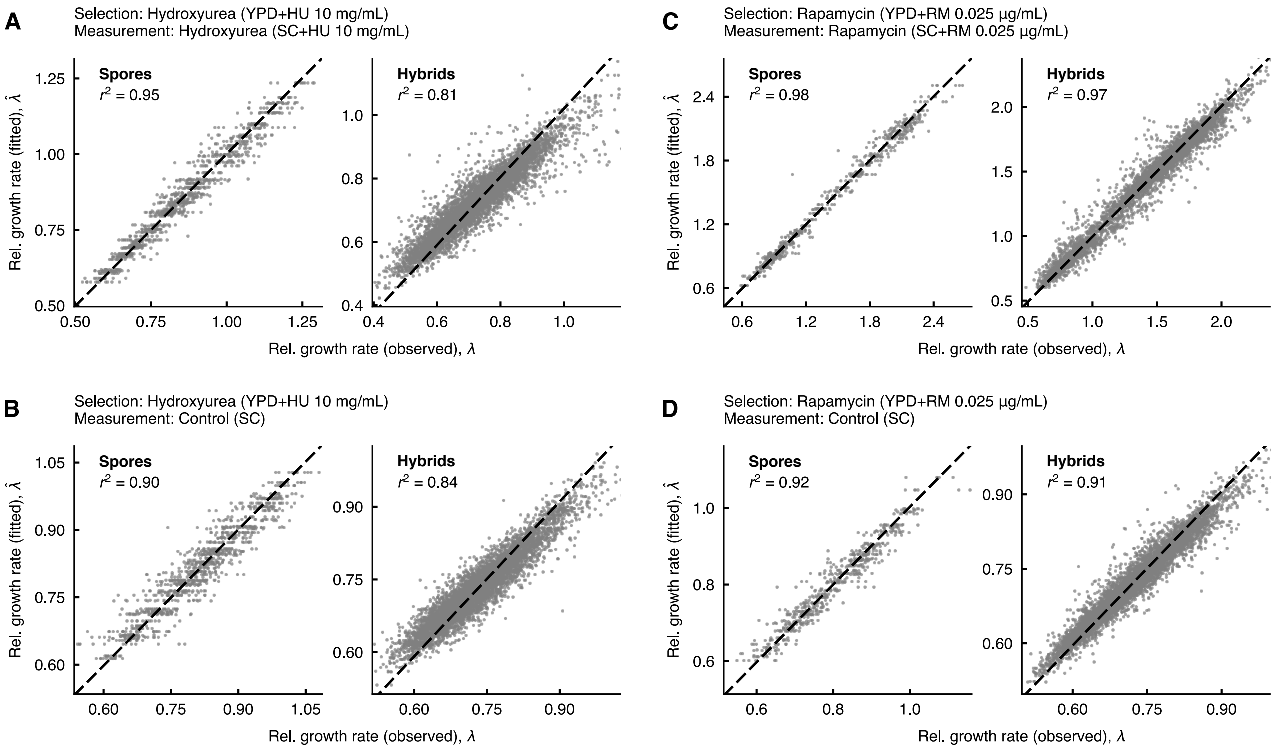

Figure S12: Hierarchical analysis of variance in the genetic cross using a linear mixed model. Related to Figure 6. We model the growth rate of spores, $\lambda^{btd}_{\{a,\alpha\}}$, and hybrids, $\lambda^{btd}_{a\alpha}$, as a function of background genotype b, *de novo* genotype d, time of sampling during selection t, and auxotrophy x. Relative growth rates are accurately fitted by this model (Model 4). Model fits are summarized in Table S2. Measurements are taken in SC+HU and SC only for populations selected in hydroxyurea (**A** and **B**); and in SC+RM and SC only for populations selected in rapamycin (**C** and **D**). The scatter shows a set of measurements λ (x axis) against the fitted rates $\hat{\lambda}$ (y axis). The total variance explained, r^2, is separately computed for spores and hybrids by environment.

Supplemental Tables

Table S1: Populations and clonal isolates analyzed by whole-genome sequencing. Related to Table 1. Summary of populations and clonal isolates analyzed by whole-genome sequencing in this study. The best-fit number of subclones N_c as estimated by cloneHD are shown together with the total clonal fraction, $F^t = \sum_{j=1}^{n} f_j^t$, after 32 days of selection. Per population, the union set of driver mutations found by whole-population and clone genome sequencing is shown. The genotypes of driver mutations found in clonal isolates were validated by Sanger sequencing (labeled by §). WA/WA populations in hydroxyurea did not survive beyond 4 days of selection (labeled by †).

Time	Background	Cross		Selection			Clonality		Drivers
		Gen.	Rep.	Environment	Rep.	Isolates	N_c	F^t	
0 days	WA/WA	–	–	YPD	–	–	–	–	
	NA/NA	–	–	YPD	–	–	–	–	
	WAxNA	F_{12}	1	YPD	–	C1–C96	–	–	
			2	YPD	–	C1–C96	–	–	
2–32 days	WA/WA	–	–	YPD+HU	1[†]	–	–	–	
					2[†]	–	–	–	
				YPD+RM	1	–	–	–	*TOR1* W2038L[§]
					2	–	–	–	*TOR1* F2045L[§]
	NA/NA	–	–	YPD+HU	1	–	–	–	*RNR4* R34I[§], K114M[§]
					2	–	–	–	*RNR4* R34I[§], K114M[§]
				YPD+RM	1	–	–	–	*FPR1* K11fs[§]; *TOR1* S1972R, W2038L[§]
					2	–	–	–	*FPR1* M1I[§]; *TOR1* S1972I[§]
	WAxNA	F_2	1	YPD+RM	1	–	2	0.74	*TOR1* W2038L
					2	–	1	0.10	
				YPD	1	–	0	–	
		F_{12}	1	YPD+HU	1	C1–C2	2	0.58	*RNR4* R34G[§], R34I[§]
					2	C1–C2	1	0.20	*RNR4* R34I[§]
					3	C1–C6	2	0.65	*RNR2* Y169H[§]; chr. II LOH
				YPD+RM	1	C1–C3	3	0.85	*CTF8*^{NA}; *FPR1* W66*[§], W66S
					2	C1–C6	2	0.20	*CTF8*^{NA}; *FPR1* W66S; *TOR1* W2038L[§]
					3	C1–C3	2	0.72	*CTF8*^{NA}; *FPR1* W66*[§]; *TOR1* S1972I
					4	–	2	0.81	*CTF8*^{NA}; *FPR1* W66*[§]
				YPD	1	–	0	–	
					2	–	0	–	
			2	YPD+HU	1	C1–C2	2	0.63	*RNR4* R34G[§], R34I[§]
					2	C1–C4	2	0.32	*RNR2* N151H, T206I[§]; *RNR4* R34I[§]
					3	C1–C6	2	0.34	*RNR2* E154G[§]; *RNR4* R34I[§]
				YPD+RM	1	C1–C3	4	0.93	*CTF8*^{NA}; *FPR1* W66S, W66*[§]
					2	C1–C6	1	0.10	*CTF8*^{NA}; *TOR1* W2038C[§]
					3	C1	1	0.10	*CTF8*^{NA}; *FPR1* S102R
					4	–	1	0.11	*CTF8*^{NA}; *FPR1* S102R
				YPD	1	–	0	–	
					2	–	0	–	

Table S2: Statistical support for variance components in the genetic cross estimated using linear mixed models. Related to Figure 6B. Summary statistics for linear mixed models of the genetic cross, fitted using restricted maximum likelihood. Models were separately fitted for spores and hybrids in each environment. The number of unique backgrounds n_b is much greater than the number of degrees of freedom (d.o.f.) for the parameters being fitted. Each background b was measured in several technical replicates. We selected the best model by maximum AIC (labeled by §). The breakdown of variance components in Model 4 is shown in Figure 6B and the models fits to the data are shown in Figure S12.

Selection	Measurement	Type	Model	d.o.f.	Variance r_F^2	r^2	Log-likelihood	AIC
Hydroxyurea	Hydroxyurea	Spores	1	3	0.0000	0.9500	1813.367	–3627.063
(YPD+HU)	(SC+HU)	$n_b = 92$	2	4	0.1071	0.9504	1816.932	–3636.219
			3§	7	0.5338	0.9507	1841.920	–3697.223
			4	8	0.5338	0.9509	1839.372	–3695.832
		Hybrids	1	3	0.0000	0.8045	6535.699	–13075.484
		$n_b = 2013$	2	5	0.0652	0.8044	6608.318	–13230.265
			3§	17	0.2989	0.8067	6969.860	–14021.351
			4	18	0.2990	0.8068	6966.279	–14019.752
Hydroxyurea	Control	Spores	1	3	0.0000	0.8914	1918.357	–3837.994
(YPD+HU)	(SC)	$n_b = 92$	2	4	0.0381	0.8924	1917.974	–3840.051
			3	7	0.0605	0.8954	1911.224	–3836.707
			4§	8	0.1559	0.8957	1913.807	–3846.480
		Hybrids	1	3	0.0000	0.8335	9435.532	–18875.965
		$n_b = 2013$	2	5	0.0009	0.8337	9429.119	–18874.079
			3	17	0.0451	0.8435	9454.950	–18998.377
			4§	18	0.0483	0.8428	9456.054	–19006.845
Rapamycin	Rapamycin	Spores	1	3	0.0000	0.9815	438.848	–875.752
(YPD+RM)	(SC+RM)	$n_b = 104$	2	4	0.0924	0.9817	443.237	–884.134
			3	7	0.8146	0.9815	524.504	–1054.421
			4§	8	0.8699	0.9815	542.282	–1094.737
		Hybrids	1	3	0.0000	0.9583	2368.301	–4738.294
		$n_b = 2271$	2	5	0.0355	0.9584	2407.246	–4819.913
			3	17	0.7422	0.9675	4465.029	–8981.497
			4§	18	0.7430	0.9675	4466.273	–8987.264
Rapamycin	Control	Spores	1	3	0.0000	0.9217	1038.794	–2078.815
(YPD+RM)	(SC)	$n_b = 104$	2	4	0.0011	0.9224	1036.569	–2076.940
			3	7	0.1711	0.9239	1039.674	–2092.692
			4§	8	0.3953	0.9235	1054.494	–2128.413
		Hybrids	1	3	0.0000	0.9065	11394.150	–22793.196
		$n_b = 2270$	2	5	0.0173	0.9066	11408.580	–22832.161
			3	17	0.0593	0.9072	11418.030	–22915.694
			4§	18	0.0697	0.9071	11428.480	–22941.867

Glossary of wild strains used in this study, including derivative strains. *Isolated in West Africa (pre-1914) by A. Guilliermond from bili wine from *Osbeckia grandiflora* (Liti et al., 2009). †Isolated in Pennsylvania (1999) by P. Sniegowski from soil beneath *Quercus alba* (Sniegowski et al., 2002).

**Background**	**ID**	**Derived from**	**Genotype**
WA	DBVPG6044	Wild isolate*	
	CC402	DBVPG6044	*MAT*a, *ura3::KanMX*, *ho::HygMX*
	CC406	DBVPG6044	*MAT*α, *ura3::KanMX*, *lys2::URA3*, *ho::HygMX*
	FS174	DBVPG6044	*MAT*α, *ura3::KanMX*, *ho::NatMX*
	YGL1001	DBVPG6044	*MAT*a/α, *ura3Δ0*; *ura3Δ0*; *leu2Δ0*; *leu2Δ0*; *lys2Δ0*; *met15Δ0*
WA/WA	CC426	CC402 × CC406	*MAT*a/α, *ura3::KanMX/ura3::KanMX*, *ho::HygMX/ho::HygMX*, *LYS2/lys2::URA3*
NA	YPS128	Wild isolate†	
	CC403	YPS128	*MAT*a, *ura3::KanMX*, *ho::HygMX*
	CC407	YPS128	*MAT*α, *ura3::KanMX*, *lys2::URA3*, *ho::HygMX*
	FS173	YPS128	*MAT*α, *ura3::KanMX*, *ho::NatMX*
	YGL1011	YPS128	*MAT*a/α, *ura3Δ0*; *ura3Δ0*; *leu2Δ0*; *leu2Δ0*; *lys2Δ0*; *met15Δ0*
NA/NA	CC440	CC403 × CC407	*MAT*a/α, *ura3::KanMX/ura3::KanMX*, *ho::HygMX/ho::HygMX*, *LYS2/lys2::URA3*
WA/NA	CC427	CC402 × CC407	*MAT*a/α, *ura3::KanMX/ura3::KanMX*, *ho::HygMX/ho::HygMX*, *LYS2/lys2::URA3*
	CC435	CC403 × CC406	*MAT*a/α, *ura3::KanMX/ura3::KanMX*, *ho::HygMX/ho::HygMX*, *LYS2/lys2::URA3*

Glossary of strains with genetic constructs used in this study. The genetic constructs are grouped by the 'target' gene of interest and are engineered in multiple genetic backgrounds. They include gene deletions, hemizygous constructs of ancestral alleles (WA and NA) and hemizygous constructs of evolved alleles.

Gene	ID	Derived from	Genotype
CTF8	YGL1269	DBVPG6044	MATa, ho::HygMX, ura3::KanMX, ctf8::URA3
	YGL1270	YPS128	MATa, ho::HygMX, ura3::KanMX, ctf8::URA3
	YGL1271	DBVPG6044 × YPS128	MATa/α, ho::HygMX, ho::NatMX, ura3::KanMX, CTF8(NA)/ctf8(wa)::URA3
	YGL1272	DBVPG6044 × YPS128	MATa/α, ho::HygMX, ho::NatMX, ura3::KanMX, CTF8(WA)/ctf8(na)::URA3
DEP1	YGL1562	DBVPG6044	MATa, ho::HygMX, ura3::KanMX, dep1::URA3
	YGL1563	YPS128	MATa, ho::HygMX, ura3::KanMX, dep1::URA3
	YGL1570	DBVPG6044 × YPS128	MATa/α, ho::HygMX, ho::NatMX, ura3::KanMX, DEP1(NA)/dep1(wa)::URA3
	YGL1571	DBVPG6044 × YPS128	MATa/α, ho::HygMX, ho::NatMX, ura3::KanMX, DEP1(WA)/dep1(na)::URA3
FPR1	YGL2166	CC402	MATa, ura3::KanMX, ho::HygMX, fpr1::URA3
	YGL2167	CC403	MATa, ura3::KanMX, ho::HygMX, fpr1::URA3
	YGL2181	FS174 × YGL2166	MATa/α, ura3::KANMX/ura3::KANMX, ho::HYGMX/ho::NATMX, fpr1::URA3/FPR1
	YGL2182	FS173 × YGL2167	MATa/α, ura3::KANMX/ura3::KANMX, ho::HYGMX/ho::NATMX, fpr1::URA3/FPR1
	YGL2184	FS173 × YGL2166	MATa/α, ura3::KANMX/ura3::KANMX, ho::HYGMX/ho::NATMX, fpr1(wa)::URA3/FPR1(NA)
	YGL2183	FS174 × YGL2167	MATa/α, ura3::KANMX/ura3::KANMX, ho::HYGMX/ho::NATMX, fpr1(na)::URA3/FPR1(WA)
	YGL2175	YGL2166	MATa, ura3::KanMX, ho::HygMX, fpr1::FPR1*
	YGL2193	YGL2167	MATa, ura3::KANMX, ho::HYGMX, fpr1::FPR1*
INP54	YGL1564	DBVPG6044	MATa, ho::HygMX, ura3::KanMX, inp54::URA3
	YGL1565	YPS128	MATa, ho::HygMX, ura3::KanMX, inp54::URA3
	YGL1572	DBVPG6044 × YPS128	MATa/α, ho::HygMX, ho::NatMX, ura3::KanMX, INP54(NA)/inp54(wa)::URA3
	YGL1573	DBVPG6044 × YPS128	MATa/α, ho::HygMX, ho::NatMX, ura3::KanMX, INP54(WA)/inp54(na)::URA3
KOG1	YGL1264	DBVPG6044 × YPS128	MATa/α, ho::HygMX, ho::NatMX, ura3::KanMX, KOG1(NA)/kog1(wa)::URA3
	YGL1263	DBVPG6044 × YPS128	MATa/α, ho::HygMX, ho::NatMX, ura3::KanMX, KOG1(WA)/kog1(na)::URA3
RNR2	YGL2164	DBVPG6044	MATa/α, ura3Δ0, ura3Δ0, leu2Δ0, leu2Δ0, lys2Δ0, met15Δ0, RNR2/rnr2::URA3
	YGL2165	YPS128	MATa/α, ura3Δ0, ura3Δ0, leu2Δ0, leu2Δ0, lys2Δ0, met15Δ0, RNR2/rnr2::URA3
	YGL2391	CC427	MATa/α, ura3::KanMX/ura3::KanMX, ho::HygMX/ho::HygMX, LYS2/lys2::URA3, RNR2(NA)/rnr2(wa)::NATMX
	YGL2392	CC427	MATa/α, ura3::KanMX/ura3::KanMX, ho::HygMX/ho::HygMX, LYS2/lys2::URA3, RNR2(WA)/rnr2(na)::NATMX
	YGL2198	WAxNA F12 2 HU 2 T32 C2	MATa/α, ura3::KanMX/ura3::KanMX, LYS2/lys2::URA3, rnr2*::NATMX/RNR2
	YGL2189	WAxNA F12 2 HU 2 T32 C2	MATa/α, ura3::KanMX/ura3::KanMX, LYS2/lys2::URA3, rnr2*/RNR2::NATMX
RNR4	YGL2174	CC402	MATa, ura3::KanMX, ho::HygMX, rnr4::URA3, aneuploidy in chr. VII (w/ *RNR4*)
	YGL2170	CC403	MATa, ura3::KanMX, ho::HygMX, rnr4::URA3
	YGL2177	FS174 × YGL2174	MATa/α, ura3::KANMX/ura3::KANMX, ho::HYGMX/ho::NATMX, rnr4::URA3/RNR4
	YGL2178	FS173 × YGL2170	MATa/α, ura3::KANMX/ura3::KANMX, ho::HYGMX/ho::NATMX, rnr4::URA3/RNR4
	YGL2180	FS173 × YGL2174	MATa/α, ura3::KANMX/ura3::KANMX, ho::HYGMX/ho::NATMX, rnr4::URA3/RNR4
	YGL2179	FS174 × YGL2170	MATa/α, ura3::KANMX/ura3::KANMX, ho::HYGMX/ho::NATMX, rnr4::URA3/RNR4
	YGL2194	YGL1001	MATa/α, ura3Δ0; ura3Δ0; leu2Δ0; leu2Δ0; lys2Δ0; met15Δ0; RNR4(WA)/rnr4(wa)::URA3
	YGL2196	YGL1011	MATa/α, ura3Δ0; ura3Δ0; leu2Δ0; leu2Δ0; lys2Δ0; met15Δ0; RNR4(NA)/rnr4(na)::URA3
	YGL2176	YGL2170	MATa, ura3::KanMX, ho::HygMX, rnr4::RNR4*
TOR1	YGL2168	CC402	MATa, ura3::KanMX, ho::HygMX, tor1::URA3
	YGL2169	CC403	MATa, ura3::KanMX, ho::HygMX, tor1::URA3
	YGL2185	FS174 × YGL2168	MATa/α, ura3::KANMX/ura3::KANMX, ho::HYGMX/ho::NATMX, tor1::URA3/TOR1
	YGL2186	FS173 × YGL2169	MATa/α, ura3::KANMX/ura3::KANMX, ho::HYGMX/ho::NATMX, tor1::URA3/TOR1
	YGL2188	FS173 × YGL2168	MATa/α, ura3::KANMX/ura3::KANMX, ho::HYGMX/ho::NATMX, tor1(wa)::URA3/TOR1(NA)
	YGL2187	FS174 × YGL2169	MATa/α, ura3::KANMX/ura3::KANMX, ho::HYGMX/ho::NATMX, tor1(na)::URA3/TOR1(WA)
	YGL2201	WAxNA F12 2 RM 2 T32 C6	MATa/α, ura3::KanMX/ura3::KanMX, LYS2/lys2::URA3, tor1*::NATMX/TOR1
	YGL2191	WAxNA F12 2 RM 2 T32 C6	MATa/α, ura3::KanMX/ura3::KanMX, LYS2/lys2::URA3, tor1*/TOR1::NATMX
YNR066C	YGL1566	DBVPG6044	MATa, ho::HygMX, ura3::KanMX, ynr066c::URA3
	YGL1567	YPS128	MATa, ho::HygMX, ura3::KanMX, ynr066c::URA3
	YGL1574	DBVPG6044 × YPS128	MATa/α, ho::HygMX, ho::NatMX, ura3::KanMX, YNR066C/ynr066c::URA3
	YGL1575	DBVPG6044 × YPS128	MATa/α, ho::HygMX, ho::NatMX, ura3::KanMX, YNR066C/ynr066c::URA3

Primers for amplification of putative drivers. Sequence of primers used to amplify the genes containing putative driver mutations.

Gene	Orientation	Chr	Target Start	Target End	Sequence (TARGET)
DEP1	fwd	I	128977	128996	CAGAGAGCTGGTCCAGTTCA
	rev	I	129573	129554	TGGCCTCATCTATCGCCTCT
FPR1	fwd	XIV	371600	371619	CCCTCCTGCCACAAGAGTTT
	rev	XIV	372170	372151	TGCCACCTTCCCAAAGACAG
INP54	fwd	XV	205005	205024	GCGAAAGTTGGCACTGCATA
	rev	XV	205624	205605	GCTACACAAGGGGATGAGCA
RNR2	fwd	X	392707	392726	CGTGCCGAAGCTTCTTTCTG
	rev	X	393245	393226	CATGCAAAGTCGGTGTGCAA
RNR4	fwd	VII	855968	855987	CAGGGTTTTGCAATTGGGCA
	rev	VII	856843	856824	TACGACCACCCAACACCAAG
TOR1	fwd	X	565069	565088	AGCCAGATCCTACGGTGAGT
	rev	X	565652	565633	CCCAGGAACAGCCAATTCGA
YNR066C	fwd	XIV	753660	753679	TCGAATTCACTACCGTCGCC
	rev	XIV	754369	754348	GCCGCATATACACAATTAGCCT

Primers used to engineer genetic constructs. Sequence of primers used to engineer gene deletions (see 'Engineered genetic constructs').

Gene	Marker	Orientation	Chr	Target Start	End	Sequence (TARGET, *ura3*/*natMX*)
CTF8	*URA3*	fwd	VIII	486155	486230	TATATACACTTTACACAGAGCGTGAAGTCTGCGCCAAATAACATAAACAAACAACTC<br>CGAACAATAACTAAGTACTcggcatcagagcagattgtactg
	URA3	rev	VIII	486709	486631	CTAACCACTAATATAGCCAAAGGAGTGATAGAAAAAAGAATTATCACTATCATTCAG<br>CCCAATAAACAGCTGAAAAGAAacaccgcagggtaataactg
DEP1	*URA3*	fwd	I	129210	129269	AACGGCAAAGTACAAGGGAAGGAAGCACAGAAGCAAGAGGAGGCGCATCGATCGTGG<br>CAGcggcatcagagcagattgtactg
	URA3	rev	I	130547	130488	ATAGCGTTACAACATATTTAAGAATAACAAAAAGAAGTGGTATGGGGTCCAGTGTGG<br>CGGacaccgcagggtaataactg
FPR1	*URA3*	fwd	XIV	371821	371881	GATACTTACCATAAACATAAATAAAAAGCAGAAAGGCGGCTCAATTGATAGTACTTT<br>GCTTacaccgcagggtaataactg
	URA3	rev	XIV	372287	372227	TAAAGTAAGGCCTTTCACCTAAACTCGAGTATAAGCAAAAAATCAATCAAAACAAGT<br>AATAcggcatcagagcagattgtactg
INP54	*URA3*	fwd	XV	204671	204730	ACTGACGTTATCTGTTTCAGACATAAATGAAAAACTTCTAGCCTGACAGCCCAGATC<br>ACTcggcatcagagcagattgtactg
	URA3	rev	XV	205945	205886	TAAGAGTAGGCTAACAAAGAAGAAAAGTGAGACAAGAAAATACAGCAGGATTCTGAC<br>CGAacaccgcagggtaataactg
KOG1	*URA3*	fwd	VIII	475924	475999	TAATAGATTATATATATATATATATATATATCTCTTTTGCAGCTAAATGAAAGAAAA<br>AAAAAGAAATGGCACATATcggcatcagagcagattgtactg
	URA3	rev	VIII	480750	480672	GAATGCATTTGGTTTGTAGATTCCTTTGATTACATTTAGCGAATCCTATTGCATGCA<br>GAGAAGGGTAAAAGATACATAAacaccgcagggtaataactg
RNR2	*URA3*	fwd	X	392343	392403	CTCGATTGGCTATCTACCAAAGAATCCAAACTTAATACACGTATTTATTTGTCCAAT<br>TACCcggcatcagagcagattgtactg
	URA3	rev	X	393664	393604	CGAAAGCCCACATAAAGAGATTGAAGAGACTGCGTAAAAAGAAATATATAGAGAGAT<br>ACTCacaccgcagggtaataactg
	NatMX	fwd	X	392343	392403	CTCGATTGGCTATCTACCAAAGAATCCAAACTTAATACACGTATTTATTTGTCCAAT<br>TACCcgtacgctgcaggtcgac
	NatMX	rev	X	393604	393664	CGAAAGCCCACATAAAGAGATTGAAGAGACTGCGTAAAAAGAAATATATAGAGAGAT<br>ACTCatcgatgaattcgagctcg
RNR4	*URA3*	fwd	VII	855203	855263	TATATATAAATATATATAAATAAAAGTGGCCAAGAATAAAAGAACGCACCCCGTCGT<br>TGACacaccgcagggtaataactg
	URA3	rev	VII	856362	856302	TACAAAAACAGATCTTTTTGAGCCACACAACCCCGCGCAACGCACACAATTAGTTAT<br>TACAcggcatcagagcagattgtactg
TOR1	*URA3*	fwd	X	559355	559415	TCACGAGAGAGTCATTGGTAAAGTGAAACATACATCAACCGGCTAGCAGGTTTGCAT<br>TGATcggcatcagagcagattgtactg
	URA3	rev	X	566889	566829	AATGCGTAATACAAAAAAAATAAATAGTAAACAAAGCACGAAATGAAAAATGACACC<br>GCAGacaccgcagggtaataactg
	NatMX	fwd	X	559355	559415	TCACGAGAGAGTCATTGGTAAAGTGAAACATACATCAACCGGCTAGCAGGTTTGCAT<br>TGATcgtacgctgcaggtcgac
	NatMX	rev	X	566829	566889	AATGCGTAATACAAAAAAAATAAATAGTAAACAAAGCACGAAATGAAAAATGACACC<br>GCAGatcgatgaattcgagctcg
YNR066C	*URA3*	fwd	XIV	753665	753724	TTCACTACCGTCGCCAACGGAACCTGTCATTAACATAATTCCGGCAGTAGGATTTGA<br>GATcggcatcagagcagattgtactg
	URA3	rev	XIV	755095	755036	ATAAAGTTCCGAGCTTTGAAAAAAAGCTTTGAACTAAGAAAAGGTAAGAGATCCTCA<br>ATTacaccgcagggtaataactg

Supplemental References

Barbera, M. A. and Petes, T. D. (2006). Selection and analysis of spontaneous reciprocal mitotic cross-overs in Saccharomyces cerevisiae. Proc. Natl. Acad. Sci. U.S.A. *103*, 12819–12824.

Bates, D., Maechler, M., Bolker, B. M., and Walker, S. (2015). Fitting linear mixed-effects models using lme4. J. Stat. Soft. *67*, 1–48.

DePristo, M. A. et al. (2011). A framework for variation discovery and genotyping using next-generation DNA sequencing data. Nat. Genet. *43*, 491–498.

Fischer, A., Vázquez-García, I., Illingworth, C. J., and Mustonen, V. (2014). High-definition reconstruction of clonal composition in cancer. Cell Rep. *7*, 1740–1752.

Gelman, A. and Hill, J. (2006). *Data analysis using regression and multilevel/hierarchical models*. Cambridge University Press.

Gerke, J., Lorenz, K., and Cohen, B. (2006). Genetic interactions between transcription factors cause natural variation in yeast. Science *323*, 498–501.

Hamon, A. and Ycart, B. (2012). Statistics for the Luria-Delbrück distribution. Electron. J. Stat. *6*, 1251–1272.

Illingworth, C. J. R., Parts, L., Bergström, A., Liti, G., and Mustonen, V. (2013). Inferring genome-wide recombination landscapes from advanced intercross lines: application to yeast crosses. PLoS One *8*, 1–10.

Illingworth, C. J. R., Parts, L., Schiffels, S., Liti, G., and Mustonen, V. (2012). Quantifying selection acting on a complex trait using allele frequency time series data. Mol. Biol. Evol. *29*, 1187–1197.

Koser, P. L. et al. (1993). The tyrosine89 residue of yeast FKBP12 is required for rapamycin binding. Gene *129*, 159–165.

Lang, G. I. and Murray, A. W. (2008). Estimating the per-base-pair mutation rate in the yeast Saccharomyces cerevisiae. Genetics *178*, 67–82.

Li, H. (2011). A statistical framework for SNP calling, mutation discovery, association mapping and population genetical parameter estimation from sequencing data. Bioinformatics *27*, 2987–2993.

Li, H. and Durbin, R. (2009). Fast and accurate short read alignment with Burrows-Wheeler transform. Bioinformatics *25*, 1754–1760.

Liti, G. et al. (2009). Population genomics of domestic and wild yeasts. Nature *458*, 337–341.

Lorenz, M. C. and Heitman, J. (1995). TOR mutations confer rapamycin resistance by preventing interaction with FKBP12-rapamycin. J. Biol. Chem. *270*, 27531–27537.

Lunter, G. and Goodson, M. (2011). Stampy: A statistical algorithm for sensitive and fast mapping of Illumina sequence reads. Genome Res. *21*, 936–939.

Luria, S. E. and Delbrück, M. (1943). Mutations of bacteria from virus sensitivity to virus resistance. Genetics *28*, 491–511.

Rimmer, A., Phan, H., Mathieson, I., Iqbal, Z., Twigg, S. R. F., Wilkie, A. O. M., McVean, G., and Lunter, G. (2014). Integrating mapping-, assembly- and haplotype-based approaches for calling variants in clinical sequencing applications. Nat. Genet. *46*, 912–918.

Sniegowski, P. D., Dombrowski, P. G., and Fingerman, E. (2002). Saccharomyces cerevisiae and Saccharomyces paradoxus coexist in a natural woodland site in North America and display

different levels of reproductive isolation from European conspecifics. FEMS Yeast Res. *1*, 299–306.

Zackrisson, M. et al. (2016). Scan-o-matic: High-resolution microbial phenomics at a massive scale. G3 Genes—Genomes—Genetics *6*, 3003–3014.

Zheng, X. F. and Schreiber, S. L. (1997). Target of rapamycin proteins and their kinase activities are required for meiosis. Proc. Natl. Acad. Sci. U.S.A. *94*, 3070–3075.
